# Cross-bridge model for predicting muscle short-range stiffness during movement

**DOI:** 10.1371/journal.pcbi.1014748

**Published:** 2026-09-01

**Authors:** Tim J. van der Zee, Surabhi N. Simha, Gregory N. Milburn, Kenneth S. Campbell, Lena H. Ting, Friedl De Groote

**Affiliations:** 1 Department of Movement Sciences, KU Leuven, Leuven, Belgium; 2 Wallace H. Coulter Department of Biomedical Engineering, Emory University and The Georgia Institute of Technology, Atlanta, Georgia, United States of America; 3 Division of Cardiovascular Medicine, University of Kentucky, Lexington, Kentucky, United States of America; 4 Department of Rehabilitation Medicine, Division of Physical Therapy, Emory University, Atlanta, Georgia, United States of America; University of South Australia, AUSTRALIA

## Abstract

Musculoskeletal simulations can offer valuable insight into how the properties of our musculoskeletal system influence the biomechanics of our daily movements. One such property is muscle’s initial resistance to stretch, also known as short-range stiffness, which is key to stabilizing movements in response to external perturbations. Short-range stiffness is poorly captured by existing musculoskeletal simulations since they employ phenomenological Hill-type models lacking activation-dependent stiffness properties. Existing simulations also do not capture the history-dependent reduction in short-range stiffness after muscle shortening, known as muscle thixotropy. While cross-bridge models can reproduce muscle short-range stiffness, it remains unclear which model properties are necessary to capture its history dependence. Here, we tested the ability of various cross-bridge models to reproduce empirical short-range stiffness and its history-dependent changes across a broad range of behaviorally relevant length changes and activation levels, using an existing dataset on 11 permeabilized rat soleus muscle fibers. We quantified muscle thixotropy using the ratio between the observed short-range stiffnesses after and before shortening. We computed the root-mean-square deviation ($\sigma_{SRS}$) between the predicted short-range stiffness ratio of various muscle models and the measured stiffness ratio. We found that cross-bridge models captured short-range stiffness changes across conditions with both small and large history-dependent stiffness reductions ($\sigma_{SRS}$ ≤ 0.1), but only when including cooperative activation of both thin and thick myofilaments. In contrast, Hill-type models and a cross-bridge model without cooperative myofilament activation underestimated short-range stiffness and did not capture its change across conditions with large history-dependent stiffness reductions ($\sigma_{SRS}$ > 0.2). Similar results were obtained when using a Gaussian-approximated solution method to simulate the cross-bridge distribution, but at an approximately eightfold lower computational cost. We therefore propose to implement Gaussian-approximated cross-bridge models with cooperative myofilament activation into musculoskeletal simulations to improve the prediction of short-range stiffness during movements.

## Author summary

Computer simulations based on musculoskeletal models can help us understand how muscles support and control everyday movements such as walking. One important muscle feature is its initial resistance to stretch, which helps stabilize the body when it is suddenly perturbed. This initial resistance is considerably reduced after muscle shortening, facilitating subsequent movements. Current musculoskeletal simulations rely on ‘Hill-type’ muscle models that do not capture muscle’s initial resistance to stretch nor its reduction after shortening. Here, we evaluated muscle forces during stretch and shortening predicted by both Hill-type models and ‘cross-bridge’ models of muscle’s contractile machinery. We compared model forces to those measured from isolated rat soleus muscle fibers and found that only cross-bridge models with cooperative interactions between muscle’s thin and thick filaments could accurately predict both muscle’s initial resistance to stretch and its reduction after shortening. Simpler models consistently underestimated muscle’s initial resistance to stretch and its reduction after shortening at submaximal activation levels. We also showed that a simplified mathematical solution method could produce similar results ~8 times faster, making these detailed models practical for larger musculoskeletal simulations. Incorporating these cooperative cross-bridge muscle models could lead to more realistic predictions of muscle behavior in everyday movements.

## Introduction

**Fig 1:**
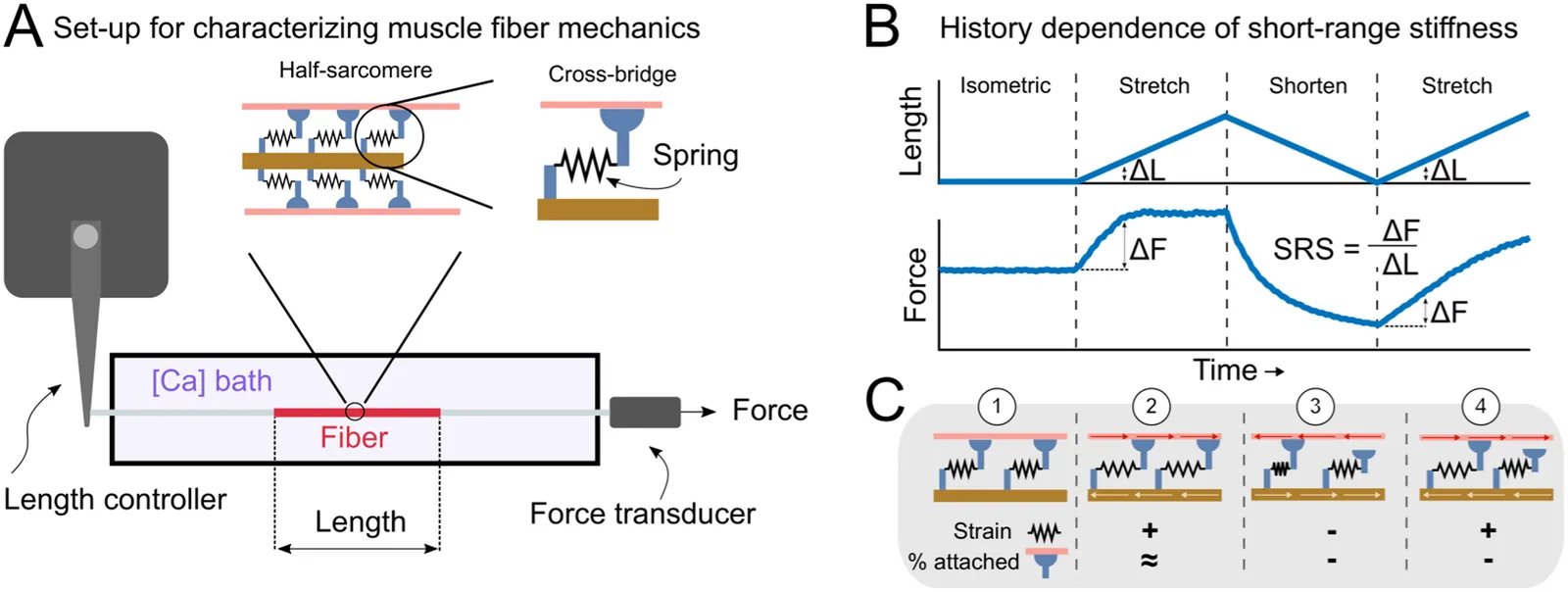
Muscle thixotropy is a history-dependent reduction in muscle short-range stiffness. **A.** Experimental set-up used for characterizing single fiber muscle mechanics. A single permeabilized muscle fiber is attached to a length controller on one end and a force transducer at the other. Muscle activation is controlled using a bathing solution with a fixed calcium (Ca^2+^) concentration. **B.** Typical example data from a muscle mechanics experiment in which triangular length changes were imposed on an isolated muscle fiber. When the fiber has been isometric for a while, force rapidly increases following onset of the first stretch (ΔF). But when the fiber is shortened and stretched again, the force increase ΔF is smaller for the same length increase ΔL. **C.** The history dependence of short-range stiffness may be explained by a cross-bridge mechanism. Molecular cross-bridges are thought to act as springs when attached (1) that get strained during stretch (2), and either unstrained or detached during shortening (3). When another stretch occurs shortly after shortening, some detached cross-bridges will not have had the time to reattach. This mechanism would explain why force increases less rapidly during the second stretch compared to during the first stretch.

Skeletal muscles are both the motors and the brakes of our musculoskeletal system [1]. Muscles’ ability to act as a brake is especially important for stabilizing movements in response to perturbations, reducing the need for sensorimotor feedback [2–6]. Short-range stiffness, a rapid and transient rise in muscle force upon stretch, is a movement history-dependent muscle property that contributes to a muscle’s braking ability [7]. Most of this rapid increase in force is thought to arise from cross-bridge stretching, i.e., cross-bridges act as stiff springs (Fig 1) [8,9]. Prior muscle shortening results in a subsequent reduction in short-range stiffness that is known as muscle thixotropy [10]. This stiffness reduction after shortening is consistent with a cross-bridge mechanism, as shortening is thought to reduce the number of formed cross-bridges [11,12] (Fig 1). Indeed, muscle models of cross-bridge cycling can qualitatively reproduce short-range stiffness and muscle thixotropy [9], while phenomenological Hill-type models require phenomenological extensions to do so [13,14]. Previously proposed extensions only distinguish the absence or presence of prior movement, and therefore cannot describe the complex relationship between muscle short-range stiffness and movement history. Yet, also simple cross-bridge models may be insufficient to capture the activation dependence of muscle thixotropy [8–10,15]. We therefore aimed to identify a cross-bridge muscle model that could capture alterations in force due to behaviorally-relevant muscle length changes, and especially short-range stiffness and its history dependence at a range of activation levels measured in vitro [16]. Alongside cross-bridge models, we also tested Hill-type models as a benchmark, as they remain the most commonly employed type of muscle model in musculoskeletal simulations of human movement [17]. As cross-bridge models are more computationally intensive than Hill-type models [18], we also sought to improve the computational efficiency of the resulting model.

**Movement history-dependent changes in muscle short-range stiffness, known as muscle thixotropy, may be shaped by cooperative myofilament dynamics that regulate cross-bridge cycling.** The rate of cross-bridge cycling depends on cross-bridge attachment and detachment rates. The attachment rate is proportional to the product of the number of available myosin heads and the number of active thin filament binding sites. The detachment rate is proportional to the number of formed cross-bridges. Cross-bridge cycling is thought to be cooperative in at least two ways. First, at low activation levels, the activation rate of the thin filament increases with the number of active thin filament binding sites (i.e., sites that can bind cross-bridges) [19]. This “thin filament cooperativity” is mediated by the formation of cross-bridges, as thin filament binding sites that have a head bound cannot deactivate [20,21]. Second, myosin heads are thought to transition from a ‘disordered-relaxed state’ or ‘ON state’ in which they can bind to actin, to a ‘super-relaxed state’ or ‘OFF state’ in which they cannot [22]. This transition is thought to depend on force, as filament stress may break the molecular interactions that stabilize the super-relaxed state, thereby decreasing the relative free energy of the disordered-relaxed state [23]. Cross-bridge force therefore influences the fraction of cross-bridges in the disordered-relaxed state (i.e., thick filament activation), a process referred to as “thick filament cooperativity”. As muscle shortening is thought to result in a reduction in both the number of formed cross-bridges [11,12] and the force per cross-bridge [12,24] (Fig 1C), it affects both thin and thick filament activation. Both thin and thick filament cooperativity may thereby contribute to muscle thixotropy.

**Muscle models may need cooperative myofilament dynamics to capture muscle thixotropy at submaximal activation**. While cross-bridge models lacking cooperative dynamics capture the history-dependence of short-range stiffness at maximal calcium activation levels, they cannot reproduce the empirical observation that muscle thixotropy is greatest at low activation levels [8,9]. Considering that cooperative mechanisms explain the activation dependence of muscle’s rate of force development [25], they might also explain the activation dependence of muscle thixotropy. Some cross-bridge models incorporate cooperative dynamics, but such models have not been tested for predicting short-range stiffness across a broad range of conditions including activation levels and recovery times [10,15,20,26–32]. Since cooperative myofilament activation is inherently coupled to cross-bridge cycling [10,21–23], it is not straightforward to add this to phenomenological models lacking cross-bridges. Hill-type models have been extended to better capture muscle thixotropy, but these models require the movement history to be set manually and do not describe the effect of recovery time and muscle activation on muscle short-range stiffness [14,33]. Therefore, we assessed whether cross-bridge models with cooperative myofilament activation can capture short-range stiffness and its history dependence across a range of behaviorally relevant conditions.

**Muscle models may require an additional detached cross-bridge state to explain short-range stiffness.** Early cross-bridge models [34,35] capture steady-state force-velocity curves, while only simulating two cross-bridge states: attached or detached. However, these simple models poorly captured force transients during stretch [36,37]. More specifically, these models predict a large increase in force during stretch followed by a decay, while empirical forces initially rise during stretch and plateau with further lengthening [38]. More complex cross-bridge models sometimes include a ‘forcibly-detached’ cross-bridge state that can be entered when cross-bridges are stretched beyond a ‘critical stretch’ [39]. Such a forcibly-detached state is thought to be associated with a faster re-attachment rate, that might explain the plateau phase of the force response. However, it remains unclear whether the inclusion of a forcibly-detached cross-bridge state is required to explain short-range stiffness and its history dependence. Therefore, we explored the effect of including a forcibly-detached cross-bridge state on the ability to capture short-range stiffness.

**Approximating the cross-bridge distribution with a Gaussian may reduce computational cost, but the trade-off between accuracy and computational cost needs to be examined for more complex cross-bridge models.** Existing cross-bridge muscle models vary in the way they describe the strain distribution of formed cross-bridges. Some solution methods discretize the distribution into a finite number of bins [8,9,18,28,40], while others approximate the distribution using an analytical function such as a Gaussian [35,37,41]. Until now, the Gaussian approximation has only been used in two-state cross-bridge models without cooperative myofilament activation. It remains unclear whether a Gaussian approximation also yields sufficiently accurate simulations for more complex models. Since numerical efficiency is an important consideration in musculoskeletal simulations [42], a better understanding of how solution methods affect the processing time and accuracy of simulations is needed before cross-bridge models are implemented in movement simulation. Therefore, we explored the effect of approximating the cross-bridge distribution with a Gaussian on the ability to capture short-range stiffness across conditions.

**Our aim was to identify a muscle model suitable for musculoskeletal simulations of unsteady and perturbed movements based on its ability to capture muscles’ history-dependent resistance to stretch.** To this end, we evaluated muscle force predictions from various models against existing data on muscle forces for a broad range of behaviorally relevant movement histories and activation levels, recently documented in rat soleus muscle fibers [16]. We assessed the effects of including cooperative myofilament activation, a forcibly-detached cross-bridge state, and a Gaussian approximation of the cross-bridge distribution on the ability to capture short-range stiffness and its history dependence. Considering our aim to identify a model suitable for musculoskeletal simulations, we compared cross-bridge models to two Hill-type models commonly employed in musculoskeletal simulations. One of these Hill-type models included series compliance [42–47], while the other one did not [48–51]. These two types were included because some have interpreted Hill-type model series compliance as representing tendons [52–55] that are absent in the skinned fibers modeled here, while others have interpreted it as representing compliance within muscle fibers themselves [36,37]. We found that cross-bridge models with cooperative myofilament activation yielded better agreement with experimental data than both cross-bridge models without such dynamics, or Hill-type models. Approximating the cross-bridge distribution with a Gaussian or adding a forcibly-detached cross-bridge state had little effect on the force transients, but the former reduced processing time considerably.

## Results

After fitting to force trajectories during two stretch conditions at different calcium levels, cross-bridge models with cooperative myofilament dynamics captured forces across stretch-shortening-stretch trials better than Hill-type models and the two-state cross-bridge model, but only if cooperative myofilament dynamics were included. Neither a cross-bridge model lacking cooperative dynamics nor the Hill-type models captured the reduced muscle stiffness after a stretch-shorten cycle, i.e., muscle thixotropy. Adding cooperative myofilament dynamics reduced the rate of force development and the short-range stiffness after stretch-shorten cycles. Adding a forcibly-detached state slightly exaggerated the thixotropic behavior of the model. The cross-bridge models including cooperative dynamics robustly captured the effects of activation level, stretch-shorten amplitude, and recovery time on short-range stiffness. However, model forces still deviated from those observed experimentally in permeabilized, activated muscle fibers. Below, we first describe qualitative differences in force trajectories simulated based on different models. Then, we quantitatively evaluate how well different models capture force trajectories and muscle thixotropy across conditions.

**Fig 2:**
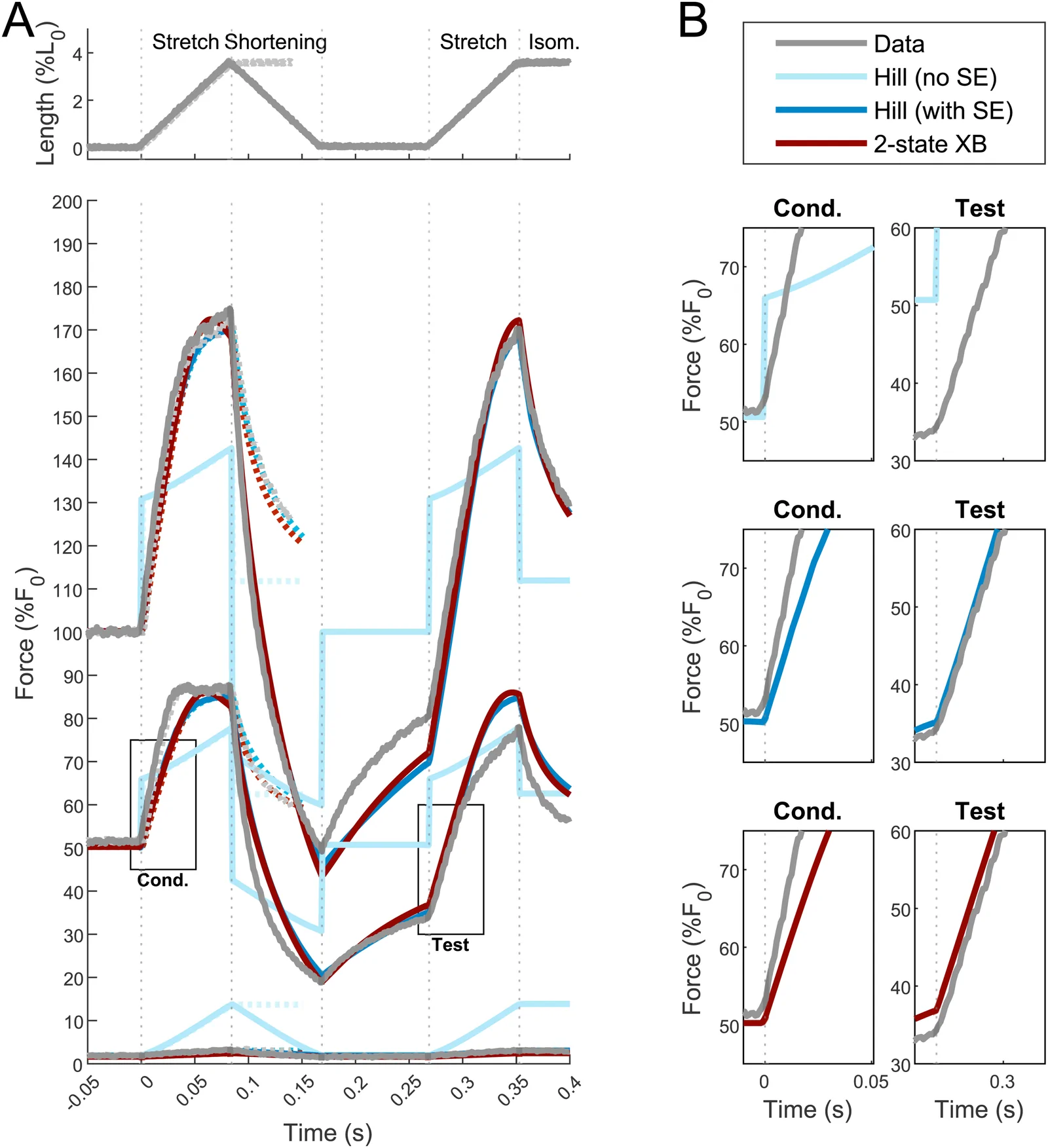
Modeled and measured forces for fitting trials: Hill-type models and a regular 2-state cross-bridge (XB) model. **A.** Entire protocol. Top row shows measured fiber length changes for a condition with a conditioning stretch (darker solid lines) and without a conditioning stretch (lighter dotted lines). Bottom row shows the corresponding empirical forces (“Data”, grey) at three different calcium concentrations (i.e. pCa 4.5, pCa 6.1 and pCa 9.0), alongside forces of a Hill-type model without a series element (“Hill (no SE)”, light blue), a Hill-type model with a series element (“Hill (with SE)", darker blue), and a regular 2-state cross-bridge model (“2-state XB”, red). **B.** Forces during the conditioning stretch (Cond.) and test stretch (Test) at submaximal activation levels.

**Cross-bridge dynamics alone were insufficient to explain muscle forces during stretch-shortening at submaximal activation levels.** A Hill-type model without series elasticity poorly captured the forces during the stretch-shortening-stretch protocol of the fitting trials (light-blue lines, Fig 2A). Adding series compliance to the Hill-type model considerably improved the agreement with experimental data (dark-blue lines, Fig 2A). The forces predicted by a 2-state cross-bridge (XB) model without cooperative dynamics (2-state XB model) were very similar to those predicted by the Hill-type model with series compliance (dark-red versus dark-blue lines, Fig 2A). For the fitting trial with conditioning stretch at the maximal activation level (solid lines, Fig 2A), both the Hill-type model and the 2-state XB model captured the rate of force development during stretch well. However, the simulated force trajectories differed considerably from the experimental force trajectories during the isometric period between stretches, where both models underestimated the rate of isometric force development after shortening. For the fitting trial with conditioning stretch at submaximal activation levels, both models failed to capture the rate of force development during stretch well. The Hill-type and 2-state XB models captured the rate of force development during the test stretch (“Test”, Fig 2A and 2B), but underestimated the rate of force development during the conditioning stretch (“Cond.”, Fig 2A and 2B). As a consequence, neither model captured the experimentally observed reduction in short-range stiffness during the test versus conditioning stretch. In addition, both models overestimated the force at the end of the test stretch. The Hill-type and 2-state XB models equally failed at capturing the history dependence of muscle short-range stiffness at submaximal activation levels.

**Fig 3:**
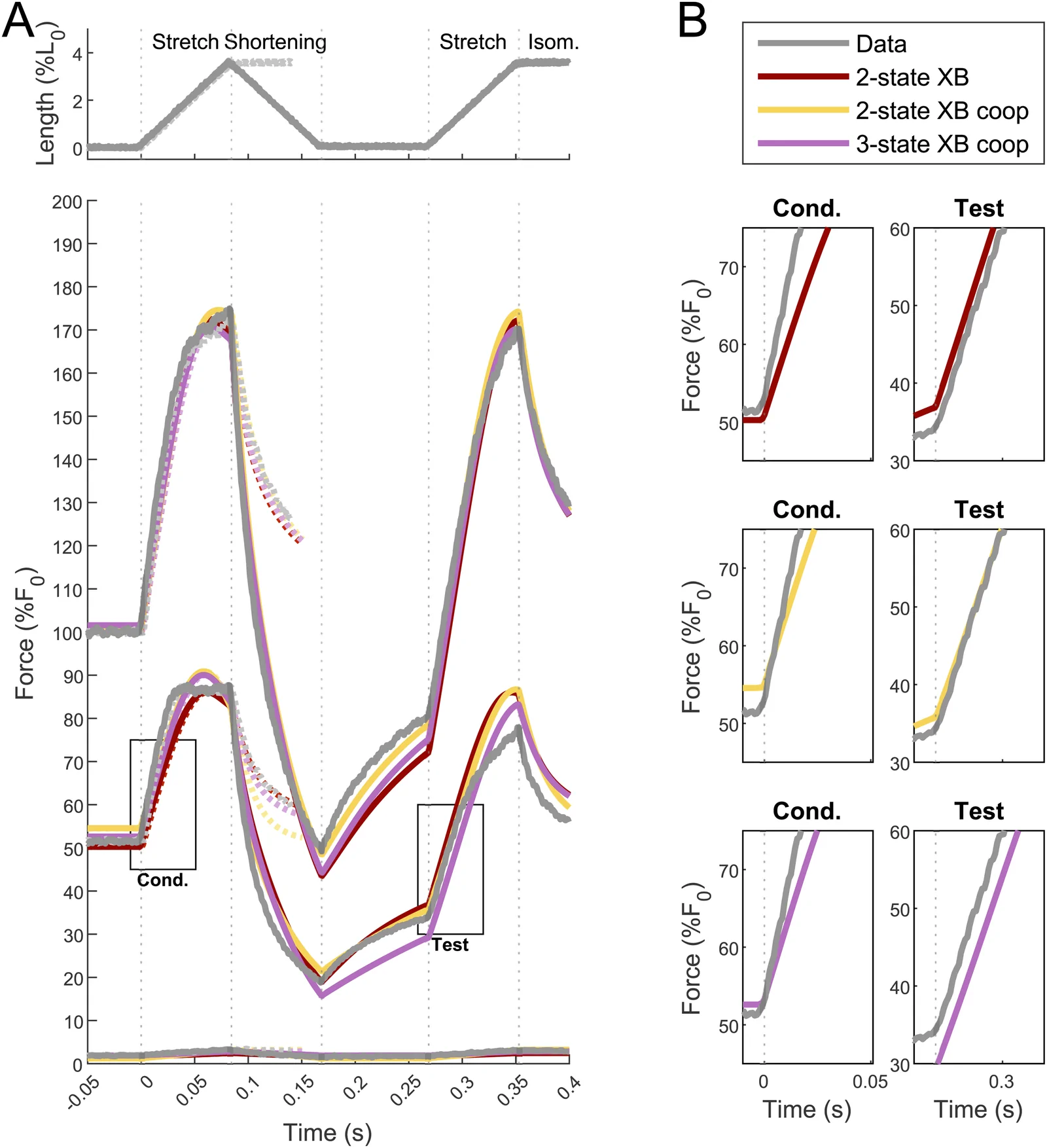
Modeled and measured forces for fitting trials: cross-bridge (XB) models. **A.** Entire protocol. Top row shows measured fiber length changes for a condition with a conditioning stretch (darker solid lines) and without a conditioning stretch (lighter dotted lines). Bottom row shows the corresponding empirical forces (“Data”, grey) at three different calcium concentrations (i.e. pCa 4.5, pCa 6.1 and pCa 9.0), alongside forces of a regular 2-state cross-bridge model (“2-state XB”, red), a cross-bridge model with cooperative thin filament activation (“2-state XB coop”, yellow), and a cross-bridge model with both cooperative thin filament activation and cooperative thick filament activation (“3-state XB coop”, purple). **B.** Forces during the conditioning stretch (Cond.) and test stretch (Test) at submaximal activation levels.

**Fig 4:**
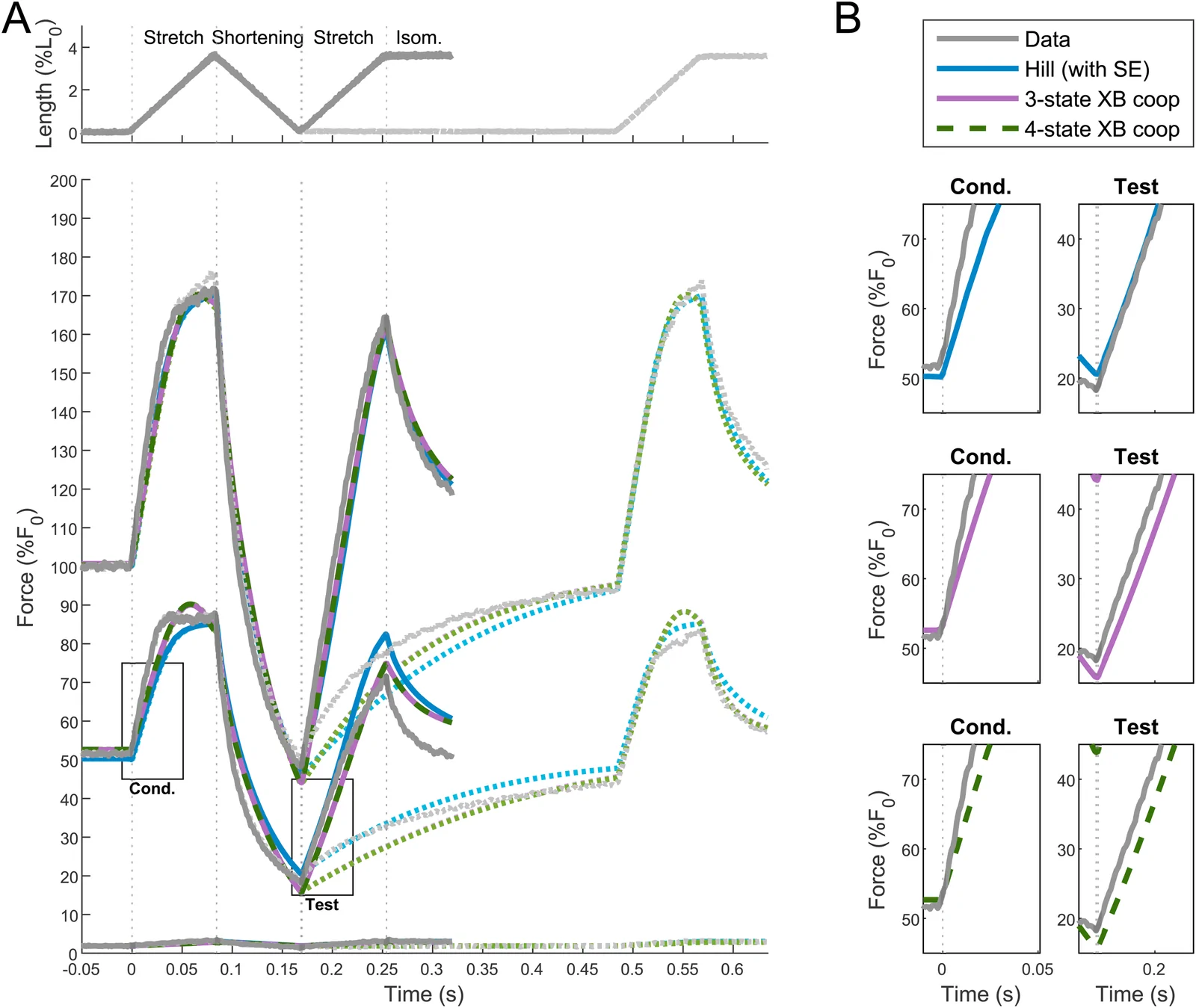
Modeled and measured forces for testing trials: cross-bridge (XB) models. **A.** Entire protocol. Top row shows measured fiber length changes for a condition with a short recovery of 0.001 s (darker solid lines) and with a longer recovery of 0.316 s (lighter dotted lines). Bottom row shows the corresponding empirical forces (“Data”, grey) at three different calcium concentrations (i.e. pCa 4.5, pCa 6.1 and pCa 9.0), alongside forces of a Hill-type model with a series element (“Hill (with SE)”, blue), a cross-bridge model with cooperative thin- and thick filament activation (“3-state XB coop”, purple), and a cross-bridge model with cooperative thin- and thick filament activation, and a forcibly-detached state (“4-state XB coop”, green). **B.** Forces during the conditioning stretch (Cond.) and test stretch (Test) at submaximal activation levels.

**Adding cooperative myofilament dynamics improved predictions of initial force rate upon stretch at submaximal activation levels.** For the fitting trial with conditioning stretch at maximal activation level, including thin (2-state XB coop) and thick (3-state XB coop) filament dynamics had little effect on force rate during stretch but increased the isometric rate of force development after shortening, yielding better agreement with experimental data (yellow and purple lines versus dark red line, Fig 3A). For the fitting trial with conditioning stretch at submaximal activation levels, including thin and thick filament dynamics increased the force rate during the conditioning stretch and yielded better agreement between simulated and experimental reductions in short-range stiffness of the test versus conditioning stretch. However, the 3-state XB coop model’s isometric rate of force development at submaximal activation levels was too slow compared with empirical data and the models with thin and thick filament dynamics also overestimated the force at the end of the test stretch, although the 3-state model did less so than the other models. The 3-state XB coop model captured the decay in force during the isometric phase of a trial without conditioning stretch better than the 2-state XB coop model (dotted lines, Fig 3). Similar results were obtained for testing trials with different recovery times (Fig 4; 3-state XB coop but not 2-state XB coop model shown). Note that the testing trial with a longer recovery time (RT = 0.316 s, dotted lines Fig 4) at submaximal activation levels reveals that the initial underestimation of isometric force recovery of the 3-state XB model is offset by a subsequent overestimation of the isometric force recovery. Together, this visual analysis shows that history-dependent short-range stiffness reductions at submaximal activation levels can be reproduced by modeling cooperative myofilament activation.

For this typical example fiber, there was little to no difference between the forces of the 3-state versus 4-state XB coop models (purple versus green lines, Fig 4), suggesting that adding a forcibly-detached cross-bridge state has a small effect on force output. The 3-state and 4-state XB coop models better captured the history dependence of muscle short-range stiffness at submaximal activation levels compared with the Hill-type models, the 2-state XB model and the 2-state XB coop model. Thus, for the models tested here, both thin filament and thick filament cooperative activation are needed to capture the history dependence of muscle short-range stiffness.

**Fig 5:**
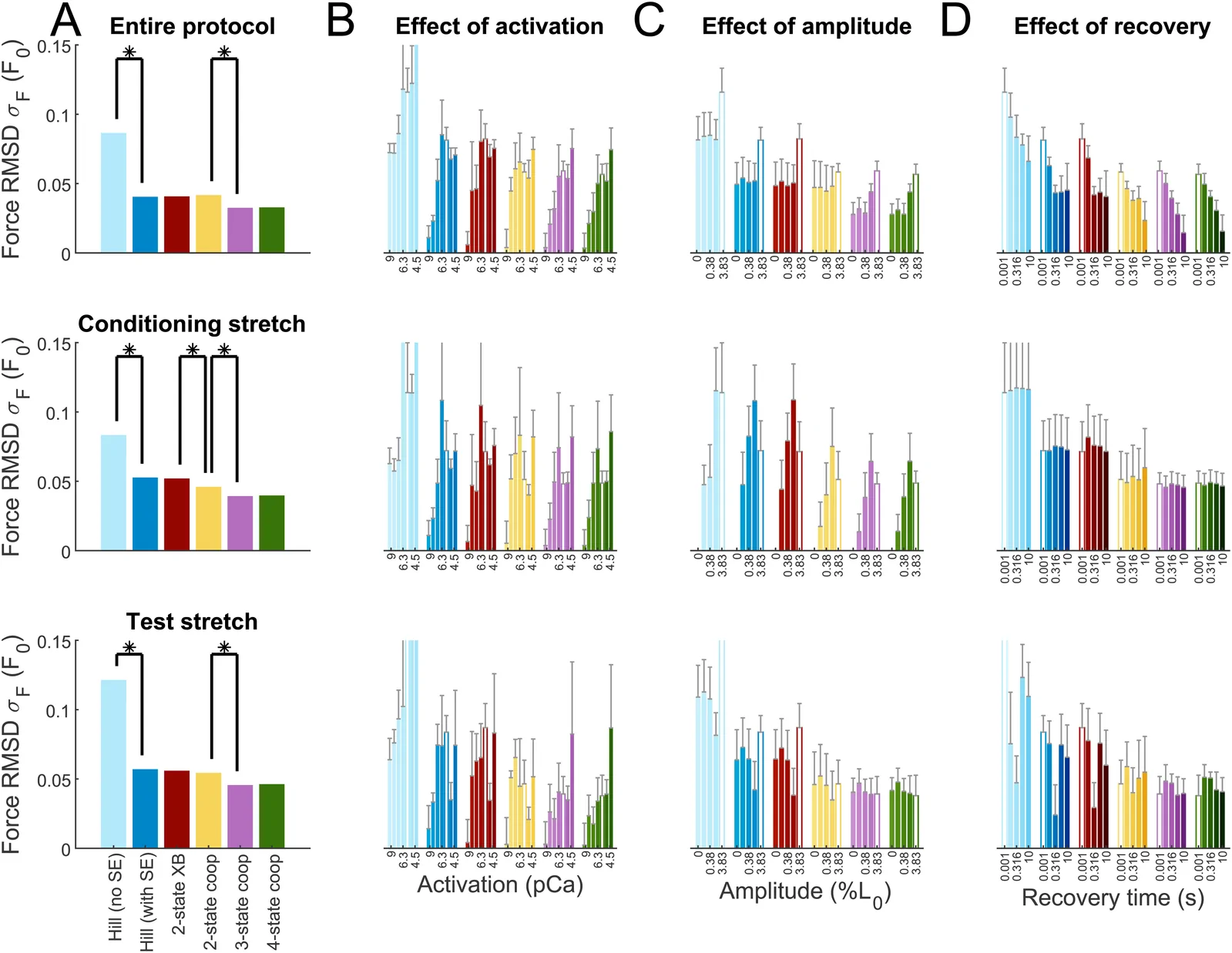
Root-mean-squared deviations (RMSDs) between model and experimental forces, normalized with respect to the maximal isometric force F_0_. Force RMSDs $\sigma_{F}$ are shown for Hill-type models without and with series elasticity (“Hill”, light and dark blue), 2-state cross-bridge model (“2-state XB”, red), 2-state cross-bridge model with cooperative dynamics (“2-state coop”, yellow), 3-state cross-bridge model with cooperative dynamics (“3-state coop”, purple) and 4-state cross-bridge model with cooperative dynamics (“4-state coop”, green). RMSDs are shown for entire stretch-shorten protocol (top row), conditioning stretch (middle row) and test stretch (bottom row). **A.** Averaged over all trials. Asterisks indicate significance at p < 10^-4^. **B.** Effect of calcium activation level at constant amplitude (3.83%L_0_) and recovery time (0.001 s). **C.** Effect of stretch amplitude at constant activation level (pCa 6.3) and recovery time (0.001 s). **D.** Effect of recovery time at constant amplitude (3.83%L_0_) and activation level (pCa 6.3). In panels B, C and D, darker shades indicate lower pCa, larger amplitude, and longer recovery, respectively. In these panels, bars with a white surface area indicate the trial that is common to all three sets of conditions.

**Modeling cooperative myofilament dynamics improved the accuracy of simulated force trajectories during stretch-shorten-stretch cycles, whereas additionally modeling a forcibly-detached state has little effect on the overall agreement between model and experimental forces.** In line with exemplar force trajectories (Figs 2–4), fiber-average results indicated that predictions from the 3-state XB coop model and 4-state XB coop model agreed better with data than predictions from the 2-state XB model, 2-state XB coop model and the Hill-type models (Fig 5). The root-mean-square deviation between model forces and empirical forces $\sigma_{F}$ generally decreased with model complexity (see Fig 5A and Table 1). Comparing models with increasing complexity, differences in overall $\sigma_{F}$ were significant (at p < 10^-4^) between the Hill-type models with and without series compliance and between the 2-state XB coop and 3-state XB coop models. Thus, adding both series compliance and thick filament dynamics was important to improving model fits. For most models, $\sigma_{F}$ increased with higher calcium activation levels (i.e., lower pCa, Fig 5B), larger amplitude of the conditioning stretch (Fig 5C) and shorter recovery time (Fig 5D). The increase in $\sigma_{F}$ with activation level and amplitude is not that surprising, as conditions with larger activation and amplitude are associated with larger (passive) forces. Across calcium activation levels, stretch amplitudes and recovery times (Fig 5B–5D), the 3-state and 4-state XB coop models generally yielded lowest $\sigma_{F}$. Thus, force predictions of the 3-state and 4-state XB coop models generally matched experimental forces better than force predictions from the Hill-type and 2-state XB models.

**Table 1:** Model accuracy.

Model	Force RMSD $\sigma_{F}$	SRS RMSD $\sigma_{SRS}$	Relative AIC score
Hill-type (no SEE)	0.121	0.140	0
Hill-type (with SEE)	0.057	0.204	67.4
2-state XB	0.056	0.141	10.6
2-state XB coop	0.054	0.121	-14.7
3-state XB coop	0.046	0.104	-37.3
4-state XB coop	0.046	0.103	-37.5

**Fig 6:**
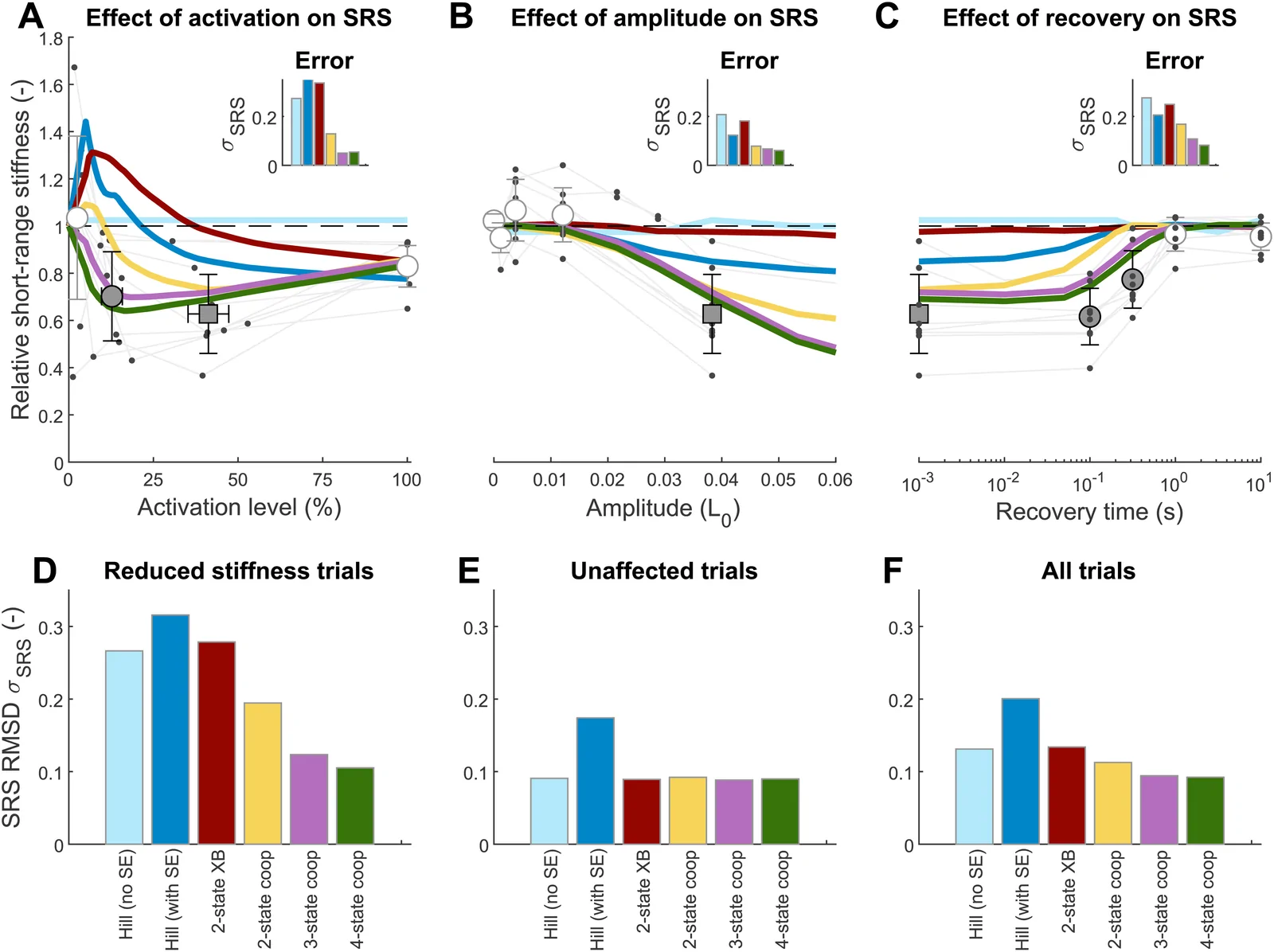
Empirical and model relative short-range stiffness across muscle activation levels, stretch amplitudes and recovery times. Fiber-averaged (n = 7) model predictions and errors are shown for Hill-type model without series compliance (“Hill (no SE)”, light blue), the Hill-type model with series compliance (“Hill (with SE)”, dark blue), the 2-state cross-bridge model (“2-state XB”, red), 2-state cross-bridge model with cooperative dynamics (“2-state coop”, yellow), 3-state cross-bridge model with cooperative dynamics (“3-state coop”, purple), and 4-state cross-bridge model with cooperative dynamics (“4-state coop”, green). Data are shown both averaged across all fibers (n = 11, grey circles), and for individual fibers (black dots). Four (out of 11) fibers were not modeled because they did not include stretch-shortening-stretch trials at Intermediate and/or Maximal activation levels, which was key to parameter fitting. Filled symbols and error bars indicate mean ± s.d. across trials with considerable short-range stiffness reductions, open symbols indicate trials with little to no short-range stiffness reductions. Squares indicate the condition that panels A-C have in common. **A.** Effect of activation level at constant amplitude (3.83%L_0_) and recovery time (0.001 s). **B.** Effect of amplitude at constant activation level (Intermediate) and recovery time (0.001 s). **C.** Effect of recovery time at constant activation level (Intermediate) and amplitude (3.83%L_0_). **D.** Model SRS error, quantified as SRS RMSD $\sigma_{SRS}$, averaged over trials with large short-range stiffness reductions. **E.** $\sigma_{SRS}$ averaged over trials with small short-range stiffness reductions. **F.** $\sigma_{SRS}$ averaged over all trials.

**Modeling cooperative myofilament activation was important to accurately capture short-range stiffness changes across conditions.** The 3-state and 4-state XB coop models captured the effect of muscle activation level (Fig 6A), conditioning stretch amplitude (Fig 6B), and recovery time (Fig 6C) on relative short-range stiffness ($\sigma_{SRS}$≤ 0.1), whereas the 2-state XB coop model only captured some effects. In contrast, the Hill-type models and 2-state XB model did not capture any of these effects ($\sigma_{SRS}$ > 0.15, Fig 6A–6C). The Hill-type model without series compliance incorrectly predicted that relative short-range stiffness was constant across activation levels, amplitudes, and recovery times (light blue line, Fig 6A–6C). Both the 2-state XB model and the Hill-type model with series compliance incorrectly predicted a continued decline in relative short-range stiffness instead of the experimental U-shaped relation between relative short-range stiffness and muscle activation level (red and dark-blue lines, Fig 6A). The Hill-type model with series compliance correctly predicted that relative short-range stiffness decreases with larger amplitude (Fig 6B) and shorter recovery (Fig 6C) but underestimated the magnitude of these effects. The 2-state XB model did not predict a decrease in short-range stiffness with greater amplitude or shorter recovery. Thus, incorporating both thin and thick filament cooperative dynamics improves the ability to capture short-range stiffness across a broad range of conditions.

**Fig 7:**
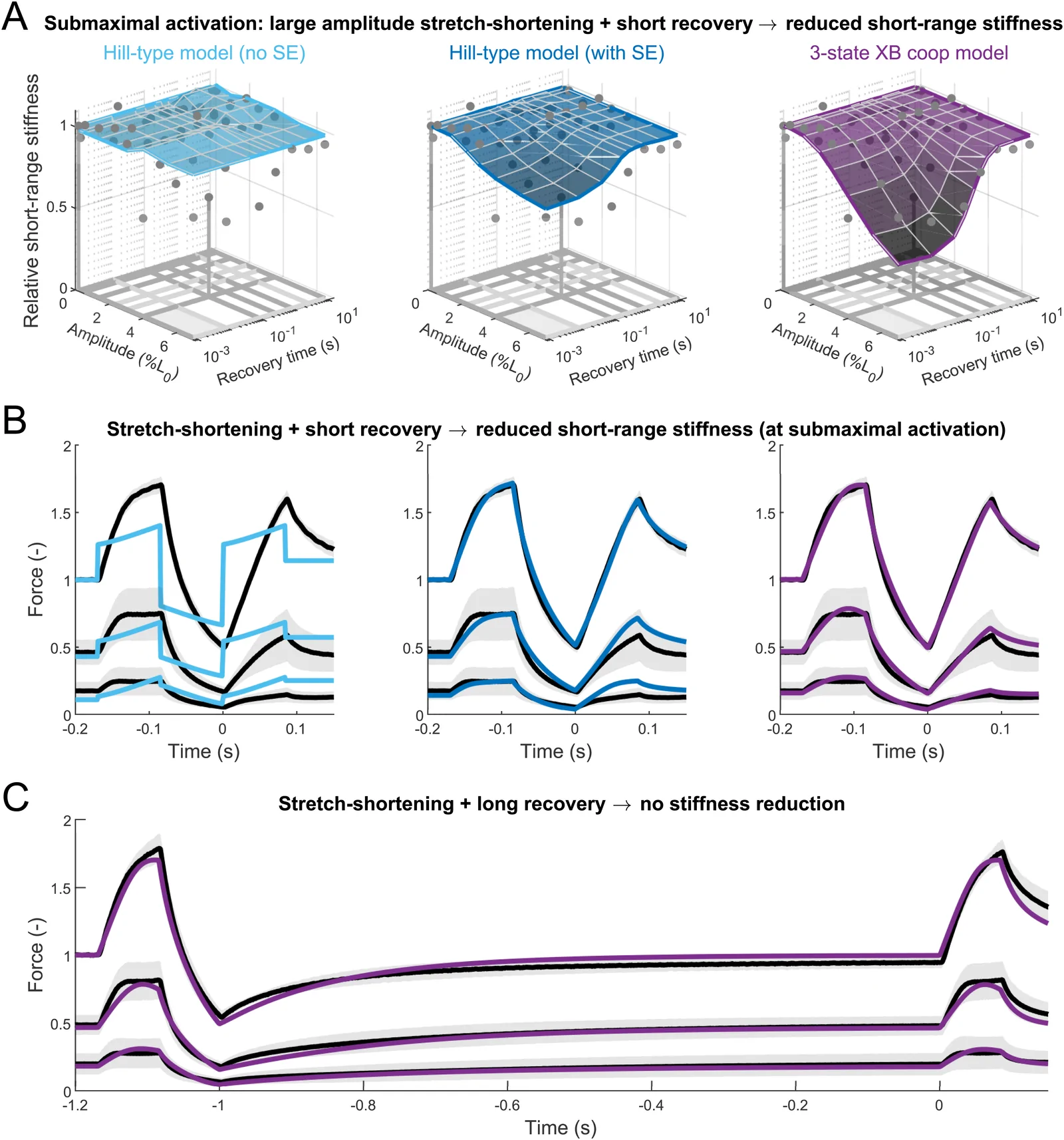
Empirical and model relative short-range stiffness across conditions. Model predictions are averaged over all fibers in the fitting set (n = 7), data are averaged over all data in the dataset (n = 11). **A.** Relative short-range stiffness at submaximal activation levels across amplitudes and recovery times. Relative short-range stiffness is indicated by both the vertical location and surface color. Fitting trials are indicated with stems, connecting to the corresponding dots. The bottom surface indicates the experimental grid of stretch amplitudes and recovery times, distinguishing between conditions measured in all fibers (dark grey lines, n = 11) and conditions measured in a subset of fibers (light grey lines, n = 3), conditions with history-dependent stiffness reductions (grey surface) and conditions without such stiffness reductions (white surface). At submaximal activation levels, empirical short-range stiffness (grey dots) decreases with larger stretch amplitudes and shorter recovery times. This is not captured by the Hill-type model without series compliance (light blue, left), partially captured by the Hill-type model with series compliance (dark blue, middle), and mostly captured by the 3-state XB coop model (purple, right). **B.** Fiber-average time-series of force trajectories during stretch-shorten-stretch with a short recovery time at 3 different activation levels for data (black), Hill-type model without series compliance (light blue, left), Hill-type model with series compliance (dark blue, middle), and 3-state XB coop model (purple, right). Shaded area indicates mean ± 1 standard deviation. **C.** Fiber-average time-series of force trajectories during stretch-shorten-stretch with a long recovery time at 3 different activation levels for data (black), and 3-state XB coop model (purple).

**A cross-bridge muscle model with cooperative myofilament activation predicts muscle thixotropy across behaviorally relevant conditions.** Averaged over trials with large short-range stiffness reductions (Fig 6D), $\sigma_{SRS}$ were lowest for the 4-state XB coop model ($\sigma_{SRS}$ = 0.101) and highest for the Hill-type model with series compliance ($\sigma_{SRS}$ = 0.312). Averaged over trials with small short-range stiffness reductions (Fig 6E), $\sigma_{SRS}$ were similar across models ($\sigma_{SRS}$ range: 0.103-0.180). Averaged over all 84 trials in the dataset (Fig 6F and Table 1), $\sigma_{SRS}$ was lowest for the 3-state and 4-state XB coop model ($\sigma_{SRS}$ = 0.103-0.104) and highest for the Hill-type model with series compliance ($\sigma_{SRS}$ = 0.204). Short-range stiffness predictions of the 3-state and 4-state XB coop models yielded the lowest AIC scores (Table 1), suggesting that these models provide the best trade-off between complexity and accuracy. While the Hill-type models did not capture short-range stiffness changes across conditions, the 3-state XB coop model reproduced the observation that relative short-range stiffness decreases with combinations of shorter recovery times and higher conditioning stretch amplitudes (Fig 7A) characteristic of abnormal postural sway. Unlike the Hill-type models, the 3-state XB coop model predicted a considerable reduction (i.e., > 30%) of muscle short-range stiffness at combinations of conditioning amplitudes greater than 3% and recovery times lower than 0.5 s, characteristic of normal postural sway (Fig 7B). It also predicted that short-range stiffness would not be affected by stretch-shortening if recovery times were sufficiently long (Fig 7C). Altogether, the 3-state and 4-state XB coop models yield greatest accuracy (based on ΔAIC) across all conditions in the dataset.

**Fig 8:**
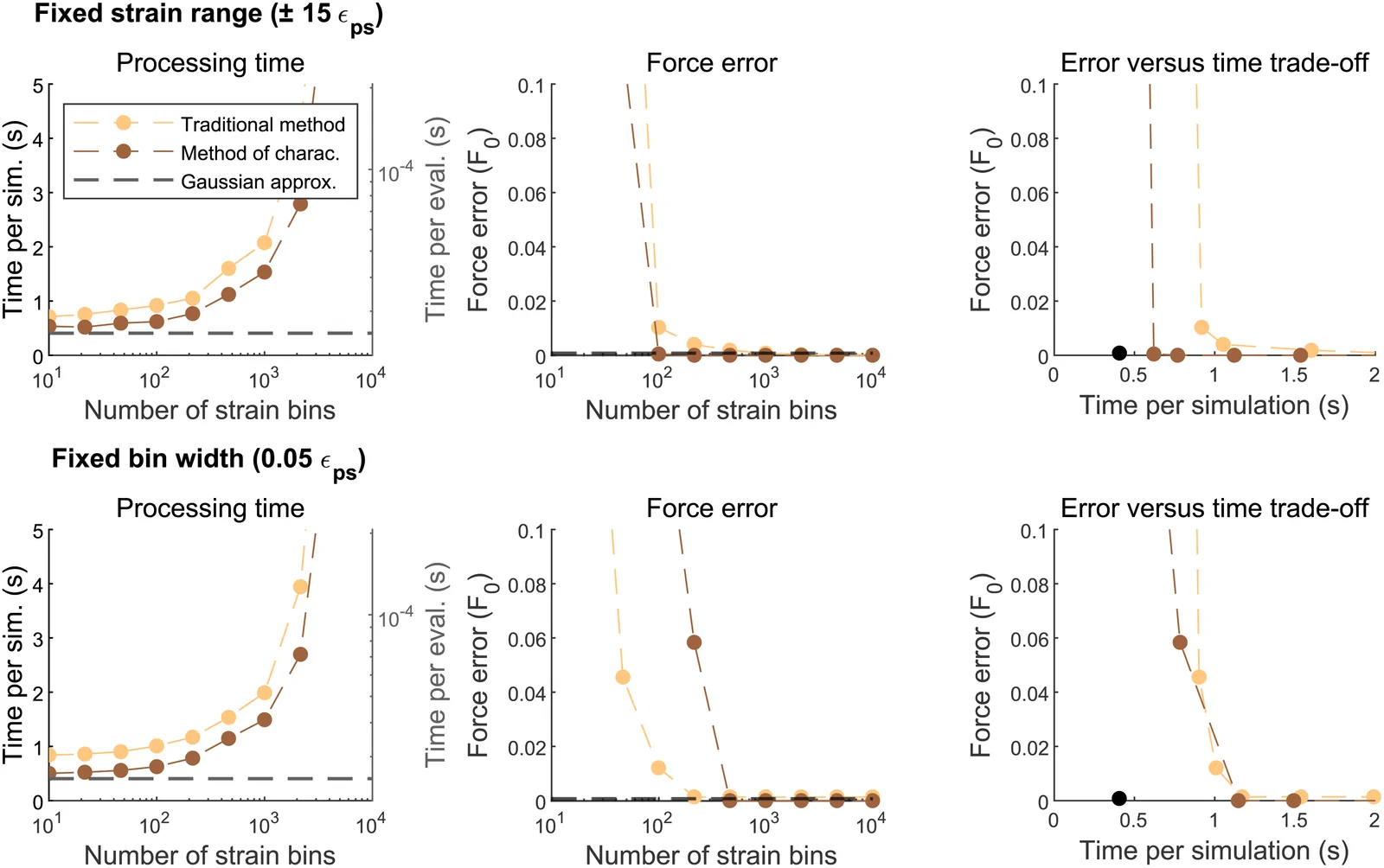
Computational cost of discretized and approximated cross-bridge models. The processing time (left column) and the force error (middle column) of solution methods based on discretization depends on the strain vector used. The strain vector can be varied in two ways: (1) fixing the strain range and increasing the number of bins (top row), or (2) fixing the bin width and increasing the number of bins (bottom row). In both cases, processing time and force error increase and decrease with the number of strain bins. Consequently, the force error depends on the processing time (right column). Two types of discretization methods are shown: the traditional method (yellow) and the method of characteristics (brown). In addition, a Gaussian approximated solution method is shown (black dotted line and black dot).

**Approximating the cross-bridge distribution with a Gaussian had a small effect on predicted force trajectories during stretch-shorten-stretch cycles but reduced the computational cost by an order of magnitude**. Approximating the cross-bridge distribution by a Gaussian had a small effect on force trajectories (Fig 2 versus S1 Fig, Fig 3 versus S2 Fig and Fig 4 versus S3 Fig), force RMSDs (Fig 5 versus S4 Fig) and short-range stiffness predictions (Fig 6 versus S5 Fig) across cross-bridge models. We performed a detailed analysis of processing time and accuracy for the 3-state XB coop model (Fig.8). Computational cost and accuracy of discretization methods was influenced by choices regarding the discretization of the strain. For the discretization methods (yellow and brown dots, Fig 8), a large number of strain bins resulted in a higher processing time (left column, Fig 8) and a smaller force error (middle column, Fig 8). The error versus time trade-off at a given strain bin was somewhat better for the method of characteristics (brown dots) than for the traditional method (Fig 8, right column, brown curve below yellow curve meaning a lower error for same processing time), but the traditional method required fewer constant width strain bins for the force error to drop below 0.1% F_0_ compared with the method of characteristics (Fig 8, bottom row middle column). When using the approximated solution method (black dashed line and black dot, Fig 8), force error was < 0.01% F_0_, while a single function evaluation only required about 0.000017 s. This yielded a simulation time of 0.4 s for simulating a trial with a duration of 2.4 s and using a fixed time step of 0.0001 s. Using the discretization methods with strain vectors that yielded a similar force error (method of characteristics, 100 strain bins, strain range: ± 15 $\epsilon_{ps}$; brown dots, Fig 8), a single function evaluation required about 50% more time (0.000025 s) and simulating the 2.4-s trial using a fixed time step of 0.0001 s required 0.6 s. When using a variable time-step integrator, the approximated model required 7.1 times fewer function evaluations than the methods of characteristics with similar accuracy (force error of 0.05%F_0_ versus 0.08%F_0_), resulting in an 8.6-times shorter processing time. Most of the computational cost difference was thus due to a reduction in the number of function evaluations due to the larger time steps enabled by a reduction in the stiffness of the state derivative equations. Altogether, approximating the cross-bridge distribution by a Gaussian has little influence on the simulated force trajectory but improves computational efficiency.

## Discussion

Here we present a cross-bridge model capturing skeletal muscle’s history-dependent reduction in short-range stiffness, referred to as muscle thixotropy. As anticipated, commonly-used phenomenological Hill-type models did not predict short-range stiffness reductions at submaximal activation levels. However, to our surprise, modeling actin-myosin interactions did not improve predictions of muscle thixotropy compared to the Hill-type model with series elasticity. Instead, adding cooperative dynamics for myofilament activation was key to capturing history-dependent short-range stiffness reductions across behaviorally relevant conditions. Adding a forcibly-detached myosin state had a small effect on short-range stiffness predictions. Cross-bridge distributions could be accurately approximated by a Gaussian, considerably reducing the processing time of simulating force trajectories and thereby facilitating the practical use of cross-bridge muscle models in musculoskeletal simulation. We recommend that musculoskeletal simulations of movements that induce history-dependent short-range stiffness changes use Gaussian-approximated cross-bridge models with cooperative myofilament dynamics.

**State-of-the-art musculoskeletal simulations of whole-body movements employ Hill-type models that lack short-range stiffness or history-dependent properties.** Here, we tested short-range stiffness predictions of two different Hill-type models commonly employed in movement simulations: one with series compliance [42–47] and one without series compliance [48–51]. We found that the Hill-type model with series compliance greatly outperformed the Hill-type model without series compliance for predicting fiber force trajectories and relative short-range stiffness across conditions (e.g., Figs 5–7). The history dependence of the Hill-type model with series compliance arose from the exponential force-length relation of the series elastic element, resulting in greater compliance at smaller forces. As shortening results in smaller forces, forces at the onset of the test stretch were smaller than those at the onset of the conditioning stretch. Hence, there might be considerable series compliance in muscle fibers. Although more and more movement simulations include series compliance in Hill-type models [42–47], it is neglected in static optimization - a popular method for estimating muscle forces during movement [48–50]. Series compliance is also neglected in forward simulation frameworks that aim at maximizing computational efficiency, such as MuJoCO [51]. While non-linear series compliance might explain why short-range stiffness is reduced after shortening, it does not account for the non-linear effect of activation level on stiffness reductions. Consequently, the Hill-type model erroneously predicted that the greatest short-range stiffness reductions occur at maximal activation level, while data indicate that they occur at submaximal activation levels (Fig 6A). Thus, while series compliance may contribute to short-range stiffness and its history dependence, it does not explain why short-range stiffness reductions are greatest at submaximal activation levels. While more complex Hill-type models than those tested here have been developed [e.g., 13,14,56–62], it is unlikely that any of these models would have captured short-range stiffness across the conditions tested here. Hill-type model extensions that aimed at better capturing short-range stiffness greatly simplified the influence of movement history, distinguishing only between the presence or absence of prior movement [13,14]. Other extensions aimed at capturing other phenomena not described by simple Hill-type models, such as length-dependent activation [56,57], non-linear calcium dynamics [59,62], transverse loading [60], and serial damping [58]. Yet, forces of these more complex Hill-type models remain uniquely determined by muscle activation, length and velocity. These more complex models therefore cannot account for observed differences in short-range stiffness between two stretches at identical activation, length and velocity (see Figs 1–4). Altogether, our results indicate that Hill-type models commonly employed in movement simulations do not capture the history dependence of short-range stiffness, and underscore the importance of including series compliance in muscle models.

**In contrast to simple Hill-type models and a simple two-state cross-bridge model, a muscle model with cooperative thin and thick filament dynamics predicted that muscle thixotropy is greatest at submaximal activation levels.** While neither Hill-type models nor the cross-bridge model without cooperative myofilament dynamics tested here predicted the U-shaped relation between muscle activation and relative short-range stiffness, all models with cooperative myofilament dynamics did (Fig 6A). In the 2-state XB coop model, all cooperativity is attributed to the thin filament. This seems to be reflected in the thin filament cooperativity parameter k_c_, which was ~ 4 times higher in this model than in the 3-state and 4-state XB coop models (see Table 3). The higher degree of thin filament cooperativity in the 2-state XB coop model could not fully compensate for the absence of thick filament cooperativity, as both the 3-state and 4-state XB coop model better captured the relative short-range stiffness reductions at the lowest activation levels (Fig 6A), and also yielded the best predictions of the effects of stretch amplitude (Fig 6B) and recovery time (Fig 6C). The 3-state and 4-state XB coop model yielded similar force and short-range stiffness predictions (Figs 4–6). Furthermore, the 3-state XB coop model reproduced short-range stiffness changes across a broad range of conditions (Fig 7). It may thus be useful to employ cross-bridge models that include cooperative dynamics for thin- and thick filament activation for predicting short-range stiffness during movement.

**A Gaussian approximation provides a good trade-off between accuracy and computational demand.** Force traces simulated based on a discretization of the cross-bridge distribution - which does not require any assumptions about its shape - were similar to force traces simulated under the assumption of a Gaussian distribution (Fig 2 versus S1 Fig, Fig 3 versus S2 Fig and Fig 4 versus S3 Fig). Similarly, errors in the prediction of force and short-range stiffness were similar when using discretization or a Gaussian approximation to simulate cross-bridge dynamics (Fig 5 versus S4 Fig and Fig 6 versus S5 Fig). Unlike other simplified solution methods such as those based on velocity-dependent distortion [63–65], the Gaussian-approximated solution method resolves Huxley’s partial differential equation describing cross-bridge dynamics (Eq. 1). Consequently, for conditions in which a Gaussian adequately represents the shape of the actual distribution, simulations with the Gaussian-approximated solution method will mimic those with solution methods based on discretization. Despite similarities in simulated forces and short-range stiffness, computational cost was ~ 8 times smaller for the approximated model. This reduction in computational cost was largely due to a reduced number of integration steps needed, which suggests that the dynamics of the discretized model were stiffer than those of the approximated model. Zahalak [35] was the first to approximate the cross-bridge distribution with a Gaussian, arguing that although the actual cross-bridge distribution may not be Gaussian, a Gaussian function may adequately reflect the distribution’s first three moments (i.e., ensemble cross-bridge stiffness, force, and elastic strain energy). While Zahalak [35] used Huxley’s [34] piece-wise linear cross-bridge attachment function that was proposed for mathematical convenience, more recent cross-bridge models [e.g., [20,31]] use a Gaussian attachment function that better captures the underlying mechanisms (i.e., random Brownian motion of myosin heads). Such a Gaussian attachment function results in a Gaussian-like cross-bridge distribution during isometric muscle contraction. In addition, while Zahalak used piece-wise linear detachment functions, more recent models [31] use more mechanistically accurate exponential cross-bridge detachment functions that help preserve a Gaussian-shaped distribution during length changes. Thus, a Gaussian may be an even better approximation of the cross-bridge distribution than originally conceived. We therefore recommend to use Gaussian-approximated cross-bridge models for predicting short-range stiffness during movement.

**Models explain how cooperative myofilament dynamics may cause an activation-dependent reduction in cross-bridge cycling rates after shortening, which may underlie the activation-dependent movement history-dependence of short-range stiffness.** It has been recognized that short-range stiffness reductions after shortening are more pronounced at submaximal activation levels than at maximal activation levels [9], but the underlying mechanisms have remained unclear to date [10]. Our computational results provide insight into how cooperative thin filament activation and cooperative thick filament activation may contribute to the activation dependence of short-range stiffness reduction. In the cooperative cross-bridge models considered here, both thin and thick filament activation are inherently coupled to cross-bridge cycling. The XB coop models predict that the effect of cross-bridge attachment on thin filament activation is greater when [Ca^2+^] alone does not suffice to fully activate the thin filament. When muscle shortens at these submaximal activation levels, cross-bridge detachment reduces thin filament activation, which reduces subsequent cross-bridge attachment and thereby reduces short-range stiffness. Because it takes time to reactivate the thin filament, this stiffness reduction persists for some time after shortening. The U-shaped relation appears because these cooperative effects are smaller at both maximal activation – where [Ca^2+^] alone fully activates the thin filament – and in passive conditions where no cross-bridges attach. Cooperative thick filament activation augments these effects through its force dependence. The 3-state XB coop model predicts that the shortening-induced force reduction due to sliding filaments (Fig 1) causes force-dependent myosin heads to revert to the super-relaxed state. This would result in a greater number of super-relaxed myosin heads after shortening, further slowing subsequent cross-bridge re-attachment and stiffness recovery. In the 3-state XB coop model, the rate at which myosin heads switch back to the disordered-relaxed state is force dependent. Because forces are generally smaller at submaximal activation, thick filament dynamics (and hence short-range stiffness recovery) is slower. In line with this model-based explanation, the inclusion of cooperative thin filament activation yielded the U-shaped relation (2-state XB coop model, Fig 6), while the inclusion of cooperative thick filament activation further deepened the U-shape at low activation (3-state XB coop model, Fig 6). It is important to note that these model-based explanations warrant experimental verification. As models inherently simplify reality, it is probable that other mechanisms – not modeled here – also contribute to muscle thixotropy. Yet, these computational results suggest that both thin and thick filament activation contribute to the history dependence of short-range stiffness.

**Adding a forcibly-detached cross-bridge state has a small effect on short-range stiffness predictions**. While cross-bridge models with cooperative myofilament dynamics captured thixotropy well, model force trajectories still deviated from experimental data (Figs 2–6). To our surprise, the model with a forcibly-detached state did not yield improved predictions of the plateau phase of the force response. This could be because the stretches considered here were relatively slow (0.45 L_0_/s) and brief (< 0.1 s). Indeed, when evaluating model predictions for faster and longer stretches, differences between models with and without a forcibly-detached state were larger (S7 Fig). In response to faster stretches, forces of the 4-state XB coop model had smaller overshoot and undershoot (sometimes referred to as “yield” [35,36] or “give” [66,67]) than those of the 3-state XB coop model. Yet, for the dataset considered here, the additional complexity and number of parameters of the 4-state XB coop model did not justify the inclusion of a forcibly-detached state to describe history-dependent short-range stiffness (based on AIC). A weakly-bound cross-bridge state with fast dynamics has been suggested as an alternative mechanism to explain the force plateau during stretch. Such a weakly-bound state has been included in cross-bridge models [8–10,24,68]. While we focused on short-range stiffness here, future studies could explore which model dynamics are required for more accurately capturing the force plateau during stretch and thus for increasing the overall agreement with experimental force trajectories.

**In addition to underlying history-dependent short-range stiffness changes, cooperative myofilament dynamics may also contribute to other aspects of muscle mechanics.** A limitation of the current study is that we did not explicitly model the rightward shift of the force-length relation with lower activation levels [56,57,69–72], here referred to as length-dependent activation. This phenomenon has recently been attributed to interactions between filament overlap and interfilament spacing [73]. In addition, previous modeling studies have indicated that cooperative myofilament dynamics contributes to length-dependent activation [20,71,72,74]. Thus, interfilament spacing and cooperative myofilament dynamics may be complementary mechanisms underlying muscle force production. Previous modeling studies also suggested that cooperative myofilament dynamics may contribute to muscle’s activation-dependent rate of force development [25,28,41,75], the reduction in muscle force after shortening [31] (i.e., force depression), and the increase in isometric muscle force after stretch (i.e., residual force enhancement). The latter phenomenon is thought to be mediated by titin [31,76–78] and half-sarcomere inhomogeneity [79,80]. One theory is that cross-bridge cycling strains titin and thereby increases force produced by titin [77]. If this is the case, cooperative myofilament dynamics will also influence the rate at which titin is strained and thus residual force enhancement. It may also be interesting to consider the effect of fiber type on these phenomena. While fibers in our dataset were predominantly slow-twitch, muscle thixotropy has also been observed in fast-twitch fibers. Fast-twitch fibers are typically associated with faster cross-bridge cycling rates, and faster short-range stiffness recovery times [8,9]. In fast-twitch fibers, more cross-bridges are thought to be attached at any given time, potentially explaining why these fibers have both larger forces and higher metabolic rates when normalized for cross-sectional area [81]. Future modeling studies could explore the extent to which cooperative myofilament dynamics can simultaneously explain multiple aspects of muscle mechanics and energetics across different fiber types.

**While the cross-bridge models considered are more mechanistic than Hill-type models, they still entail hugely simplified descriptions of the underlying biophysical processes.** Some aspects of the cross-bridge models considered here are rooted in our understanding of the biophysics of muscle contraction. For example, the increased cross-bridge detachment rate at lower cross-bridge strains reflects the force dependence of ADP release, the rate-limiting step in cross-bridge detachment [31,82,83]. But other aspects of the cross-bridge models are still somewhat simplistic and descriptive. For example, our models do not account for changes in force or calcium sensitivity due to interfilament spacing [73], which will be key to capturing forces across a broader range of length changes than the short-range stretches modeled here. Furthermore, our models assume that cross-bridges behave as linear springs, while muscle’s mechanical response to stretch has a viscous component [67]. Some of this simplicity arises from the fact that our understanding of the biophysics of muscle contraction remains incomplete. While evidence for the force dependence of myosin’s super-relaxed state is accumulating, the underlying mechanisms remain unclear. Titin may play a role, considering that it can transfer force to myosin and thereby induce structural alterations to the thick filament [74,84]. Titin may also be important for capturing the velocity-dependent force response to longer stretches than those considered here [67,85–88], rheopexy observed during slower stretches [32], and the increase in stiffness over multiple stretch-shortening cycles [89]. The lack of titin dynamics in the models considered here may in part explain why we still observed deviations between model force trajectories and empirical force trajectories, despite finding relatively good agreement during the short-range stiffness phase. Alternatively, errors could arise from modeling thin and thick filament activation globally instead of locally [31,90–92], from neglecting (half-)sarcomere inhomogeneity [79,80], or neglecting changes in interfilament spacing [73]. As far as we know, no existing model has simultaneously explained all aspects of muscle history dependence, including muscle thixotropy, residual force depression, and residual force enhancement. Considering that various aspects of muscle mechanics may arise from interactions between actomyosin and titin dynamics within and between (half-)sarcomeres, future modeling studies should integrate these dynamics to generate a comprehensive understanding of muscle contraction.

**Modeling muscle thixotropy and short-range stiffness may be crucial to simulate musculoskeletal movements where muscles act eccentrically.** Muscle thixotropy has been observed across a wide range of scales, ranging from single fiber to human movement [10] as well as in intrafusal muscle fibers encapsulating muscle spindles [30,93,94]. Our findings might therefore have broad implications for understanding neuromechanics of whole-body movements. Simulations are a useful tool to disentangle the relation between muscle mechanisms and whole-body behavior, yet most simulations of whole-body movement are based on Hill-type models and the few simulations that are based on cross-bridge dynamics lack cooperative myofilament dynamics [17]. Some simulations have used phenomenological models of short-range stiffness that require the user to explicitly define the movement history and do not capture the complex relationships between conditioning stretch characteristics, activation level, and short-range stiffness [13,14]. These simulations have shown that modeling the large short-range stiffness in muscles that have been isometric is crucial to explain the response to perturbations of standing [13,95–97], walking [98], and reaching [99]. Furthermore, capturing the history dependence of short-range stiffness is key to explaining joint hyper-resistance in neurological populations [14,100,101]. Yet, understanding the role of short-range stiffness during continuous or cyclic movement such as locomotion requires a model of muscle dynamics that captures how short-range stiffness varies with movement history and activation levels. The cross-bridge model with cooperative myofilament dynamics proposed here could fill this gap, especially since describing the cross-bridge distribution by a Gaussian yields high accuracy at low computational demand. The logical next step is therefore to implement these models in whole-body musculoskeletal simulations to simulate short-range stiffness during movement.

## Conclusion

This study aimed to identify a muscle model based on its ability to capture skeletal muscle’s history-dependent reduction in short-range stiffness, referred to as muscle thixotropy. We found that incorporating cooperative dynamics for myofilament activation improved predictions of history-dependent short-range stiffness reductions. Using a Gaussian approximation of the cross-bridge distribution seems to be a good trade-off between model accuracy and computational demand, facilitating integration in musculoskeletal simulation. Musculoskeletal simulations based on cross-bridge muscle models with cooperative myofilament dynamics could elucidate the role of history-dependent short-range stiffness in whole-body movement.

## Methods

We tested four different cross-bridge muscle models of increasing complexity against *in vitro* data from permeabilized rat soleus muscle fibers for a range of behaviorally relevant stretches. Data included force and length trajectories of permeabilized rat soleus muscle fibers (n = 11) for stretch-shortening-stretch protocols at 4–6 different calcium concentrations, 4–7 different amplitudes of the first stretch, and 5–7 isometric recovery times before the second stretch, for a total of 80–196 trials per fiber. We tested four cross-bridge models: (1) two cross-bridge states (i.e., attached, detached) without cooperative myofilament activation (2-state XB model), (2) two cross-bridge states (i.e., attached, detached) with cooperative thin – but not thick - filament activation (2-state XB coop model), (3) three cross-bridge states (i.e., attached, detached, super-relaxed) with cooperative thin- and thick filament activation (3-state XB coop model), and (4) four cross-bridge states (i.e., attached, detached, super-relaxed, and forcibly-detached) with cooperative thin- and thick filament activation (4-state XB coop model). We evaluated all models both with and without a Gaussian approximation of the cross-bridge distribution. Model parameters were fit on a subset of data (two stretch-shortening-stretch protocols across all activation levels) and evaluated on the remaining data. We first describe the experimental data in more detail and then describe the cross-bridge muscle models.

### Experimental data

**Fig 9:**
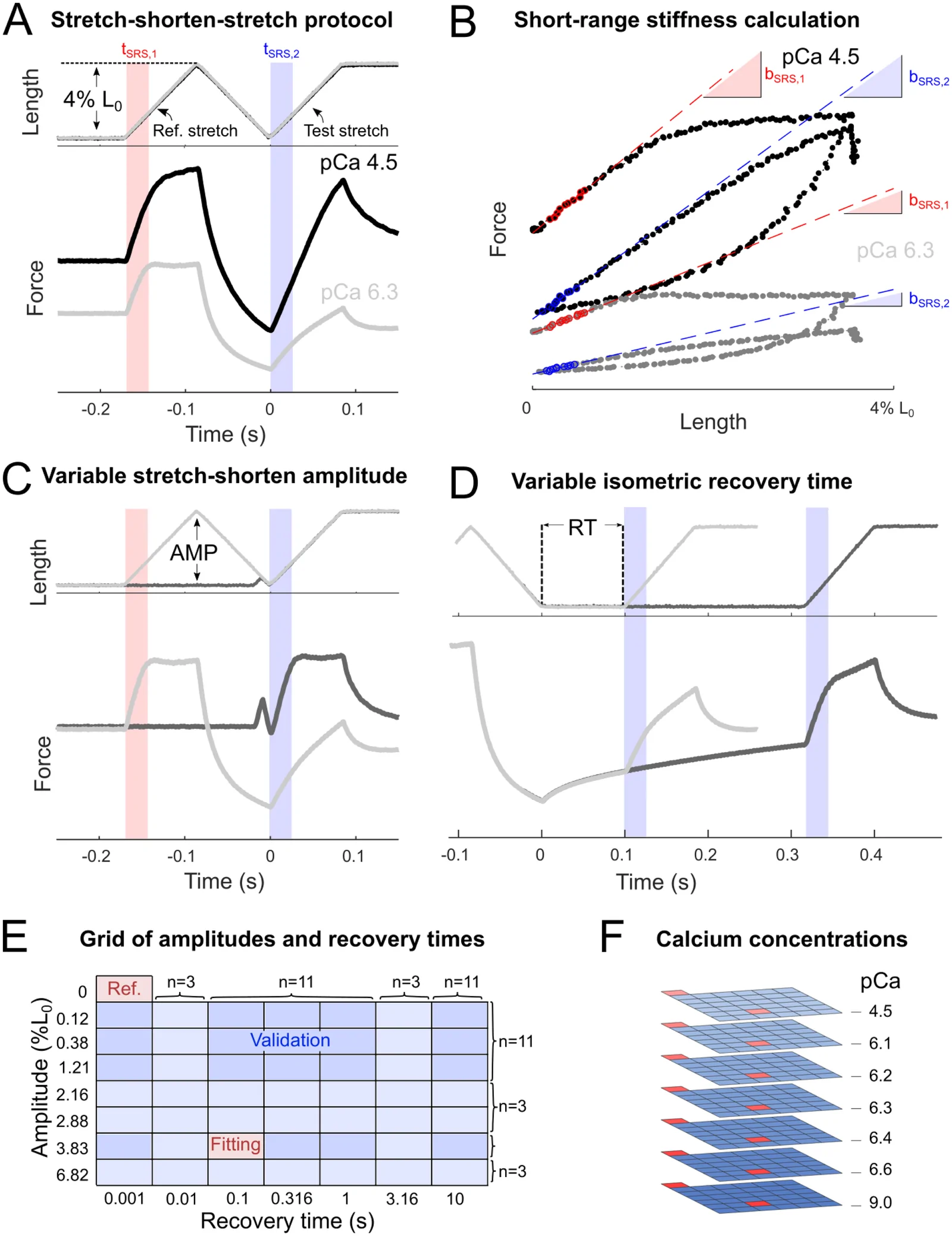
Experimental data. Data were obtained using stretch-shortening-stretch protocols that differed in conditioning stretch amplitude, recovery time, and calcium concentration. **A.** Muscle fiber length and force trajectories for stretch-shorten-stretch protocols at constant stretch amplitude and recovery time but variable calcium concentration (pCa). **B.** Short-range stiffness was defined as the slope of the force versus length data during the first 10 ms after stretch onset b_SRS_. Red and blue dots indicate data points used to determine stiffness during the conditioning stretch and test stretch, respectively. **C.** Trajectories for protocols at constant calcium concentration and recovery time but variable conditioning stretch amplitude (AMP). **D.** Trajectories for protocols at constant amplitude and calcium concentration but variable recovery time (RT). **E.** Grid of tested amplitudes and recovery times. Models were fitted on data from two trials per calcium concentration (red) and evaluated on data from the remaining trials (blue). Most fibers (n = 8) had grids with 4 AMPs and 5 RTs. A subset of fibers (n = 3) had grids with 7 AMPs and 7 RTs. Empirical and modeled short-range stiffness during stretch-shortening-stretch protocols was expressed relative to short-range stiffness during a reference stretch trial without prior stretch-shortening (Ref). **F.** Grids of amplitudes and recovery times were repeated for up to 7 different calcium concentrations.

We fitted and evaluated cross-bridge muscle models on existing data from a broad range of stretch-shortening-stretch trials at various calcium concentrations on eleven permeabilized rat soleus muscle fibers obtained from 2 adult female Sprague–Dawley rats [16]. Rat soleus muscle fibers were chosen because they exhibit greater history dependence and are more robust to long experimental protocols compared with faster muscle fibers [9]. Fibers had a rate of tension regeneration of less than 2 s^-1^, consistent with that of slow-twitch fibers [9]. Permeabilized muscle fiber bundles were stored in a glycerol solution at −20°C for no more than 1 month prior to use. A mean temperature of 22°C was maintained during the experiments. The experimental trials featured an isometric phase, followed by an isokinetic conditioning stretch, an isokinetic shortening phase back to the pre-conditioning length, an isometric recovery phase, and an isokinetic test stretch followed by another isometric phase (Fig 9). All protocols started at a reference length $L_{\text{0}}$ of 0.8 ± 0.2 mm (mean ± s.d.), corresponding to a mean sarcomere length of 2.60 ± 0.05 µm (mean ± s.d., microscopy). All stretches had a strain rate of 0.45 L_0_/s, corresponding to ~35% of the expected unloaded shortening velocity of these fibers [102]. Trials differed in their calcium concentration (pCa, Fig 9A and 9B), conditioning stretch-shortening amplitude (AMP, Fig 9C), and isometric recovery time (RT, Fig 9D). Most fibers (n = 8) were tested at 4 AMPs and 5 RTs at 5–6 pCas. A subset of fibers (n = 3) was tested at 7 AMPs and 7 RTs for 4–5 pCas (Fig 9E and 9F). Some AMP-RT-pCa combinations were excluded to avoid run-down of the fibers. All fibers also had a reference condition, without a conditioning stretch. To reduce the chance of early run-down, the stretch-shorten trials at pCa 4.5 were performed last. The pCa 4.5 conditions of two fibers were excluded because the isometric force at the onset of the stretch-shortening-stretch trials was less than 50% of that during an initial reference trial at the same calcium activation level, indicating run-down. Averaged over the remaining trials, isometric force at stretch onset was within 1% of that measured during the reference trials, indicating that the fiber was still intact. The reader is referred to the original publication [16] for more details on experimental data.

#### Fitting versus validation data.

We selected two stretch-shortening-stretch protocols across activation levels for fitting model parameters (“Fitting”, Fig 9E). The first condition was a large conditioning stretch amplitude (AMP = 3.83% $L_{\text{0}}$) and a short recovery time (RT = 0.1 s), resulting in large short-range stiffness reductions. The second fitting condition was the reference condition without conditioning stretch (AMP = 0% $L_{\text{0}}$), resulting in high short-range stiffness. Whereas the first stretch was identical in both trials, it was followed by shortening in the presence of a conditioning stretch and by an isometric contraction in the reference condition. The remaining trials were used to validate model predictions (“Validation”, Fig 9E).

### Data processing

Data was analyzed and processed using custom MATLAB-based software (MathWorks, Natick, MA, USA). Empirical forces during experimental trials were scaled such that the initial isometric force equaled that during a corresponding pCa-matched isometric trial to remove potential run-down effects. To account for differences in specific tension between fibers, empirical forces were then normalized with respect to the maximal isometric force $F_{\text{0}}$, obtained during an isometric contraction at the highest calcium concentration (i.e., pCa = 4.5). Short-range stiffness was quantified as the average slope b_SRS_ of the force-length curve during the first 0.01 s after stretch onset, determined by fitting a straight line (MATLAB’s *polyfit*) to the force versus length data (see Fig 9B). It was challenging to reliably calculate short-range stiffness in passive conditions, where forces were so small that they were of similar magnitude as background noise. The chosen time window therefore was a trade-off between including more data points to improve the reliability of the short-range stiffness estimates, and preventing that force leveled off within the window. The chosen window of 0.01 s was well within the range of previous research [16,103,104]. Muscle thixotropy was determined from the reduction in short-range stiffness, quantified as the short-range stiffness of the test stretch relative to that of an unconditioned stretch. Relative short-range stiffness values of 0 and 1 respectively indicate maximal and minimal muscle thixotropy. To account for differences in calcium sensitivity between fibers, activation level was defined as the isometric force reached relative to that at pCa 4.5. Four activation categories were used to compute fiber-average results: Passive (<5% activated), Low (5 – 30% activated), Intermediate (30 – 70% activated) and High (>70% activated). Overall, the dataset included 84 experimental conditions tested in all 11 fibers (i.e., 4 activation levels × [4 AMPs × 5 RTs + 1 reference trial]), and 200 experimental conditions tested in a subset of 3 fibers (i.e., 4 activation levels × [7 AMPs × 7 RTs + 1 reference trial]).

### Cross-bridge models

We explored four cross-bridge muscle models that describe the mechanisms that are thought to underly muscle short-range stiffness and its history dependence. Considering the potential role of cooperative myofilament dynamics in muscle thixotropy, we evaluated a previously described model that includes these dynamics [20], referred to as 3-state XB coop model, alongside a model without such dynamics [18,40], referred to as 2-state XB model. We also evaluated an intermediate model with 2 cross-bridge states and thin filament cooperative dynamics, referred to as 2-state XB coop model. Lastly, we evaluated the effects of adding a forcibly-detached cross-bridge state, referred to as 4-state XB coop model. Most parameter values were obtained from literature (Table 2), with the remainder obtained from fitting on a subset of data (Table 3).

**Table 2:** Fixed parameter values. ^1^T: temperature (295 K), $k_{B}$: Boltzmann constant (1.380649 × 10^-23^ m^2^ kg s^-2^ K^-1^). ^2^ ~ 200 times higher than regular attachment/detachment rates. ^3^ Corrected for temperature difference.

Symbol	Meaning	Value	Reference
$d_{ps}$	Cross-bridge power stroke step size	10 nm	[105]
$k_{CB}$	Cross-bridge stiffness	0.5 pN/nm	[106,107]
w	Standard deviation Brownian motion detached head	3 nm	$\sqrt{\frac{k_{CB}}{k_{B}\cdot T}}$ [20,31] ^1^
$E_{\text{1}}$	Exponential coefficient strain-dependent cross-bridge detachment at larger negative strains	2	[31]
$\Gamma_{\text{1}}$	Detachment rate beyond critical stretch	3000 s^-1^	[39]^2^
$\phi_{\text{1}}$	Reattachment rate of forcibly-detached cross-bridges	1000 s^-1^	[39]^2^
$N_{overlap}$	Fraction of binding sites that are in range of myosin heads	1	See text
$k_{\text{off}}$	Thin filament deactivation rate constant	80 s^-1^	[108]^3^
$k_{\text{1}}$	Thick filament activation rate constant	6.17 s^-1^	[20]
$k_{\text{2}}$	Thick filament deactivation rate constant	200 s^-1^	[20]
β	Fraction of attached myosin heads relative to the total attached during an maximal isometric contraction	0.5	[109]

**Table 3:** Parameter values for each model, expressed as median across fibers (n = 7).

Parameter	Units	Hill (no SE)	Hill (with SE)	2-state XB	2-state XB coop	3-state XB coop	4-state XB coop
$n_{H}$	−	4.4	3.3	3.1			
$\text{C}{\text{a}}_{\text{50}}$	μM	0.77	0.78	0.83			
$v_{max}$	$x_{ps}\cdot s^{-\text{1}}$	440	76				
ρ	(${x_{ps}}^{-\text{1}})$		0.21	0.24	0.30	0.24	0.26
σ	$F_{\text{0}}$		0.19	0.19	0.02	0.2	0.15
$k_{PE}$	$F_{\text{0}}\cdot$(${x_{ps}}^{-\text{1}})$	0.03	0.006	0.002	0.004	0.002	0.002
$f_{\text{1}}$	$s^{-\text{1}}$			52.0	67.1	84.5	79.5
$g_{\text{1}}$	$s^{-\text{1}}$			4.0	3.4	3.5	6.4
$g_{\text{2}}$	$s^{-\text{1}}$			21.1	26.6	23.8	21.2
$E_{\text{2}}$	−			-0.6	-0.4	-0.6	-0.7
$k_{on}$	$\mu M\cdot s^{-\text{1}}$				41.4	44.4	43.9
$k_{c}$	−				15.4	3.8	4.0
$k_{F}$	${\left({\tilde{F}}_{CB,\text{ max}}\right)}^{-\text{1}}$					210	223
$x_{crit}$	$x_{ps}$						2.5

#### Cross-bridge cycling.

All cross-bridge muscle models considered here are based on A.F. Huxley’s [34] 2-state cross-bridge model, which tracks the fraction of formed cross-bridges n for each cross-bridge strain xover time t, denoted as n(x,t). The original formulation distinguishes between contributions of the temporal and spatial dynamics of n:


$$\begin{array}{c} \frac{\text{d}n}{\text{d}t}=\frac{\partial n}{\partial t}+\frac{\partial n}{\partial x}\cdot \frac{\partial x}{\partial t} \end{array}$$ (1)

Huxley’s model originally only considered muscle fibers at maximal activation levels, but has been adapted over the years to incorporate submaximal activation of the thin filament [35] and thick filament [20]. Here, we adopt the following version of the temporal dynamics [20]:


$$\begin{array}{c} \frac{\partial n}{\partial t}=M\cdot f(x)\cdot [N_{on}-\int n(x)\text{d}x]-g(x)\cdot n(x) \end{array}$$ (2)

M is the fraction of myosin heads in the disordered-relaxed state (see ‘Thick filament activation’, below), $N_{on}$ is the fraction of thin filament binding sites that is in a state in which cross-bridge attachment is possible (see ‘Thin filament activation’, below), the integral ∫n(x)dx indicates the fraction of formed cross-bridges, and f(x) and g(x) are the strain-dependent attachment and detachment rate functions. In line with previous cross-bridge models [20,34,35,40], we assume that cross-bridges instantaneously undergo a power-stroke upon attachment, increasing their strain with step size $d_{ps}$, set to 10 nm here [105]. In Eq. 2, cross-bridge strain x is normalized and centered relative to the assumed step size of the cross-bridge power stroke $d_{ps}$:


$$\begin{array}{c} x=\frac{d-d_{ps}}{d_{ps}} \end{array}$$ (3)

Here, d and x are the absolute cross-bridge elongation and the cross-bridge strains, respectively. Because of centering (i.e., Eq. 3), normalized cross-bridge force equals the sum of the zero-order and first-order moments of the cross-bridge distribution n(x):


$$\begin{array}{c} {\tilde{F}}_{CB}=\sum_{\lambda =\text{0}}^{\text{1}}\int x^{\lambda }\cdot n(x)\text{d}x \end{array}$$ (4)

Here, ${\tilde{F}}_{CB}$ is the cross-bridge force, normalized with respect to the force obtained when all cross-bridges are attached with a strain equal to that of the power stroke (i.e., $d=d_{ps}$, and x=0).

In line with a previous formulation [31], the cross-bridge attachment function f(x) is a Gaussian and the detachment function g(x) is the sum of two exponentials:


$$\begin{array}{c} f(x)=\frac{f_{\text{1}}}{\sqrt{\text{2}\pi }\cdot w}\cdot e^{-\frac{x^{\text{2}}}{\text{2}{\cdot w}^{\text{2}}}} \end{array}$$ (5)

$$\begin{array}{c} g(x)=\sum_{i=\text{1}}^{\text{2}}g_{i}\cdot e^{-x\cdot E_{i}} \end{array}$$ (6)

The Gaussian attachment function f(x) is specified by two parameters: surface area $f_{\text{1}}$ and standard deviation w. The exponential detachment function g(x) is scaled by rate constants $g_{\text{1}}$ and $g_{\text{2}}$, and shaped by dimensionless exponential strain dependence coefficients $E_{\text{1}}$ and $E_{\text{2}}$. A Gaussian formulation of the attachment function was chosen because it reflects the Brownian motion of the myosin head before attachment. Cross-bridge stiffness $k_{CB}$ was assumed to be 0.5 pN/nm [106,107], yielding a cross-bridge power-stroke force of 5 pN and a maximum work output of 50 zJ, both in agreement with previous estimates [110]. In line with previous model formulations [20,31], the Brownian motion of the myosin heads had a standard deviation w equal to $\sqrt{\frac{k_{CB}}{k_{B}\cdot T}}$, where $k_{B}$ is the Boltzmann constant and T is the temperature. Cross-bridge stiffness and experimental temperature (22 °C) yielded w = 3 nm. The exponential increase in detachment function g(x) with larger negative strains reflects the force dependence of the ADP release rate, which is assumed to have a dimensionless coefficient $E_{\text{1}}$ of 2 [31]. The exponential increase in detachment function g(x) with larger positive strains reflects strain induced cross-bridge detachment [31], with a dimensionless coefficient $E_{\text{2}}$. Optionally, a separate ‘forcible detachment’ pathway was included, similar to that of Lombardi and Piazzesi [39]:


$$\begin{array}{c} \frac{\text{d}R}{\text{d}t}=\int \Gamma (x)\cdot n(x)\text{ d}x-\phi (x)\cdot R \end{array}$$ (7)

Here, R is the fraction of cross-bridges in the forcibly-detached state, while Γ(x) and ϕ(x) are the forcible detachment and reattachment functions, respectively:


$$\begin{array}{c} \Gamma (x)=\Gamma_{\text{1}}\cdot \frac{\text{tanh}[\left(x-x_{c}\right)\cdot s]+\text{1}}{\text{2}} \end{array}$$ (8)

$$\begin{array}{c} \phi (x)=\frac{\phi_{\text{1}}}{\sqrt{\text{2}\pi }\cdot w}\cdot e^{-\frac{x^{\text{2}}}{\text{2}{\cdot w}^{\text{2}}}} \end{array}$$ (9)

Reattachment function ϕ(x) has the same form as attachment function f(x), but with integral $\phi_{\text{1}}$ instead of $f_{\text{1}}.$ $\phi_{\text{1}}$ was set at 1000 s^-1^, qualitatively matching a previous model formulation [39] in which the reattachment rate constant was ~ 200 times greater than the regular attachment rate constant. Forcible detachment function Γ(x) has a step-like shape, specified by critical strain $x_{c}$, steepness s set to 20, and step-height $\Gamma_{\text{1}}$ set to 3000 s^-1^. The full temporal dynamics of the cross-bridge model incorporating forcibly-detached cross-bridges are described by:


$$\begin{array}{c} \frac{\partial n}{\partial t}=M\cdot f(x)\cdot [N_{on}-\int n(x)dx]-g(x)\cdot n(x)-\Gamma (x)\cdot n(x)+\phi (x)\cdot R \end{array}$$ (10)

#### Thin filament activation.

In models without cooperative thin filament activation, thin filament activation $N_{on}$ depends on calcium concentration via a sigmoidal saturation function [111]:


$$\begin{array}{c} N_{on}=\frac{N_{overlap}}{\text{1}+{\text{1}\text{0}}^{n_{H}\cdot (\text{pCa}+{\text{log}}_{\text{10}}\left(Ca_{\text{5}\text{0}}\right))}} \end{array}$$ (11)

Here, $N_{overlap}$ is the fraction of binding sites that are in range of myosin heads, $\text{C}{\text{a}}_{\text{50}}$ is the calcium concentration required to achieve 50% of the maximal isometric force and $n_{H}$ sets the steepness of the sigmoid. Considering the experiments were performed around optimum length, $N_{overlap}$ was set to 1 (i.e., the dependence of force on sarcomere length was neglected here). In models with cooperative thin filament activation, thin filament dynamics are implemented using the following state equation [20]:


$$\begin{array}{c} \frac{\text{d}N_{on}}{\text{d}t}=k_{on}\cdot {\text{1}\text{0}}^{-\text{pCa}}\cdot \left(N_{overlap}-N_{on}\right)\cdot \left(\text{1}+k_{c}\cdot \frac{N_{on}}{N_{overlap}}\right) \end{array}$$

$$\begin{array}{c} -k_{off}\cdot \left[N_{on}-\int n(x)\text{d}x\right]\cdot \left(\text{1}+k_{c}\cdot \frac{N_{overlap}-N_{on}}{N_{overlap}}\right) \end{array}$$ (12)

Here, $k_{on}$ and $k_{off}$ are activation and deactivation rate constants, respectively. $k_{\text{off}}$ was set to 80 s^-1^, based on temperature corrected estimates of the calcium-troponin dissociation rate in mouse soleus muscle fibers [108]. pCa specifies the calcium concentration ([Ca^2+^] $={\text{1}\text{0}}^{-\text{pCa}})$. $k_{c}$ is a constant that defines the strength of thin filament cooperativity. If $k_{c}$ is greater than zero, active binding sites induce other sites to activate, whereas inactive sites accelerate deactivation. The term ($N_{on}-\int n(x)dx$) assures that the thin filament can only deactivate when no cross-bridges are formed.

#### Thick filament activation.

In models with cooperative thick filament activation, thick filament dynamics are implemented using the following state equation:


$$\begin{array}{c} \frac{\text{d}M}{\text{d}t}=k_{\text{1}}\cdot \left(\text{1}+k_{F}\cdot {\tilde{F}}_{CB}\right)\cdot [\text{1}-\int n(x)\text{ d}x-M]-k_{\text{2}}\cdot M \end{array}$$ (13)

Here, $k_{\text{1}}$ and $k_{\text{2}}$ are forward and backward rate constants, respectively. Parameter $k_{F}$ specifies the sensitivity of the forward rate to normalized cross-bridge force ${\tilde{F}}_{CB}$, reflecting the effects of filament stress on the molecular interactions stabilizing the super-relaxed state [23]. The forward and backward rates $k_{\text{1}}$ and $k_{\text{2}}$ were set to 6.17 s^-1^ and 200 s^-1^, based on previous estimates [20]. In models without cooperative myofilament activation, cross-bridges are not allowed to leave the disordered-relaxed state and enter the super-relaxed state. This is implemented by setting $k_{\text{2}}=\text{0}$, and setting the initial value of M equal to 1. This effectively reduces the number of cross-bridge states by 1.

#### Interface with elastic elements.

**Fig 10:**
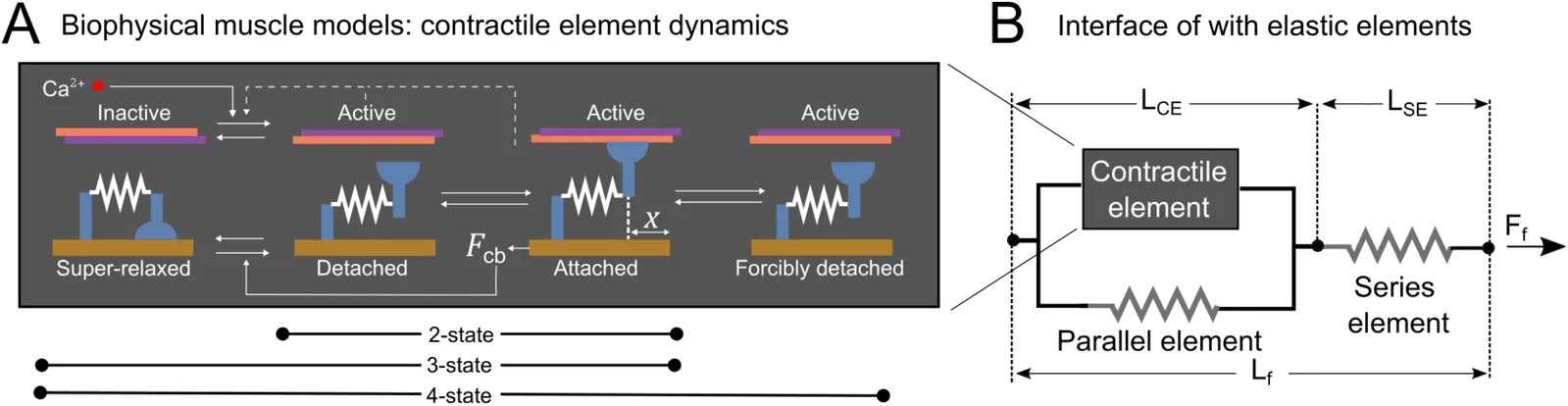
Cross-bridge muscle model dynamics and interface with elastic elements. **A.** Cross-bridge muscle models distinguish between dynamics for thin filament activation, thick filament activation, and cross-bridge cycling. Thin filament activation depends on myoplasmic calcium (Ca^2+^), and is optionally mediated by the fraction of active thin filament binding sites and the fraction of formed cross-bridges (dashed lines). Thick filament activation is mediated by cross-bridge force $F_{CB}$ (dashed lines), which depends on cross-bridge strain x. **B.** Cross-bridge muscle model dynamics describe the contractile element (CE), which is interfaced with series and parallel elastic elements (SE and PE).


Cross-bridge cycling dynamics are interfaced with parallel and elastic tissues (Fig 10), similar to previously described [40,41]. We used a common configuration in which the contractile element (CE) is combined with both in-parallel (PE) and in-series elastic elements (SE). The forces and lengths of these elements are related as follows:

$$\begin{array}{c} {F_{f}=F_{SE}=F}_{CE}+F_{PE} \end{array}$$ (14)

$$\begin{array}{c} L_{PE}=L_{CE} \end{array}$$ (15)

$$\begin{array}{c} {L_{f}=L_{SE}+L}_{CE} \end{array}$$ (16)

$F_{f}$, $F_{SE}$, $F_{PE}$ and $F_{CE}$ are the forces produced by the fiber, the SE, the PE, and the CE, respectively. $L_{f}$, $L_{SE}$, $L_{PE}$, and $L_{CE}$ are the lengths of the fiber, the SE, the PE and the CE, respectively. We used a scale factor γ to relate CE length changes to cross-bridge strain changes:


$$\begin{array}{c} \Delta L_{CE}=\gamma \cdot \Delta x \end{array}$$ (17)

$$\begin{array}{c} \gamma =\text{2}\cdot N\cdot d_{ps} \end{array}$$ (18)

Here, N is the number of sarcomeres in the fiber, which is counted experimentally using microscopy. For this dataset, N was equal to 306 ± 78 (mean ± s.d.).

The parallel element represents compliant structures within the half-sarcomere that act in parallel with the cross-bridge (e.g., titin). It is modeled as a linear spring for lengths greater than resting length $L_{PE,\text{0}}$, specified by two parameters, i.e., stiffness $k_{PE}$ and resting length $L_{PE,\text{0}}$. Similarly to previous formulations [112,113], a ‘softplus’ function was used to smoothen the transition between the two regions (i.e., $L_{PE}<L_{PE,\text{0}}$ and $L_{PE}>L_{PE,\text{0}}$):


$$\begin{array}{c} F_{PE}=k_{PE}\cdot \text{ln}\left(\text{1}+e^{\frac{L_{PE}-L_{PE,\text{0}}}{\gamma }}\right) \end{array}$$ (19)

The series element represents compliant structures outside the half-sarcomere that act in series with the cross-bridges, such as those arising from sarcomere heterogeneity - where half-sarcomeres in the middle of the preparation shorten by stretching half-sarcomeres near the ends [114,115] - and compliance of the fiber attachment. Similarly to previous formulations [112,113], it is modeled as an exponential spring, specified by two parameters, i.e., coefficient σ and exponent ρ:


$$\begin{array}{c} F_{SE}=\sigma \cdot \left[e^{\frac{\rho }{\gamma }\cdot {(\text{L}}_{\text{SE}}-L_{SE,\text{0}})}-\text{1}\right] \end{array}$$ (20)

For Hill-type models, the force of the contractile element can be computed from:


$$\begin{array}{c} F_{CE}=N_{on}\cdot f_{v}\left(\frac{dx}{dt}\right) \end{array}$$ (21)

Here, $f_{v}$ is the Hill-type force-velocity relation [43]. For cross-bridge models, the force of the contractile element represents the force generated by cross-bridges, which is proportional to the cross-bridge force defined above:


$$\begin{array}{c} F_{CE}=\frac{{\tilde{F}}_{CB}}{\beta }. \end{array}$$ (22)

Here, β represents the fraction of attached myosin heads during an isometric contraction at maximal activation relative to the total number of myosin heads. Eq. 22 assures that $F_{CE}=\text{1}$ when the thin filament is maximally activated (i.e. pCa = 4.5) and the fiber is isometric at optimum length. Cross-bridge attachment fraction β was set to 0.5 [109]. As described previously [40,41], there is only one lengthening velocity of the contractile element $\frac{{dL}_{CE}}{dt}$ that assures that the force constraint (Eq. 14) is met. This velocity $\frac{{dL}_{CE}}{dt}$ can be computed from the model states and inputs [40,41]:


$$\begin{array}{c} \frac{{\text{d}L}_{CE}}{\text{d}t}=\frac{\frac{{\text{d}L}_{f}}{\text{d}t}\cdot \frac{{\text{d}F}_{SE}}{\text{d}L_{SE}}-\beta \zeta_{\text{0}}}{\frac{\beta }{\gamma }\int n(x)\text{ dx}+\frac{{\text{d}F}_{SE}}{\text{d}L_{SE}}+\frac{{\text{d}F}_{PE}}{\text{d}L_{PE}}} \end{array}$$ (23)

Here, $\frac{{\text{d}L}_{f}}{\text{d}t}$ is the fiber velocity, which is imposed experimentally and therefore treated as input. $\zeta_{\text{0}}$ is the zero-order moment of the cross-bridge distribution’s partial derivative with respect to time $\frac{\partial n}{\partial t}$, which can be computed from n(x) and thin- and thick filament activation (i.e., part of Eq. 2 left from the spatial derivative term).

$F_{CE}$ can be computed from n(x), and is therefore also known. Altogether, given fiber length $L_{f}$ (input) and using Eqs. 19 and 20, Eq. 14 only contains one unknown. Solving the force equilibrium (Eq. 14) given the SE and PE force-length relations (Eqs. 19 and 20) yields $L_{CE}$, $L_{PE}$ and $L_{SE}$, and after differentiating Eqs. 19 and 20 with respect to length, $\frac{{\text{d}F}_{PE}}{\text{d}L_{PE}}$ and $\frac{{\text{d}F}_{SE}}{\text{d}L_{SE}}$. Thus, all variables in Eq. 23 can be determined.

### Solution methods

We evaluated all models with both a discretized and a Gaussian-approximated cross-bridge distribution. We also compared two types of discretization methods, namely one in which the partial differential equation (i.e., Eq. 1) is discretized with respect to both temporal and spatial dynamics (referred to as “traditional solution method”), and one in which this partial differential equation is transformed into a set of ordinary differential equations, thereby separating the temporal and spatial dynamics (referred to as “method of characteristics”). To date it remains unclear how the computation cost and accuracy of these methods compare. We therefore evaluated one cross-bridge model (3-state XB coop model) with both the Gaussian-approximated method, and both discretization methods for a typical example experimental condition (AMP = 3.83%L_0_, RT = 10^-3^ s, pCa = 6.1).

#### Solution methods based on discretization.

Discretization methods used here rely on solving the partial differential equation describing cross-bridge dynamics (Eq. 1) by discretizing the strain distribution x.

The method of characteristics transforms the partial differential equation into ordinary differential equations, thereby separating the temporal and spatial dynamics of the cross-bridge distribution n(x)[35]. The first equation describes the change in the cross-bridge strain vector x as a function of time:


$$\begin{array}{c} \frac{\text{d}x}{\text{d}t}=\frac{\text{1}}{\gamma }\cdot \frac{{\text{d}L}_{CE}}{\text{d}t}\cdot I \end{array}$$ (24)

Here, $\frac{\text{d}x}{\text{d}t}$ is the temporal derivative of the strain vector x and I is a vector of size x with all entries equal to 1. The second equation describes the change in the cross-bridge distribution n(x) due to attachment and detachment of cross-bridges:


$$\begin{array}{c} \frac{\text{d}n}{\text{d}t}=M\cdot f(x)\cdot [N_{on}-\int n(x)dx]-g(x)\cdot n(x) \end{array}$$ (25)

The attachment function f(x) dictates at which strain values cross-bridges can attach (i.e., n differs from zero). Theoretically, n(*x*) can be non-zero for all strain values considering that f(x) is a Gaussian. For practical reasons, we only considered values of f(x) > 0.03 $\frac{f_{\text{1}}}{\sqrt{\text{2}\pi }\cdot w}$ meaningful and hence we assumed that n can be non-zero over the range of x-values between ±3w. In the methods of characteristics, the values of the vector x change during a simulation (Eq. 24) and we need to guarantee that the vector x always contains the interval ±3w where n might differ from zero. Therefore, x should range from $-\text{3}\cdot w+\frac{\text{1}}{\gamma }\cdot \Delta L_{CE,\text{min}}$ to $\text{3}\cdot w+\frac{\text{1}}{\gamma }\cdot \Delta L_{CE,\text{max}}$, where $\Delta L_{CE,\text{min}}$ and $\Delta L_{CE,\text{max}}$ respectively denote the minimum and maximum values of the CE length change during the simulation. Based on the imposed fiber length changes and the obtained SE force-length relation, we found that a range of ±15 $d_{ps}$ was sufficient to meet this requirement.

The traditional solution method tested here [8,9,28,39] discretizes n with respect to *x* and *t*. In this case, the vector *x* is cons*t*ant. Applying the previously mentioned criterion, x should range from −3·w to 3·w. The smaller range of x allows for a smaller number of discretized values of n compared to the method of characteristics, which reduces computational demand. However, unlike the method of characteristics, this alternative solution method requires that a displaced strain vector $x^{+}$ is determined at each time step:


$$\begin{array}{c} x^{+}=x+\Delta x \end{array}$$ (26)

Displacement Δx can be computed from the change in CE length (similar to Eq. 24):


$$\begin{array}{c} \Delta x=\frac{\text{1}}{\gamma }\cdot {\Delta L}_{CE}\cdot I \end{array}$$ (27)

Because n is expressed as a function of x (which is constant in this method), this method requires that n is interpolated from the displaced vector $x^{+}$ back to the discretization vector x. Here, we used linear interpolation (MATLAB’s *interp1*) in line with previous implementations [20,28]. This interpolation has a computational cost that is incurred at each time step, which should result in a higher processing time to simulate a model with a given number of strain bins in x compared with the method of characteristics. To date, it is not clear how the computational demand of this alternative method compares to that of the method of characteristics.

#### Gaussian approximated solution method.

In the approximated solution method, the distribution of formed cross-bridges n(x) is described by a Gaussian:


$$\begin{array}{c} n(x)=\frac{Q_{\text{0}}}{\sqrt{\text{2}\pi }\cdot q}\cdot e^{-\frac{{\left(x-p\right)}^{\text{2}}}{\text{2}{\cdot q}^{\text{2}}}} \end{array}$$ (28)

p and q are the distribution’s mean and standard deviation, respectively. As shown by Zahalak [35], applying a Gaussian approximation to Huxley’s [34] state equation yields a set of three ordinary differential equations. Using the formulation adopted here (Eq. 7), the following three equations can be derived:


$$\begin{array}{c} \frac{\text{d}Q_{\lambda }}{\text{d}t}=M\cdot C_{\lambda }\cdot \left(N_{on}-Q_{\text{0}}\right)-\theta_{\lambda }\cdot Q_{\text{0}}-{\Omega_{\lambda }\cdot Q}_{\text{0}}+\beta_{\lambda }\cdot R+\lambda \cdot \frac{\text{d}x}{\text{d}t}\cdot Q_{\lambda -\text{1}}\text{ for }\lambda =\text{1},\text{2},\text{3} \end{array}$$ (29)

$Q_{\lambda }$ is the λ-order moment of the cross-bridge distribution n(x), with $Q_{-\text{1}}$ set equal to 0. $C_{\lambda }$ and $\beta_{\lambda }$ are the λ-order moments of the attachment functions f(x) and ϕ(x), respectively. $\theta_{\lambda }$ and $\Omega_{\lambda }$ are the λ-order moments of the product of a surface area normalized cross-bridge distribution and the detachment functions g(x) and Γ(x), respectively. For the rate functions used here, the moments $C_{\lambda }$, $\theta_{\lambda }$, $\Omega_{\lambda }$ and $\beta_{\lambda }$ have analytical solutions (see S1 Appendix). If the forcibly-detached state is not included, Eqs. 29 simplify to:


$$\begin{array}{c} \frac{\text{d}Q_{\lambda }}{\text{d}t}=M\cdot C_{\lambda }\cdot \left(N_{on}-Q_{\text{0}}\right)-\theta_{\lambda }\cdot Q_{\text{0}}+\lambda \cdot \frac{\text{d}x}{\text{d}t}\cdot Q_{\lambda -\text{1}}\text{ for }\lambda =\text{1},\text{2},\text{3} \end{array}$$ (30)

Moments $Q_{\lambda }$ relate to the cross-bridge distribution’s mean and standard deviation p and q:


$$\begin{array}{c} Q_{\text{1}}=p\cdot Q_{\text{0}} \end{array}$$ (31)

$$\begin{array}{c} Q_{\text{2}}={(q}^{\text{2}}+p^{\text{2}})\cdot Q_{\text{0}} \end{array}$$ (32)

### Hill-type model

As a benchmark, we also tested two Hill-type models commonly used in movement simulations. In these models, cross-bridge dynamics (Fig 10A) were replaced by a phenomenological force-velocity relation to capture the dependence of force on stretch velocity. We adopted a continuously differentiable formulation of the force-velocity relation using default shape parameters [43]. This formulation was chosen to facilitate parameter estimation through direct collocation. We tested one Hill-type model without a series elastic element, and one Hill-type model with a series elastic element. In contrast to the common interpretation that the series elastic element represents the tendon, here the series elastic element represented fiber compliance.

### Fitted model parameters

The following cross-bridge parameters were fitted on experimental data from the fitting trials for all XB models: $f_{\text{1}}$, $g_{\text{1}}$, $g_{\text{2}}$, and $E_{\text{2}}$. For the 4-state XB coop model, one additional parameter was fitted: critical stretch $x_{c}$. For the models with cooperative thin filament activation, $k_{on}$ and $k_{c}$ were estimated by fitting experimental data. For the Hill-type and (regular) 2-state XB models that lack cooperative thin filament activation, the parameters $n_{h}$ and $\text{pC}{\text{a}}_{\text{50}}$ of the sigmoidal activation function (Eq. 11) were estimated by fitting experimental data. For the models with cooperative thick filament activation, parameter $k_{F}$ was fitted on experimental data from the fitting trials. For the Hill-type model, unloaded shortening velocity $v_{max}$ was fitted on experimental data from the fitting trials. Finally, the resting length $L_{PE,\text{0}}$ and stiffness $k_{PE}$ of the parallel element, and the series elasticity parameters σ and ρ were fitted on experimental data from the fitting trials for each model.

### Parameter estimation

All unknown parameters were estimated by fitting the simulated forces to the empirical forces for two stretch-shortening-stretch protocols across calcium concentration levels for 7 muscle fibers (“Fitting”, Fig 9). Four (out of 11) fibers were excluded from fitting because they did not include stretch-shortening-stretch trials at Intermediate and/or Maximal activation levels, which we found to be key to fitting. Imposed length changes and calcium concentrations were inputs and model parameters were estimated by minimizing the sum-squared difference between the simulated and empirical forces. The time interval considered for fitting ranged from 0.0843 s before the onset of the conditioning stretch until 0.0843 s after the end of the test stretch. We chose 0.0843 s because it corresponds to the duration of the test stretch. For cross-bridge models, parameter estimation was performed using the Gaussian approximated solution method. Optimization problems were solved using direct collocation [CasADi; 116]. The resulting nonlinear optimization problems were solved using IPOPT [117] with MUMPS [118]. All code pertaining to the muscle models is available on GitHub (https://github.com/timvanderzee/biophysical-muscle-model). Model parameter values are reported in Table 3.

### Model evaluation and predictions

#### Forces and history dependence.

The ability of muscle models to capture the empirical data was assessed in two different ways. First, the root-mean-square difference (RMSD) between modeled forces and empirical forces was computed for all experimental trials for each fiber included in the fitting set (n = 7). These force RMSDs $\sigma_{F}$ were computed for three different time intervals: (1) 0.0843 s before the onset of the conditioning stretch until 0.0843 s after the end of the test stretch (i.e., same interval as used for parameter estimation), (2) during the conditioning stretch, and (3) during the test stretch. The force RMSDs $\sigma_{F}$ were expressed as a fraction of the maximal isometric force $F_{\text{0}}$ measured at pCa = 4.5. We predicted that $\sigma_{F}$ would decrease with increasing model complexity. To compare $\sigma_{F}$ between models of increasing complexity, we first ranked models according to the number of model states (Table 4). We then performed paired t-tests to compare $\sigma_{F}$ between models adjacent in the complexity ranking (5 comparisons × 3 types of RMSD). We applied a Bonferroni correction, yielding a probability threshold of p = 0.0033. Second, in addition to comparing model force predictions, model predictions of muscle thixotropy were evaluated. To this end, we computed the RMSDs $\sigma_{SRS}$ between the mean modeled (n = 7) and mean measured (n = 11) relative short-range stiffness across (1) all 84 experimental conditions tested in all 11 fibers, (2) conditions with large history-dependent short-range stiffness reductions, and (3) conditions with small history-dependent short-range stiffness reductions. Based on the experimental results [16], conditions with large short-range stiffness reductions were defined as those with Low or Intermediate activation levels, AMP > 3% L_0_ and RT < 0.5 s. Experimental conditions with small short-range stiffness reductions were defined as the remaining conditions. We anticipated that differences in $\sigma_{SRS}$ between models would be more pronounced for conditions with greater history-dependent stiffness reductions.

**Table 4:** Models of increasing complexity. N is the number of additional states required to track the strain of formed cross-bridges. For discretized solutions methods, N equals the number of strain bins minus 1. For a Gaussian-approximated solution methods, N = 2 (e.g., mean p and standard deviation q).

Model (increasing complexity)	# model states	# fitted parameters k
Hill-type (no SEE)	0	5
Hill-type (with SEE)	1	7
2-state XB	1 + N	10
2-state XB coop	2 + N	10
3-state XB coop	3 + N	11
4-state XB coop	4 + N	12

We computed the Akaike Information Criterion score (AIC) of each model’s short-range stiffness predictions to account for differences in the number of fitted parameters between models (see Table 4):


$$\begin{array}{c} \text{AIC}=\text{2}\cdot k+n\cdot \text{ln}{({\hat{\sigma }}_{SRS}}^{\text{2}}) \end{array}$$ (33)

Here, k is the number of fitted parameters (see Table 4) and n is the number of observations (84). The AIC score was expressed relative to that of the Hill-type model without SEE (ΔAIC). Unless mentioned otherwise, results pertain to a solution method based on a discretized cross-bridge distribution (500 bins, method of characteristics) with a strain range of ±15 $\epsilon_{ps}$.

#### Computational demand and accuracy of solution methods.

We compared processing time and accuracy of the two methods based on discretization and the approximated method for a typical example trial (AMP = 3.83%L_0_, RT = 10^-3^ s, pCa = 6.1). For the two discretized models, we evaluated model accuracy and processing time for a range of strain vectors x. Strain vector was varied both by changing the bin width at a given strain range (±15 $\epsilon_{ps})$ and by changing the strain range at a given bin width (0.05 $\epsilon_{ps}$). Accuracy was quantified using the deviation in force prediction with respect to using the method of characteristics with a strain range of ±15 $\epsilon_{ps}$, 10.000 strain bins and Euler integration with a fixed time-step of 10^-4^ s, referred to as force error. For this strain vector, the method of characteristics and the traditional method yielded very similar force output (force error of 0.0025%F_0_). To isolate processing time per function evaluation, we first evaluated all models using Euler integration with a fixed time-step of 10^-4^ s. Next, we compared the processing time of a simulation with variable time steps (using MATLAB’s *ode15s*) for the approximated model and the method of characteristics. We did not evaluate the traditional method with a variable time step because the adopted implementation required a fixed time step.

## Supporting information

S1 Data Short-range stiffness data part 1. (ZIP)

S2 Data Short-range stiffness data part 2. (ZIP)

S1 Appendix Analytical expressions for the spatial integrals. (DOCX)

S1 Fig Modeled (approximated) and measured forces for fitting trials: Hill-type models and a regular 2-state cross-bridge (XB) model. A. Entire protocol. Top row shows measured fiber length changes for a condition with a conditioning stretch (darker solid lines) and without a conditioning stretch (lighter dotted lines). Bottom row shows the corresponding empirical forces (“Data”, grey) at three different calcium concentrations (i.e. pCa 4.5, pCa 6.1 and pCa 9.0), alongside forces of a Hill-type model without a series element (“Hill (no SE)”, light blue), a Hill-type model with a series element (“Hill (with SE), darker blue), and a regular 2-state cross-bridge model (“2-state XB”, red). B. Forces during the conditioning stretch (Cond.) and test stretch (Test) at submaximal activation levels.
(EPS)

S2 Fig Modeled (approximated) and measured forces for fitting trials: cross-bridge (XB) models. A. Entire protocol. Top row shows measured fiber length changes for a condition with a conditioning stretch (darker solid lines) and without a conditioning stretch (lighter dotted lines). Bottom row shows the corresponding empirical forces (“Data”, grey) at three different calcium concentrations (i.e. pCa 4.5, pCa 6.1 and pCa 9.0), alongside forces of a regular 2-state cross-bridge model (“2-state XB”, red), a cross-bridge model with cooperative thin filament activation (“2-state XB coop”, yellow), and a cross-bridge model with both cooperative thin filament activation and cooperative thick filament activation (“3-state XB coop”, purple). B. Forces during the conditioning stretch (Cond.) and test stretch (Test) at submaximal activation levels.
(EPS)

S3 Fig Modeled (approximated) and measured forces for testing trials: cross-bridge (XB) models. A. Entire protocol. Top row shows measured fiber length changes for a condition with a short recovery of 0.001 s (darker solid lines) and with a longer recovery of 0.316 s (lighter dotted lines). Bottom row shows the corresponding empirical forces (“Data”, grey) at three different calcium concentrations (i.e. pCa 4.5, pCa 6.1 and pCa 9.0), alongside forces of a Hill-type model with a series element (“Hill (with SE)”, blue), a cross-bridge model with cooperative thin- and thick filament activation (“3-state XB coop”, purple), and a cross-bridge model with cooperative thin- and thick filament activation, and a forcibly-detached state (“4-state XB coop”, green). B. Forces during the conditioning stretch (Cond.) and test stretch (Test) at submaximal activation levels.
(EPS)

S4 FigRoot-mean-squared deviations (RMSDs) between model (approximated) and experimental forces, normalized with respect to the maximal isometric force F_0_.Force RMSDs $\sigma_{F}$ are shown for Hill-type models without and with series elasticity (“Hill”, light and dark blue), 2-state cross-bridge model (“2-state XB”, red), 2-state cross-bridge model with cooperative dynamics (“2-state coop”, yellow), 3-state cross-bridge model with cooperative dynamics (“3-state coop”, purple) and 4-state cross-bridge model with cooperative dynamics (“4-state coop”, purple). RMSDs are shown for entire stretch-shorten protocol (top row), conditioning stretch (middle row) and test stretch (bottom row). **A**. Averaged over all trials. Asterisks indicate significance at p < 10^-4^. **B.** Effect of calcium activation level at constant amplitude (3.83%L_0_) and recovery time (0.001 s). **C.** Effect of stretch amplitude at constant activation level (pCa 6.3) and recovery time (0.001 s). **D**. Effect of recovery time at constant amplitude (3.83%L_0_) and activation level (pCa 6.3). In panels B, C and D, darker shades indicate lower pCa, larger amplitude, and longer recovery, respectively. In these panels, bars with a white surface area indicate the trial that is common to all three sets of conditions.(EPS)

S5 FigEmpirical and model (approximated) relative short-range stiffness across muscle activation levels, stretch amplitudes and recovery times.Fiber-averaged (n = 7) model predictions and errors are shown for Hill-type model without series compliance (“Hill (no SE)”, light blue), the Hill-type model with series compliance (“Hill (with SE)”, dark blue), the approximated 2-state cross-bridge model (“2-state XB”, red), 2-state cross-bridge model with cooperative dynamics (“2-state coop”, yellow), 3-state cross-bridge model with cooperative dynamics (“3-state coop”, purple), and 4-state cross-bridge model with cooperative dynamics (“4-state coop”, green). Data are shown both averaged across all fibers (n = 11, grey circles), and for individual fibers (black dots). Filled symbols and error bars indicate mean ± s.d. across trials with considerable short-range stiffness reductions, open symbols indicate trials with little to no short-range stiffness reductions. Squares indicate the condition that panels A-C have in common. **A.** Effect of activation level at constant amplitude (3.83%L_0_) and recovery time (0.001 s). **B.** Effect of amplitude at constant activation level (Intermediate) and recovery time (0.001 s). **C.** Effect of recovery time at constant activation level (Intermediate) and amplitude (3.83%L_0_). **D.** Model SRS error, quantified as SRS RMSD $\sigma_{SRS}$, averaged over trials with large short-range stiffness reductions. **E.** $\sigma_{SRS}$ averaged over trials with small short-range stiffness reductions. **D** $\sigma_{SRS}$ averaged over all trials.(EPS)

S6 Fig Empirical and model (approximated) relative short-range stiffness across conditions. Model predictions are averaged over all fibers in the fitting set (n = 7), data are averaged over all data in the dataset (n = 11). **A**. Relative short-range stiffness at submaximal activation levels across amplitudes and recovery times. Relative short-range stiffness is indicated by both the vertical location and surface color. Fitting trials are indicated with stems, connecting to the corresponding dots. The bottom surface indicates the experimental grid of stretch amplitudes and recovery times, distinguishing between conditions measured in all fibers (dark grey lines, n = 11) and conditions measured in a subset of fibers (light grey lines, n = 3), conditions with history-dependent stiffness reductions (grey surface) and conditions without such stiffness reductions (white surface). At submaximal activation levels, empirical short-range stiffness (grey dots) decreases with larger stretch amplitudes and shorter recovery times. This is not captured by the Hill-type model without series compliance (light blue, left), partially captured by the Hill-type model with series compliance (dark blue, middle), and mostly captured by the approximated 3-state XB coop model (purple, right). **B.** Fiber-average time-series of force trajectories during stretch-shorten-stretch with a short recovery time at 3 different activation levels for data (black), Hill-type model without series compliance (light blue, left), Hill-type model with series compliance (dark blue, middle), and approximated 3-state XB coop model (purple, right). Shaded area indicates mean ± 1 standard deviation. **C.** Fiber-average time-series of force trajectories during stretch-shorten-stretch with a long recovery time at 3 different activation levels for data (black), and 3-state XB coop model (purple).
(EPS)

S7 Fig Force predictions for isokinetic stretches across a range of strain rates. Differences between 3-state and 4-state XB coop models are greater for faster stretches than for slower stretches. For faster stretches, the 3-state XB coop model has a larger force overshoot and undershoot compared with the 4-state XB coop model.
(EPS)
