# Supplementary material for: Cross-bridge model for predicting muscle short-range stiffness during movement
Source: PLoS Comput Biol. 2026 Sep 1;22(9):e1014748. doi: 10.1371/journal.pcbi.1014748 (PMC13561430; doi:10.1371/journal.pcbi.1014748)
Supplement: Supplementary file 1 [file pcbi.1014748.s011.pdf]

Review of manuscript PCOMPBIOL-D-26-00059

This study demonstrates – by evaluating various muscle models against empirical data from rat soleus fibers – that cross-bridge models incorporating cooperative activation of both thin and thick myofilaments can accurately predict the history-dependent reduction of short-range stiffness (SRS) known as muscle thixotropy. The authors found that while traditional Hill-type and simpler cross-bridge models fail to capture these dynamics – especially at submaximal activation levels – the inclusion of cooperative dynamics allows the model to match experimental force trajectories across various movement histories. To make these complex models practical for musculoskeletal simulations, the authors implemented a Gaussian-approximated solution method that maintains high accuracy while reducing computational costs significantly compared to traditional discretization methods.
This is an interesting and absolutely relevant approach, mostly new (see below) and thus deserves some attention. The authors have a notable publication history for both the issue of thixotropy and muscle modeling. However, I have several major, medium and minor concerns and questions. Some of these concerns might simply stem from the fact that I am not an expert in Huxley-type modeling, so the authors may feel free to correct me if I am mistaken in certain assumptions or inferences.

Major concerns:

1. One of my main concerns is the – in my opinion unnecessary and biased – 'Hill-bashing', i.e. showing unfair comparisons of Huxley-type and Hill-type models. To elaborate: First off, the first 'subsection' of the discussion ('State-of-the-art musculoskeletal.... properties') claims that no employed Hill-type model could adequately account for thixotropy. It is not said WHERE (i.e. literature sources) those models were employed. Second, comparing a Hill-type model without serial elasticity is just complete bogus, as I personally know of not a single musculoskeletal model that has no SE in it. Third, why is the Hill-type model compared to 3+ state XB models and not likewise modified to account for thin and thick filament activation?
   All in all I plead for the omission of Hill-type models in this manuscript as they add nothing to the story at all, but rather seem to serve as a scapegoat. The enhancement of the Huxley-type model is the story here and this is an important story indeed. If the authors would like to keep it still, it would be enough to show that the standard Hill- and standard Huxley-model equally fail at capturing thixotropy and move on to improve the latter – or improve both models equally and make fair comparisons.

2. Another main concern is the terminology of the XB-model as being "biophysical". In my opinion, it is a phenomenological compartment model that *describes* the physiologically observed transition between XB states, but offers no *mechanistic* explanation. I give some examples: How exactly are the myosin heads assumed to connect to actin? The model offers a kinetic constant, but does it offer an explanation, e.g. via Coulomb forces? No. How exactly is the XB supposed to produce forces? Well it does as per observation and measurement, but does the XB model offer a mechanism? No again. The whole method section from Eqn. 1 to Eqn. 23 offers not a single biophysically interpretable formula. I am not arguing against the Huxley-model per se, as some do (e.g. the recent work of Josh E. Baker), but I would urge the authors to acknowledge the limitations of the model as being of roughly the same descriptive nature as Hill's force-velocity relation. This brings me to my next point.

3. The modeling of cooperativity. I have a rough descriptive idea about cooperative thin filament activation in a sense that Calcium binding to troponin C leads to an easier clearance of tropomyosin along the actin filament (it might be worthwhile to mention that Eqn.11 is already a cooperative calcium activation), yet I am not sure about the exact mechanism here. The authors now propose another descriptive idea by that the cooperative thin-filament activation is further enhanced by already formed XBs. This picture makes sense as bound myosin might hinder tropomyosin from sliding back in place (i.e. deactivate), but whether this is mechanistically justified, I am not sure. Is it somehow backed in the literature, besides the cited modeling paper?
   However, I have some problems with the proposed force-dependent thick-filament activation. While there seems to be a recent push of that idea (by M. Irving and colleagues who term it 'mechano-sensing'), I personally cannot form a coherent mechanistic picture in my head

why simply pulling on the end of myosin should result in the heads to change their state (i.e. leave their ordered state ajar to the myosin backbone). Could the authors provide such a picture or mechanism? Following the literature hint ([19]), I couldn't find any. My personal idea of this observation is that the disorder on the myosin head level stems from changes in electrostatic attraction from the actin – be it that it is moved closer (radial distance decreases during lengthening) or that oppositely-charged binding sites are exposed due to said thin-filament activation (as presented recently by Rockenfeller et al. (2022), DOI 10.1016/j.bpj.2022.04.019). In that regard, the paragraph following line 433 needs to be backed with literature sources.

4. Novelty of the presented approach. I am missing a literature review on how muscle thixotropy has been dealt with hitherto. For example, I found a paper by Altman et al. (2015, DOI 10.1371/journal.pone.0121726) or by Ait-Mou et al. (2016, DOI 10.1073/pnas.1516732113). There may be more. I personally do not now any Hill-type muscle models considering SRS, but a larger body of force enhancement and force depression modeling.

Medium concerns:

1. The overall language of the manuscript is on point. Yet, at some instances, it reads like LLM-assisted writing. Could the authors clarify which parts of the manuscript were written (drafted, proofread, etc) by AI? As the most prominent example, I refer to the first and fourth 'subsections' of the discussion ('State-of-the-art musculoskeletal.... properties' and 'Cooperative myofilament...stiffness"). Here, little to no literature reference is given, most sentences are rather descriptive and some information is given twice, e.g. l.370-71 and l.385 both give a physiological interpretation of the SE as tendon.

2. Lines 59-60 and 84-85: I do not understand why muscle shortening should result in a reduction of the number of cross-bridges? And why should it result in lesser force per cross-bridge? What is the picture behind these statements?

3. Lines 155 and many more: The authors take into account muscle behavior at submaximal Ca levels. First some semantics: In my view, the term "activation" describes a process, while "activity" would describe the state. Would the authors be d'accord to re-term their "activation" accordingly? Second, and this is not semantics, submaximal *activity* poses a problem in terms of the force-length relation in that the muscle no longer operates at the optimal (or plateau) region (see lines 809-810), but on the ascending limb. It is well-established experimentally that the force-length relation shifts to longer lengths with decreasing activity. I was wondering, if the XB-model of the authors was able to capture this effect? For Hill-models there are several ways to enforce this behavior, e.g. Hatze-type length-dependent activation dynamics or Lloyd-like activity-dependent FL curves. I could not find any such modeling in the Hill-type models used by the authors before, e.g. De Groote et al. (2016). Overall, I think this missing factor would be important to consider in the modeling of submaximal contractions.

4. Fig 4: the results shown in this figure are not discussed enough, in my opinion, and raise many questions. In panel A, I completely fail to understand the *huge* variation of relative SRS at zero activity (does activity really have the dimension of a force?). Do the authors have any explanation to offer here? Is it a general uncertainty when stretching a passive muscle? If so, this would query the concept of determining SRS in the first place.
Similarly, in panel B, first relative SRS seems to grow with amplitude and then at one amplitude it is less. Are the measurements for the other amplitudes not available here?
Panel C seems reasonable.
From not being able to see the same points in every panel, I concluded that different experimental boundary conditions were shown in each panel, but the caption does not give me a hint what these would be – contrary to Fig. 5 in which they are clearly stated.

Minor concerns:

1. Fig 1: The second ΔF on the bottom right is not in line with the ΔL above. The last sentence of the caption seems highly speculative, is there any literature backing up this claim?

2. Line 82: Reference [19] is imo not an adequate source to back the claim made here, see also my major concern above.

3. Line 124: It is not until the discussion that titin is mentioned, although it might explain thixotrophy already when implemented. The authors themselves give a hint in their reference [19] to the modeling paper of Ait-Mou et al. (2016).

4. Lines 132-133: This sentence is odd: One models yields better results than another, if we equip it with equations meant to govern the effect of interest. This seems like an obvious statement, see my main concern #1.

5. Lines 203-205: the closing sentence of this paragraph seems a bit of an overstatement. Just because you enhanced the model in a way that it better fit some data does not necessarily imply the physiological consequences you state here. Compare to the statement in lines 469-470.

6. Fig. 4: Some text is hidden behind the graphs, consider replacing it.

7. Lines 222, 227 and some more: Reporting p values in the magnitude of 2^{-50} and below is meaningless, as it lies beyond machine precision in Matlab. I suggest defining some thresholds, e.g. 10^{-4}, below which you speak of highly significant differences. Also, the whole paragraph is hard to read as you report the same values for each overall, conditional and test stretches, with a lot of repetitions. Maybe a table would help here?

8. Lines 221, 226 and more: The concept of $\hat{\sigma}_F$ was hard to follow here, because it does not appear prominently in the text, but rather wordy at the end of the method section. From what I understand, it bears the unit of relative force, where normalization was done post-hoc to a certain reference force. While this seems reasonable, it might make some consequences not very surprising. In lines 236-237, I would expect $\hat{\sigma}_F$ to go up with higher Ca concentration (more force) and larger amplitude (more passive force). This fact is not discussed, if I am not mistaken.

9. Fig. 5: The σ is missing its hat. Also, why are only those bars shown for which 11 samples were taken and not the ones with 3 samples as well?

10. Fig 6 (and others): After trying to zoom in on this figure I found it of poor quality. Are no vector graphic exports available? Also, what confidence intervals are shown with the error bars? 95%? What is the quantitative criterion to label some of them “low HD” and others “high HD”?

11. Line 344: “little influence” is not a quantitative statement.

12. Fig. 8: The mid and right column might better be displayed using a semi-log plot?!

13. L. 387: I do not understand how sarcomere heterogeneity may work as a serial elastic element. The elasticity of the myosin heads itself were shown to work as such an element (see for example DOI 10.1016/j.jtbi.2018.07.023).

14. L. 392: The first half of this sentence seems superfluous.

15. Lines 406ff: While I appreciate the Gaussian approximation to fasten up the computation, I could not find any quantitative rationale (e.g. a Wasserstein metric) supporting the claim that it “adequately represents the shape of the actual distribution”.

16. Line 437: Reference [8] appears twice?!

17. Line 482: From what I see, reference [28] indeed includes both titin and cooperative myosin dynamics?!

18. Line 577: It should read force-TIME curve. Also, can you provide any rationale for choosing the arbitrary 0.01 s?

19. Line 585-586: “ISI” seems to be copy-pasted from a prior publication and should presumably read “RT” here.

20. Line 606 and many others: This formula (Eqn. 2) does not appear in the cited reference [19]?! Further, I personally find the use of curly brackets disconcerting, but it may be just me. Consider using larger parentheses (in particular Eqs 12, 20 and 21, and some in the supplement). Also, operators (such as the “d” in dx) should not be written in cursive.

21. Line 611: I failed to decode the term "assumed size of the XB power stroke". Do you mean the mean displacement?

22. Line 625: Are both E_1 and E_2 necessarily greater than zero?

23. Line 630: Is there a literature reference for Eq. 7?

24. Line 641: The parameter N_{on} is not explained, though it can be inferred from the context.

25. Line 658: This is one of the reasons, I would not term the model "biophysical", as the force dependence is descriptively hard-coded in the model without any mechanistic idea behind.

26. Eqn. 12: Why is Ca concentration not normalized with respect to the maximal concentration?

27. Line 677: Is there an estimate of N made available?

28. Line 690: Before the era of machine learning ('softplus'), PE and SE have been modeled using this exact function by Gerald Loeb and his group (see e.g. Brown et al. 1996).

29. Line 695: Why is the SE force not starting at a slack length like the PE but rather at zero?

30. Lines 701 and 824ff: I do not understand the rationale behind choosing $\beta = 2$.

31. Line 717: Out of ignorance: What are the "two discretization methods"?

32. Line 757: Is "interp1" really efficient? Matlab documentary states it requires "more memory and computation time than nearest neighbor". Or do you require continuity?

33. Line 781ff: Due to the huge amount of parameters presented here, it would be good to shift Table S1 here and also have a second table, presenting all fixed parameters in a concise manner.

34. Line 797: Parameter "r" should be "s".

35. Line 799: What is the literature source for $\phi_1 = 1000\,\mathrm{s}^{-1}$?

36. Line 808: Parameter "h" should be "n_H".

37. Line 811ff: The parameter k_F is *not* force-dependent!

38. Line 822: The FV-relation presented in [36, Eqn S4] looks odd, i.e. a high-parametric log curve. Why not use a Hill-relation and make it adequately activity-dependent?

39. Line 831: Figure 9 instead of Figure 10?

40. Line 834: State which algorithm exactly you were using? From working my way through the Matlab scripts, I assume it is ipopt MUMPS, but the files were not readily executable as you hard-coded local paths on your machine. Could this be fixed? And while I appreciate to have a history of the progress of finding the correct algorithms, it is hard to work one's way through old "test"-files.

41. Line 835: This range is rather arbitrary, is there a rationale for it?

42. Table S1: Do the authors have any explanation regarding the huge deviations in the parameter k_c for the 2-state and 3/4-state model?
