# Supplementary material for: Cross-bridge model for predicting muscle short-range stiffness during movement
Source: PLoS Comput Biol. 2026 Sep 1;22(9):e1014748. doi: 10.1371/journal.pcbi.1014748 (PMC13561430; doi:10.1371/journal.pcbi.1014748)
Supplement: Supplementary file 2 [file pcbi.1014748.s013.pdf]

Review of manuscript PCOMPBIOL-D-26-00059_R2

The authors have revised the manuscript substantially and many of the detailed points raised in the previous round have been addressed. In particular, the manuscript is now clearer regarding the distinction between Hill-type and cross-bridge models, the limitations of the proposed cross-bridge formulations are stated more explicitly, the terminology has been improved, the experimental and computational methods are described in more detail, and many figure, equation, and parameter-description issues have been corrected. I also appreciate the additional discussion of titin, rheopexy, fibre-type effects, and the more careful phrasing of the mechanistic interpretation of cooperative thin- and thick-filament activation. However, I do not think that all conceptual concerns from the previous round have been addressed satisfactorily. In particular, the revised manuscript still treats rather simple Hill-type models as the effective comparison class, although several existing Hill-type or Hill-type-compatible formulations already incorporate nonlinear calcium dynamics, length-dependent calcium sensitivity, activity-dependent force–length behaviour, or short-range-stiffness-like history dependence. Moreover, the alternative electrostatic/interfilament-spacing mechanism raised previously is not discussed in a meaningful way. These two omissions weaken the claim that the present cross-bridge formulation is uniquely required to capture the observed activation-dependent short-range stiffness and thixotropy.

Major concern:
The manuscript still does not make a sufficiently strong effort to improve or enrich the Hill-type comparison models, despite the fact that the previous review round explicitly questioned whether the comparison to Hill-type models was too weak or potentially unfair. The revised manuscript argues that cooperative myofilament activation is hardly implementable in Hill-type models, but I do not find this convincing in its present form. It is true that explicit cross-bridge-state cooperativity is naturally represented in a cross-bridge framework. However, this is not the same as saying that Hill-type models cannot incorporate activation-dependent filament effects, length-dependent calcium sensitivity, or phenomenological history-dependent stiffness. There is a substantial body of literature showing that Hill-type or Hill-type-compatible models can be extended beyond the simple multiplicative structure of activation times force–length times force–velocity. For example, Hatze-type nonlinear activation dynamics include calcium-related effects and have been used to reproduce activity-dependent shifts in optimum muscle length. Kistemaker, van Soest and Bobbert showed that length-dependent Ca^{2+} sensitivity can be incorporated into a Hill-type musculoskeletal framework and can contribute substantially to muscle stiffness, especially at low-to-moderate activation levels (Journal of Biomechanics, DOI: 10.1016/j.jbiomech.2004.08.025). Furthermore, De Groote et al. augmented a Hill-type muscle model with a dynamic short-range-stiffness component and showed that this substantially improved simulated perturbation responses (Journal of Biomechanics, DOI: 10.1016/j.jbiomech.2017.02.008). This latter example is particularly relevant because it directly addresses short-range stiffness in a Hill-type modelling context, and one of the present authors is, to my understanding, also an author of that study. Or am I missing something here? Therefore, the manuscript should either include a more advanced Hill-type comparison model or, at minimum, explicitly acknowledge that the Hill-type models tested here are deliberately simple baseline models and do not exhaust the possible Hill-type modelling space. The current phrasing risks overstating the conclusion that Hill-type models as a class cannot capture activation-dependent short-range stiffness or history dependence.

Medium concern:
The revised manuscript still does not adequately address the alternative electrostatic/interfilament-spacing mechanism raised in the previous review. Rockenfeller, Günther and Hooper proposed an electrophysical sarcomere model in which constant-volume geometry, length-dependent interfilament spacing, calcium binding, and electrostatic thin–thick filament interactions contribute to the active force–length–activation relation, including activity-dependent shifts in optimum length (Biophysical Journal, DOI: 10.1016/j.bpj.2022.04.019). This mechanism is directly relevant to the present manuscript because the authors interpret activation-dependent short-range stiffness and its history dependence mainly through cooperative thin- and thick-filament activation. The present revision discusses cooperative effects and refers to thick-filament mechanosensing, but it does not clearly explain why the number of force-generating cross-bridges should decrease after shortening at the mechanistic level. In particular, I cannot identify from the Barclay- or Linari-related discussion a concrete mechanistic picture for why shortening per se should alter myosin states or reduce

the number of attached (or attachment-competent) cross-bridges in the way inferred by the model. The electrostatic/interfilament-spacing hypothesis could provide an alternative or complementary explanation. Shortening changes sarcomere geometry and, under volume constancy, may alter radial lattice spacing and hence thin–thick filament electrostatic interactions. This could plausibly affect calcium sensitivity, attachment probability, or the number of attachment-competent cross-bridges without requiring the same mechanistic assumptions as force-dependent thick-filament recruitment. I do not ask the authors to implement this mechanism in the present model, but it should be discussed as a plausible alternative or complementary mechanism, especially because the manuscript makes comparatively strong mechanistic claims about cooperative filament activation.

Minor comments:

1. Line 31: "at a 8 times lower computational cost" should be changed to "at an 8-times lower computational cost" or, preferably, "with an approximately eightfold lower computational cost".
2. Line 42: The Author Summary refers to data from "a single rat soleus muscle fiber", whereas the study uses multiple fibres. This should be changed to "isolated rat soleus muscle fibres" or similar.
3. Line 76: "The detachment is proportional..." should be "The detachment rate is proportional...".
4. Lines 80–85: The manuscript appears to use "disordered-relaxed state", "disordered relaxed state", and "disorder-relaxed state" inconsistently. Please standardise the terminology.
5. Line 153: "could robustly captured" should be "could robustly capture" or "robustly captured".
6. Lines 238–245 (and probably elsewhere): There is still an inconsistency in the notation for the force RMSD. If the hats on σ were removed throughout the manuscript, $\hat{\sigma}_F$ should be changed to σ_F.
7. In the Fig. 5 caption, the 4-state XB coop model is described as "purple", whereas elsewhere it appears to be green. Please check and correct the colour description.
8. Line 281: "high large short-range stiffness reductions" should be "large short-range stiffness reductions".
9. In the Fig. 6 caption, the panel labels appear to end "D", "E", "D". The final "D" should presumably be "F". Also,"Data is shown" should be "Data are shown". In the same caption, please clarify the apparent mixture of model predictions averaged over $n = 7$ fibres and experimental data shown for $n = 11$ fibres.
10. Line 341: "Fig, 10" should presumably be "Fig. 8".
11. Line 354: "resulting in a 8.6 times shorter processing time" should be "resulting in an 8.6-fold reduction in processing time" or "an 8.6-times shorter processing time".
12. Line 385: "Fig. 5-9" should be formatted as "Figs. 5–9" as a range of figures is intended.
13. Line 392 There appears to be an extra closing parenthesis in "movement [52–54])".
14. Lines 431: "which suggest that" should be "which suggests that" if the subject is singular.
15. Lines 446: "which may underly" should be "which may underlie".
16. Line 494: "cooperative myofilaments dynamics" should be "cooperative myofilament dynamics".
17. Lines 525 and many others: Please standardise the spelling of "modeling"/"modelling" throughout the manuscript.
18. Line 700: "cross-bridge ripping" is rather colloquial. Consider replacing it with "strain-induced cross-bridge detachment" or "cross-bridge rupture/detachment".
19. Line 718 and others: Use "log" or "$\log_{10}$" consistently.

20. Line 777 and others: Make parentheses as large as the terms inside.
21. Line 779 and others: Differential operator is still italic in some places.
22. Lines 812–813: "transforms the partial differential equation in ordinary differential equations" should be "transforms the partial differential equation into ordinary differential equations".
23. Line 860: "If forcibly detached state is not included" should be "If the forcibly detached state is not included".
24. Line 876: "one additional parameters was fitted" should be "one additional parameter was fitted".
