# Supplementary material for: Cross-bridge model for predicting muscle short-range stiffness during movement
Source: PLoS Comput Biol. 2026 Sep 1;22(9):e1014748. doi: 10.1371/journal.pcbi.1014748 (PMC13561430; doi:10.1371/journal.pcbi.1014748)
Supplement: Supplementary file 3 [file pcbi.1014748.s014.pdf]

We thank both reviewers for their constructive criticism. In addition to addressing the reviewers' comments, we took the opportunity to refine our analyses and update the wording. None of these changes influenced our results. Literature references are listed at the bottom of this document.

Reviewer 1

This study demonstrates – by evaluating various muscle models against empirical data from rat soleus fibers – that cross-bridge models incorporating cooperative activation of both thin and thick myofilaments can accurately predict the history-dependent reduction of short-range stiffness (SRS) known as muscle thixotropy. The authors found that while traditional Hill-type and simpler crossbridge models fail to capture these dynamics – especially at submaximal activation levels – the inclusion of cooperative dynamics allows the model to match experimental force trajectories across various movement histories. To make these complex models practical for musculoskeletal simulations, the authors implemented a Gaussian-approximated solution method that maintains high accuracy while reducing computational costs significantly compared to traditional discretization methods.

This is an interesting and absolutely relevant approach, mostly new (see below) and thus deserves some attention. The authors have a notable publication history for both the issue of thixotropy and muscle modeling. However, I have several major, medium and minor concerns and questions.

Some of these concerns might simply stem from the fact that I am not an expert in Huxley-type modeling, so the authors may feel free to correct me if I am mistaken in certain assumptions or inferences.

Reviewer

Major concerns:

1. One of my main concerns is the – in my opinion unnecessary and biased – 'Hill-bashing', i.e. showing unfair comparisons of Huxley-type and Hill-type models. To elaborate: First off, the first 'subsection' of the discussion ('State-of-the-art musculoskeletal.... properties') claims that no employed Hill-type model could adequately account for thixotropy. It is not said WHERE (i.e. literature sources) those models were employed. Second, comparing a Hill-type model without serial elasticity is just complete bogus, as I personally know of not a single musculoskeletal model that has no SE in it. Third, why is the Hill-type model compared to 3+ state XB models and not likewise modified to account for thin and thick filament activation?

All in all I plead for the omission of Hill-type models in this manuscript as they add nothing to the story at all, but rather seem to serve as a scapegoat. The enhancement of the Huxley type model is the story here and this is an important story indeed. If the authors would like to keep it still, it would be enough to show that the standard Hill- and standard Huxley model equally fail at capturing thixotropy and move on to improve the latter – or improve both models equally and make fair comparisons.

Reply

Our aim was to identify a muscle model suitable for movement simulations based on its ability to capture muscles' history-dependent resistance to stretch (see line 130–132). Hill-type muscle models are the standard in movement simulations, and estimation of muscle forces in inverse dynamic analyses is often based on Hill-type models without series elasticity. We added the following sentence to the introduction to clarify why we included Hill-type models (line 65-67):

> *"Alongside cross-bridge models, we also tested Hill-type models as a benchmark, as they remain the most commonly employed type of muscle model in musculoskeletal simulations of human movement (Wakeling et al., 2023)"*

In response to subpoint 1, we clarified where Hill-type models with and without series compliance have been employed in the discussion section (line 380-382):

> *"Although more and more movement simulations include series compliance in Hill-type models (Afschrift et al., 2019; De Groote et al., 2016; Dembia et al., 2020; Falisse et al., 2019; Geijtenbeek, 2021; Uchida et al., 2016), it is neglected in static optimization - a popular method for estimating muscle forces during movement (Ao and Fregly, 2024; Michaud et al., 2025; Hammond et al., 2025)). It is also neglected in forward simulation frameworks that aim at maximizing computational efficiency, such as MuJoCO (Todorov, 2012)"*

In response to subpoint 2, we added an explanation on why we included a Hill-type model without SE to introduction section (line 137-140):

> *"These two types were included because some have interpreted Hill-type model series compliance as representing tendons (Millard et al., 2013; Thelen, 2003; Wilson and Lichtwark, 2011; Zajac, 1989) that are absent in the skinned fibers modeled here, while others have interpreted it as representing compliance within muscle fibers themselves (Cole et al., 1996; van den Bogert et al., 1998)"*

In response to subpoint 3, we added an explanation on why we did not add cooperative dynamics to Hill-type models to the revised introduction (line 98-100):

> *"Since cooperative myofilament activation is inherently coupled to cross-bridge cycling, it is not straightforward to add this to phenomenological models lacking cross-bridges."*

Finally, in the revised results and discussion sections, we further emphasized that the standard Huxley and Hill-type models indeed fail equally at capturing short-range stiffness, as correctly noted by the reviewer. Two examples are provided below:

> Line 175-177: *"Hill-type and 2-state XB models equally fail at capturing the history dependence of muscle short-range stiffness at submaximal activation levels"*

> Line 362-364: *"However, to our surprise, modeling actin-myosin interactions did not improve predictions of muscle thixotropy compared to Hill-type models with series elasticity"*

Reviewer
2. Another main concern is the terminology of the XB-model as being "biophysical". In my opinion, it is a phenomenological compartment model that describes the physiologically observed transition between XB states, but offers no mechanistic explanation. I give some examples: How exactly are the myosin heads assumed to connect to actin? The model offers a kinetic constant, but does it offer an explanation, e.g. via Coulomb forces? No. How exactly is the XB supposed to produce forces? Well it does as per observation and measurement, but does the XB model offer a mechanism? No again. The whole method section from Eqn. 1 to Eqn. 23 offers not a single biophysically interpretable formula. I am not arguing against the Huxley-model per se, as some do (e.g. the recent work of Josh E. Baker), but I would urge the authors to acknowledge the limitations of the model as being of roughly the same descriptive nature as Hill's force-velocity relation. This brings me to my next point.

Reply
We acknowledge the reviewer's concern regarding the degree to which the term "biophysical" may be appropriate. We made three types of changes:

1. Throughout the manuscript, we replaced the word "biophysical" with "cross-bridge".

2. Some of the biophysical interpretation of the equations was described rather late in the manuscript, in the "Model parameters" section. We moved these descriptions up, directly following the corresponding equations. We also removed unnecessary repetition.

3. We added a dedicated discussion section paragraph in which the limitations of the employed models are discussed (line 509-531). The first sentence of this paragraph reads:

> *"While the cross-bridge models considered are more mechanistic than Hill-type models, they still entail hugely simplified descriptions of the underlying biophysical processes"*

Reviewer

3. The modeling of cooperativity. I have a rough descriptive idea about cooperative thin filament activation in a sense that Calcium binding to troponin C leads to an easier clearance of tropomyosin along the actin filament (it might be worthwhile to mention that Eqn.˙11 is already a cooperative calcium activation), yet I am not sure about the exact mechanism here. The authors now propose another descriptive idea by that the cooperative thin-filament activation is further enhanced by already formed XBs. This picture makes sense as bound myosin might hinder tropomyosin from sliding back in place (i.e. deactivate), but whether this is mechanistically justified, I am not sure. Is it somehow backed in the literature, besides the cited modeling paper?

Reply

We added a reference to Longyear et al. (2017) here (line 81), describing the molecular basis of thin filament activation and how it is affected by cross-bridge binding .

Reviewer

However, I have some problems with the proposed force-dependent thick-filament activation.
While there seems to be a recent push of that idea (by M. Irving and colleagues who term it 'mechano-sensing'), I personally cannot form a coherent mechanistic picture in my head why simply pulling on the end of myosin should result in the heads to change their state (i.e. leave their ordered state ajar to the myosin backbone). Could the authors provide such a picture or mechanism? Following the literature hint ([19]), I couldn't find any. My personal idea of this observation is that the disorder on the myosin head level stems from changes in electrostatic attraction from the actin – be it that it is moved closer (radial distance decreases during lengthening) or that oppositely-charged binding sites are exposed due to said thin-filament activation (as presented recently by Rockenfeller et al. (2022), DOI 10.1016/j.bpj.2022.04.019). In that regard, the paragraph following line 433 needs to be backed with literature sources.

Reply

We made two types of changes:

1. We replaced this literature reference (previously Campbell et al., 2018) with that of the original publication behind the mechanosensing mechanism and briefly summarize the hypothesized mechanism, i.e. line 82-85:

   > *"This transition is thought to depend on force, as filament stress may break the molecular interactions that stabilize the super-relaxed state, thereby decreasing the relative free energy of the disorder-relaxed state (Linari et al., 2015)"*.

2. We revised the discussion section paragraph mentioned by the reviewer (line 445-471), clarifying that it describes model predictions rather than statements about mechanisms. Sentences now start with "*Models explain how*" (line 445), "*The 3-state XB coop model predicts that*" (line 461) and "*These computational results suggest that*" (line 470). Literature references are included in paragraphs that describe the employed models (e.g. Linari reference above).

Reviewer

4. Novelty of the presented approach. I am missing a literature review on how muscle thixotropy has been dealt with hitherto. For example, I found a paper by Altman et al. (2015, DOI 10.1371/journal.pone.0121726) or by Ait-Mou et al. (2016, DOI 10.1073/pnas.1516732113). There may

be more. I personally do not now any Hill-type muscle models considering SRS, but a larger body of force enhancement and force depression modeling.

Reply

We added a paragraph to the introduction that reviews literature on modeling muscle thixotropy (line 90-104):

> *"Muscle models may need cooperative myofilament dynamics to capture muscle thixotropy at submaximal activation. While cross-bridge models lacking cooperative dynamics capture the history-dependence of short-range stiffness at maximal calcium activation levels, they cannot reproduce the empirical observation that muscle thixotropy is greatest at low activation levels (Campbell and Moss, 2000; Campbell and Moss, 2002). Considering that cooperative mechanisms explain the activation dependence of muscle's rate of force development (Fitzsimons et al., 2001), they might also explain the activation dependence of muscle thixotropy. Some cross-bridge models incorporate cooperative dynamics, but such models have not been tested for predicting short-range stiffness across a broad range of conditions including activation levels and recovery times (Tanner et al., 2007; Tanner et al., 2012; Campbell, 2014; Fenwick et al., 2017; Campbell et al., 2018; Lakie and Campbell, 2019; Blum et al., 2020; Liu et al., 2024; Simha and Ting, 2024; Altman et al., 2015). Since cooperative myofilament activation is inherently coupled to cross-bridge cycling (Irving, 2017; Lakie and Campbell, 2019; Linari et al., 2015; Longyear et al., 2017), it is not straightforward to add this to phenomenological models lacking cross-bridges. Hill-type models have been extended to better capture muscle thixotropy, but these models require the movement history to be set manually and do not describe the effect of recovery time and muscle activation on muscle short-range stiffness (Shue and Crago, 1998; Willaert et al., 2024). Therefore, we assessed whether cross-bridge models with cooperative myofilament activation can capture short-range stiffness and its history dependence across a range of behaviorally relevant conditions."*

We added the Altman paper as suggested by the reviewer. We did not add the Ait-Mou paper because it does not address muscle thixotropy. Because Ait-Mou's experimental results do provide useful insight into the mechanisms underlying force production, we added it to the discussion section (line 517-520):

> *"While evidence for the force dependence of myosin's super-relaxed state is accumulating, the underlying mechanisms remain unclear. Titin may play a role, considering that it can transfer force to myosin and thereby induce structural alterations to the thick filament (Ait-Mou et al., 2016; Squarci et al., 2023)"*

Reviewer

Medium concerns:

1. The overall language of the manuscript is on point. Yet, at some instances, it reads like LLM-assisted writing. Could the authors clarify which parts of the manuscript were written (drafted, proofread, etc) by AI? As the most prominent example, I refer to the first and fourth 'subsections' of the discussion ('State-of-the-art musculoskeletal.... properties' and 'Cooperative myofilament...stiffness"). Here, little to no literature reference is given, most sentences are rather descriptive and some information is given twice, e.g. l.370-71 and l.385 both give a physiological interpretation of the SE as tendon.

Reply

We added more literature reference to the first subsection of the discussion section, and omitted unnecessary repetition or description. We clarified that the fourth subsection is describing model predictions rather than statements about mechanisms (see response to earlier comment). We did not use AI in any part of the manuscript.

Reviewer

2. Lines 59-60 and 84-85: I do not understand why muscle shortening should result in a reduction of the number of cross-bridges? And why should it result in lesser force per cross-bridge? What is the picture behind these statements?

Reply

To answer these questions:

1. Previous data have indicated that fiber stiffness decreases with shortening velocity (Ford et al., 1985), which has been interpreted as arising from a reduced number of bound cross-bridges (Barclay, 2015). We added these two references to the sentence referred to by the reviewer (line 59). Recently, the reduction in number of bound cross-bridge has been attributed to the force dependence of myosin's ADP release rate (e.g., Buonfiglio et al., 2024; Capitanio et al., 2012; Liu et al., 2024). We briefly mention this in the discussion, and added these three references to that discussion section (line 512-514).

2. The lesser force per cross-bridge has been attributed to a decrease in cross-bridge strain due to interfilamentous sliding while the cross-bridge is attached (Ford et al., 1985; Huxley and Simmons, 1971). We added these two references on line 87 and revised Fig. 1 to more clearly illustrate this effect and modified the lines mentioned by the reviewer to add nuance to our statements, replacing "can reduce" with "thought to reduce" and "results in" with "thought to result in".

Reviewer

3. Lines 155 and many more: The authors take into account muscle behavior at submaximal Ca levels. First some semantics: In my view, the term "activation" describes a process, while "activity" would describe the state. Would the authors be d'accord to re-term their "activation" accordingly? Second, and this is not semantics, submaximal activity poses a problem in terms of the force-length relation in that the muscle no longer operates at the optimal (or plateau) region (see lines 809-810), but on the ascending limb. It is well-established experimentally that the force-length relation shifts to longer lengths with decreasing activity. I was wondering, if the XB-model of the authors was able to capture this effect? For Hill-models there are several ways to enforce this behavior, e.g. Hatze-type length-dependent activation dynamics or Lloyd-like activity-dependent FL curves. I could not find any such modeling in the Hill-type models used by the authors before, e.g. De Groote et al. (2016). Overall, I think this missing factor would be important to consider in the modeling of submaximal contractions.

Reply

We replaced "activation" with "activation level" throughout the manuscript.

We acknowledge that optimal fiber length may be greater at submaximal activation level, and that this is an interesting effect. However, it was beyond the scope of this manuscript to model this effect, especially because we could not validate the length dependence of model predictions as we only have measurements at one initial fiber length. That being said, previous research has suggested that length-dependent activation may also arise from the same cooperative myofilament dynamics. We briefly discuss this limitation on line 489-494:

> *"A limitation of the current study is that we did not explicitly model the rightward shift of the force-length relation with lower activation levels (de Tombe et al., 2010; Hatze, 1981; Kawai and Jin, 2021; Kistemaker et al., 2005; Lloyd and Besier, 2003; Terui et al., 2010), here referred to as length-dependent activation. While previous modeling studies have indicated that cooperative myofilament dynamics can yield length-dependent activation (Ait-Mou et al., 2016; Campbell et al., 2018; Kawai and Jin, 2021; Terui et al., 2010), it remains unclear whether a single model can simultaneously capture both phenomena"*

Reviewer

4. Fig 4: the results shown in this figure are not discussed enough, in my opinion, and raise many questions. In panel A, I completely fail to understand the huge variation of relative SRS at zero activity (does activity really have the dimension of a force?). Do the authors have any explanation to offer here? Is it a general uncertainty when stretching a passive muscle? If so, this would query the concept of determining SRS in the first place. Similarly, in panel B, first relative SRS seems to grow with amplitude and then at one amplitude it is less. Are the measurements for the other amplitudes not available here? Panel C seems reasonable. From not being able to see the same points in every panel, I concluded that different experimental boundary conditions were shown in each panel, but the caption does not give me a hint what these would be – contrary to Fig. 5 in which they are clearly stated.

Reply

We assume the reviewer refers to Fig. 6 instead of Fig. 4, because this is where relative SRS is shown. The reviewer correctly observed a large variation of relative SRS in the passive condition. We added the following explanation (line 632-637):

> *"It was challenging to reliably calculate short-range stiffness in passive conditions, where forces were so small that they were of similar magnitude as background noise. The chosen time window therefore was a trade-off between including more data points to improve the reliability of the short-range stiffness estimates, and preventing that force leveled off within the window. The chosen window of 0.01 s was well within the range of previous research (Cui et al., 2008; Horslen et al., 2023; de Vlugt et al., 2011)"*

Muscle activation indeed does not have dimension of force. We revised Fig. 6 and the sentence that explains how activation was defined (line 640-643):

> *"To account for differences in calcium sensitivity between fibers, activation level was defined as the isometric force reached relative to that at pCa 4.5. Four activation categories were used to compute fiber-average results: Passive (<5% activated), Low (5 – 30% activated), Intermediate (30 – 70% activated) and High (>70% activated)."*

The reviewer also correctly observed that short-range stiffness grows with amplitude in some cases (Fig. 6B). Upon further inspection, we found that this was an artefact of how SRS was computed. Specifically, in the reference condition of a few fibers, force started to level off within the 10 ms SRS window, resulting in an overestimation of relative short-range stiffness. Reducing the window size could prevent this, but it would also result in much noisier SRS estimates (see above). We noticed a ~2 ms delay between the SRS time window and the force response, most likely due to the frequency response of the force sensor. We therefore shifted the time window 2 ms forward in time, reducing the artefact. We also added the data at the three intermediate amplitudes, for the two fibers in which these were recorded (black dots, Fig. 6B). These two fibers happened to be the ones in which the artefact was most apparent.

Lastly, we revised the caption of Fig. 6 to clarify which conditions are shown, and indicated the condition that panels have in common with a different symbol (i.e. square instead of circle).

Reviewer

Minor concerns:

1. Fig 1: The second ΔF on the bottom right is not in line with the ΔL above. The last sentence of the caption seems highly speculative, is there any literature backing up this claim?

Reply

We aligned the ΔF's and ΔL's and rewrote the last sentence of the Fig. 1 caption to be less speculative:

> *"When another stretch occurs shortly after shortening, some detached cross-bridges will not have had the time to reattach. This mechanism would explain why force increases less rapidly during the second stretch compared to during the first stretch"*

Reviewer
2. Line 82: Reference [19] is imo not an adequate source to back the claim made here, see also my major concern above.

Reply
We added a reference to Linari et al. (2015) here (line 85), see response to major concern above.

Reviewer
3. Line 124: It is not until the discussion that titin is mentioned, although it might explain thixotrophy already when implemented. The authors themselves give a hint in their reference [19] to the modeling paper of Ait-Mou et al. (2016).

Reply
We revised the part of the discussion section on the potential role of titin in muscle thixotropy, adding the Ait-Mou reference (line 520, see earlier reply). We did not add this reference earlier because the paper does not address muscle thixotropy, but about the Frank-Sterling effect. To the best of our knowledge, there does not exist a titin-based model that captures muscle thixotropy. Simultaneously addressing comments from the other reviewer, we added more discussion on the potential role of titin in muscle's mechanical response to stretch (line 520-525):

> *"Titin may also be important for capturing the velocity-dependent force response to longer stretches than those considered here (Millard et al., 2024; Pinniger et al., 2006; Till et al., 2008; Tomalka et al., 2017; Weidner et al., 2022), rheopexy observed during slower stretches (Altman et al., 2015), and the increase in stiffness over multiple stretch-shortening cycles (Elst et al., 2025). The lack of titin dynamics in the models considered here may in part explain why we still observed deviations between model force trajectories and empirical force trajectories, despite finding relatively good agreement during the short-range stiffness phase"*

Reviewer
4. Lines 132-133: This sentence is odd: One models yields better results than another, if we equip it with equations meant to govern the effect of interest. This seems like an obvious statement, see my main concern #1.

Reply
We clarified why we did not add cooperative dynamics to Hill-type models (line 98-100):

> *"Since cooperative myofilament activation is inherently coupled to cross-bridge cycling (Irving, 2017; Lakie and Campbell, 2019; Linari et al., 2015; Longyear et al., 2017), it is not straightforward to add this to phenomenological models lacking cross-bridges".*

Reviewer
5. Lines 203-205: the closing sentence of this paragraph seems a bit of an overstatement. Just because you enhanced the model in a way that it better fit some data does not necessarily imply the physiological consequences you state here. Compare to the statement in lines 469-470.

Reply
We agree that this was an overstatement and therefore removed this sentence. We merged the remainder of this paragraph with the preceding paragraph.

Reviewer
6. Fig. 4: Some text is hidden behind the graphs, consider replacing it.

Reply
We moved this text to below the boxes, such that it does not interfere with the graph

Reviewer

7. Lines 222, 227 and some more: Reporting p values in the magnitude of 2−50 and below is meaningless, as it lies beyond machine precision in Matlab. I suggest defining some thresholds, e.g. 10−4, below which you speak of highly significant differences. Also, the whole paragraph is hard to read as you report the same values for each overall, conditional and test stretches, with a lot of repetitions. Maybe a table would help here?

Reply

We improved this paragraph's readability, removing most p-values and instead using the threshold suggested by the reviewer (line 238-242):

> *"The root-mean-square deviation between model forces and empirical forces σ_F generally decreased with model complexity (see Fig. 5A, Table 1). Comparing models with increasing complexity, differences in overall σ_F were significant (at $p < 10^{-4}$) between Hill-type models with and without series compliance and between the 2-state XB coop and 3-state XB coop models."*

Reviewer

8. Lines 221, 226 and more: The concept of σF was hard to follow here, because it does not appear prominently in the text, but rather wordy at the end of the method section. From what I understand, it bears the unit of relative force, where normalization was done post-hoc to a certain reference force. While this seems reasonable, it might make some consequences not very surprising. In lines 236-237, I would expect σF to go up with higher Ca concentration (more force) and larger amplitude (more passive force). This fact is not discussed, if I am not mistaken.

Reply

We revised this part of the results, adding a general observation and a brief description of σ_F (line 238-242, see reply to previous comment). In the Fig. 5 caption we further clarified the normalization by adding (line 268-269): "*normalized with respect to the maximal isometric force F_0*". The reviewer correctly points out that the increase in σ_F with activation and amplitude is not very surprising. We acknowledge this in the following sentence (line 245-246):

> *"The increase in σ_F with activation level and amplitude is not that surprising, as conditions with larger activation and amplitude are associated with larger (passive) forces"*

Reviewer

9. Fig. 5: The σ is missing its hat. Also, why are only those bars shown for which 11 samples were taken and not the ones with 3 samples as well?

Reply

We removed the hats of σ throughout the manuscript. We only show the conditions in which 11 samples were taken, because, in our opinion, 3 samples are insufficient to reliably estimate a fiber average.

Reviewer

10. Fig 6 (and others): After trying to zoom in on this figure I found it of poor quality. Are no vector graphic exports available? Also, what confidence intervals are shown with the error bars? 95%? What is the quantitative criterion to label some of them "low HD" and others "high HD"?

Reply

We made three types of changes to address these questions:

1. We now inserted all figures as vector graphics format. It remains possible that during the journal's PDF generation process some quality reduction occurs that is beyond our control.

2. Error bars show standard deviation, we added this information to the Fig. 6 legend.

3. “HD” referred to “history dependence”. We replaced “low and high HD” with “small and large short-range stiffness reductions”, which we find more intuitive. In the methods, it is mentioned how these are defined (line 918-921):

   “Based on the experimental results (Horslen et al., 2023), conditions with large short-range stiffness reductions were defined as those with Low or Intermediate activation levels, AMP > 3% L_0 and RT < 0.5 s. Experimental conditions with small short-range stiffness reductions were defined as the remaining conditions.”

Reviewer
11. Line 344: “little influence” is not a quantitative statement.

Reply
To provide quantification, we revised an earlier sentence in this paragraph (line 345-356):

> *“When using the approximated solution method (black dashed line and black dot, Fig. 8), force error was < 0.01 % F_0, while a single function evaluation only required about 0.000017 s”*

Reviewer
12. Fig. 8: The mid and right column might better be displayed using a semi-log plot?!

Reply
We considered this possibility, but decided to use a linear plot because it is easier to interpret.

Reviewer
13. L. 387: I do not understand how sarcomere heterogeneity may work as a serial elastic element. The elasticity of the myosin heads itself were shown to work as such an element (see for example DOI 10.1016/j.jtbi.2018.07.023).

Reply
The elasticity of the myosin heads is already incorporated in the contractile element, through cross-bridge dynamics. Following Campbell (2016), we added a brief explanation of how sarcomere heterogeneity results in series compliance (line 772-774):

> *“The series element represents compliant structures outside the half-sarcomere that act in series with the cross-bridges, such as those arising from sarcomere heterogeneity - where half-sarcomeres in the middle of the preparation shorten by stretching half-sarcomeres near the ends (Campbell, 2016; Julian and Morgan, 1979)”*

Reviewer
14. L. 392: The first half of this sentence seems superfluous.

Reply
We removed this sentence.

Reviewer
15. Lines 406ff: While I appreciate the Gaussian approximation to fasten up the computation, I could not find any quantitative rationale (e.g. a Wasserstein metric) supporting the claim that it “adequately represents the shape of the actual distribution”.

Reply
Our sole aim was to point out that a Gaussian distribution might be a better approximation in some conditions than in others. We revised this sentence (line 426-428):

"Consequently, for conditions in which a Gaussian shape adequately represents the shape of the actual distribution, simulations with the Gaussian-approximated solution method will mimic those with solution methods based on discretization"

Reviewer
16. Line 437: Reference [8] appears twice?!

Reply
Thanks for spotting this mistake. We replaced this reference with Lakie & Campbell (2019), which we found more appropriate (line 449).

Reviewer
17. Line 482: From what I see, reference [28] indeed includes both titin and cooperative myosin dynamics?!

Reply
Thanks for spotting this mistake. We removed this reference here.

Reviewer
18. Line 577: It should read force-TIME curve. Also, can you provide any rationale for choosing the arbitrary 0.01 s?

Reply
Short-range stiffness is defined as the slope of the force-length, not force-time curve. We made two changes:

1. We revised Fig. 9, adding a new panel B that clarifies how short-range stiffness was calculated from the force-length curve.

2. We added a rationale for the chosen time window (line 632-637, see earlier reply).

Reviewer
19. Line 585-586: "ISI" seems to be copy-pasted from a prior publication and should presumably read "RT" here.

Reply
Thanks for spotting this mistake. In an earlier version, we did use ISI instead of RT to describe this measure. We chose RT because it more accurately reflects the measure. We replaced ISI with RT here (line 644-645).

Reviewer
20. Line 606 and many others: This formula (Eqn. 2) does not appear in the cited reference [19]?!

Reply
Most of this formula (Eqn. 2) does appear in the cited reference, albeit using different symbols. The exception is the partial spatial derivative term (i.e. $\partial n/\partial x \cdot \partial x/\partial t$). The reason is that the cited reference uses what we call the "traditional solution method" (see our paragraph titled "Solution methods based on discretization") in which this term is computed separately. To resolve this, we removed the partial spatial derivative term from Eq. 2, replacing the full temporal derivative (i.e. dn/dt) with the partial temporal derivative term (i.e. $\partial n/\partial t$).

Reviewer
Further, I personally find the use of curly brackets disconcerting, but it may be just me. Consider using larger parentheses (in particular Eqs 12, 20 and 21, and some in the supplement). Also, operators (such as the "d" in dx) should not be written in cursive.

Reply
We replaced curly brackets with square brackets and made all operators non-cursive.

Reviewer
21. Line 611: I failed to decode the term "assumed size of the XB power stroke". Do you mean the mean displacement?

Reply
Before this sentence, we added the following explanatory sentence (line 672-674):

> *"In line with previous cross-bridge models (Campbell et al., 2018; Huxley, 1957; Lemaire et al., 2016; Zahalak, 1981), we assume that cross-bridges instantaneously undergo a power-stroke upon attachment, increasing their strain with step size* d_{ps}*, set to 10 nm here (Newhard et al., 2019)"*

Reviewer
22. Line 625: Are both E1 and E2 necessarily greater than zero?

Reply
E_1 should be positive and E_2 should be negative. Consequently, detachment function g(x) increases with both larger positive and negative strains. There was a typo in Eqn. 6 that we corrected: its exponent should start with a minus sign (i.e. e^{-Ex}). Thus, the signs of E_1 and E_2 cause g(x) to rise with larger negative and positive strains respectively. Table S1 incorrectly indicated positive values for E_2 which we changed to negative values. None of this affected our results because the underlying model code (i.e. on Github) was correct.

Reviewer
23. Line 630: Is there a literature reference for Eq. 7?

Reply
No, because cooperative dynamics have not been combined with a forcibly detached state before. To help the reader find the appropriate references, we added that the forcibly-detached part of the equation (previously Eq. 10, now Eq. 7) is based on the model described by Lombardi & Piazzesi (1990). We switched the order of these equations to make things easier to comprehend.

Reviewer
24. Line 641: The parameter Non is not explained, though it can be inferred from the context.

Reply
We sharpened the definition of Non when introducing it (line 670-671):

> *"*N_{on} *is the fraction of thin filament binding sites that is in a state in which cross-bridge attachment is possible".*

Reviewer
25. Line 658: This is one of the reasons, I would not term the model "biophysical", as the force dependence is descriptively hard-coded in the model without any mechanistic idea behind.

Reply
We agree. As mentioned in a previous reply, we removed the word "biophysical". We also added a brief mentioning of the mechanism that is thought to underly myosin's force dependence (line 735-737):

> *"Parameter k_F specifies the sensitivity of the forward rate to normalized cross-bridge force $\tilde{F}_{CB}$, reflecting the effects of filament stress on the molecular interactions stabilizing the super-relaxed state* (Linari et al., 2015)*"*

Reviewer
26. Eqn. 12: Why is Ca concentration not normalized with respect to the maximal concentration?

Reply
Because our maximal Ca concentration (i.e. pCa 4.5 ≈ 32 μM) does not necessarily correspond to the physiological maximum. In slow-twitch muscle, peak Ca concentrations are typically ~10 μM (Baylor and Hollingworth, 2003), which is much lower than pCa 4.5. Because the choice of normalization factor is somewhat arbitrary, we use the absolute (i.e. non-normalized) Ca concentration instead.

Reviewer
27. Line 677: Is there an estimate of N made available?

Reply
We added the following sentence (line 755-756):

> *"The number of sarcomeres in the fiber N was determined from microscopy. For this dataset, N was equal to 306 ± 78 (mean ± s.d.)".*

Reviewer
28. Line 690: Before the era of machine learning ('softplus'), PE and SE have been modeled using this exact function by Gerald Loeb and his group (see e.g. Brown et al. 1996).

Reply
We added references to Brown et al. (1996a) and Brown et al. (1996b) on line 768.

Reviewer
29. Line 695: Why is the SE force not starting at a slack length like the PE but rather at zero?

Reply
Thanks for spotting this mistake. We changed Lse to (Lse-Lse,0) in this equation (Eq. 20). This did not affect our results, because it was correctly implemented in the underlying code.

Reviewer
30. Lines 701 and 824ff: I do not understand the rationale behind choosing β = 2.

Reply
In the initial submission, β represented the inverse fraction of attached myosin heads relative to the total number of myosin heads during a maximal isometric contraction. Because such an inverse fraction is not very intuitive, we changed the definition of β such that it now corresponds to this fraction itself, not its inverse. We revised the equation (Eq. 22) accordingly, and modified the sentence explaining the definition of β and the chosen value (line 783-786):

"Here, β represents the fraction of attached myosin heads during an isometric contraction at maximal activation relative to the total number of myosin heads. Eq. 22 assures that $F_{CE} = 1$ when the thin filament is maximally activated (i.e. pCa = 4.5) and the fiber is isometric at optimum length. Cross-bridge attachment fraction β was set to 0.5 (Linari et al., 1998)."

Reviewer

31. Line 717: Out of ignorance: What are the "two discretization methods"?

Reply

These were explained in the paragraph titled "Solution methods based on discretization". We changed this sentence such that the reader is provided with more information earlier on, see line 800-804:

"We also compared two types of discretization methods, namely one in which the partial differential equation (i.e., Eq. 1) is discretized with respect to both temporal and spatial dynamics (referred to as "traditional solution method"), and one in which this partial differential equation is transformed in a set of ordinary differential equations thereby separating the temporal and spatial dynamics (referred to as "method of characteristics")".

Reviewer

32. Line 757: Is "interp1" really efficient? Matlab documentary states it requires "more memory and computation time than nearest neighbor". Or do you require continuity?

Reply

Nearest neighbor is not an option here, because we indeed require continuity.

Reviewer

33. Line 781ff: Due to the huge amount of parameters presented here, it would be good to shift Table S1 here and also have a second table, presenting all fixed parameters in a concise manner.

Reply

We agree. We moved the parameter values to a table (Table 2) and also moved fitted values from the supplement (previously Table S1) to the main manuscript (now Table 3).

Reviewer

34. Line 797: Parameter "r" should be "s".

Reply

Thanks for spotting this mistake. We corrected this. The symbol was indeed incorrect, but it should have been Γ_1 (not s).

Reviewer

35. Line 799: What is the literature source for $\phi 1 = 1000\ s{-}1$?

Reply

We added a literature reference, see line 708-709:

"*ϕ_1 was set at 1000 s^{-1}, qualitatively matching a previous model formulation* (Lombardi and Piazzesi, 1990) *in which the reattachment rate constant was ~200 times greater than the regular attachment rate constant."*

Reviewer

36. Line 808: Parameter "h" should be "nH".

Reply
We corrected this, thanks for spotting this mistake.

Reviewer
37. Line 811ff: The parameter kF is not force-dependent!

Reply
We removed the word "force-dependent" when referring to this parameter.

Reviewer
38. Line 822: The FV-relation presented in [36, Eqn S4] looks odd, i.e. a high-parametric log curve. Why not use a Hill-relation and make it adequately activity-dependent?

Reply
We added an explanation for choosing this formulation, see line 869-870:

> *"This formulation was chosen to facilitate parameter estimation through direct collocation"*

Reviewer
39. Line 831: Figure 9 instead of Figure 10?

Reply
We corrected this, thanks for spotting this mistake.

Reviewer
40. Line 834: State which algorithm exactly you were using? From working my way through the Matlab scripts, I assume it is ipopt MUMPS, but the files were not readily executable as you hard-coded local paths on your machine. Could this be fixed? And while I appreciate to have a history of the progress of finding the correct algorithms, it is hard to work one's way through old "test"-files.

Reply
We improved the code on Github, removing local paths and older versions of the scripts. We clarified in the text that we used IPOPT with MUMPS (line 895-896).

Reviewer
41. Line 835: This range is rather arbitrary, is there a rationale for it?

Reply
We clarified this (line 892-893):

> *"We chose 0.0843 s because it corresponds to the duration of the test stretch".*

Reviewer
42. Table S1: Do the authors have any explanation regarding the huge deviations in the parameter kc for the 2-state and 3/4-state model?

Reply
We added the following sentences to the discussion section (line 405-408):

> *"In the 2-state XB coop model, all cooperativity is attributed to the thin filament. This seems to be reflected in the thin filament cooperativity parameter k_c, which was ~4 times higher in this model than in the 3-state and 4-state XB coop models (see Table 3)"*

Reviewer #2

The use of muscle models in biomechanics, medicine, and sports is rapidly expanding. They are increasingly applied to study the neuromechanics of whole-body movement, predict the effects of medical treatments and surgery, assess injury risk in accidents, understand how the body stabilizes movement in response to perturbations, and analyze performance for sports training and clinical diagnostics. A large proportion of modeling studies use classical Hill-type models, which are limited, for example, in predicting force generation during eccentric contractions. In particular, the description of the history-dependent reduction in short-range stiffness after muscle shortening, known as muscle thixotropy, is still insufficiently understood. This study compares different modeling approaches (e.g., Hill-type and biophysical cross-bridge (XB) models) with extensive experimental data from stretch-shortening cycles determined in rat soleus muscle fibers. The authors found that only biophysical models with cooperative interactions between muscle's thin and thick filaments could accurately predict short-range stiffness and its reduction after shortening. Consequently, the study has very high relevance for the microstructural understanding of muscle eccentric as well as the further development of biophysical XB models. Nevertheless, there are some limitations in the description of the methodology, the interpretation of the results, and the discussion of the model limitations, which need improvement.

Reviewer

Major comments

This study presents various biophysical XB modeling approaches that lead to a significantly better description of short-range stiffness, and in particular, the reduction of short-range stiffness depending on contraction history (e.g. 3-state and 4-state XB coop models). These models represent (as emphasized by the authors in the discussion and conclusion) a major step forward for improving model predictions of musculoskeletal systems. However, the models were only validated for predicting relatively small changes in muscle fiber length (approximately 0.1 to 6.8% L0, Fig. 9D). For longer stretches, however, a further, approximately linear increase in force occurs (Tomalka et al. 2017, Till et al. 2008), which cannot be explained by the presented models. It is likely that this increase in force can be explained by the force development of an activation-dependent titin spring. Model approaches with an activation-dependent titin spring (which, while not focusing on describing the short-range stiffness), can reproduce linear force development (after the short-range stiffness phase) at long stretches (Till et al. 2008). Thus, the comparison of model predictions with and without an activation-dependent titin spring shows a significantly better prediction of the linear force development from experiments (Millard et al. 2024). While the discussion mentions the importance of titin for describing residual force enhancement (i.e., the isometric force increase after stretch), titin also has a crucial influence on the dynamic eccentric force response (Pinniger et al. 2006) and on the change in the eccentric force response over repeated SSCs (Elst et al. 2025). To enable model predictions over a larger working range (i.e., also at larger stretches), models must be extended to include this additional mechanism (titin-actin interaction). This requires further discussion.

Tomalka et al. (2017). The active force-length relationship is invisible during extensive eccentric contractions in skinned skeletal muscle fibres. Proc Biol Sci 284.

Till et al. (2008). Characterization of isovelocity extension of activated muscle: a Hill-type model for eccentric contractions and a method for parameter determination. J Theor Biol 255, 176-187.

Millard et al. (2024). A benchmark of muscle models to length changes great and small. J Mech Behav Biomed Mater 160, 106740.

Pinniger et al. (2006). Crossbridge and non-crossbridge contributions to tension in lengthening rat muscle: force-induced reversal of the power stroke. J Physiol 573, 627-643.

Elst et al. (2025). Consecutive SSCs increase the SSC effect in skinned rat muscle fibres. Pflugers Arch 477, 873-888.

Response

We revised the discussion section paragraph in which titin is discussed to more clearly reflect the shortcomings of the biophysical models considered here and underscore the need for a model that includes titin (line 509-531), adding the mentioned references. The two sentences most relevant to this specific comment are (line 520-525):

> *"Titin may also be important for capturing the velocity-dependent force response to longer stretches than those considered here (Millard et al., 2024; Pinniger et al., 2006; Till et al., 2008; Tomalka et al., 2017; Weidner et al., 2022), rheopexy observed during slower stretches (Altman et al., 2015), and the increase in stiffness over multiple stretch-shortening cycles (Elst et al., 2025). The lack of titin dynamics in the models considered here may in part explain why we still observed deviations between model force trajectories and empirical force trajectories, despite finding relatively good agreement during the short-range stiffness phase"*

Reviewer

Furthermore, rapid stretches lead to forcibly detached XBs, defined as give (Flitney et al. 1978, Choi et al. 2010, Weidner et al. 2022). Although a "give" does not occur at the applied strain rates and amplitudes (e.g., Fig. 2), it is still interesting to consider whether the XB model with the 'forcibly detached' cross-bridge state is fundamentally capable of reproducing this eccentric behavior. This should at least be discussed.

Flitney et al. (1978). Cross-bridge detachment and sarcomere 'give' during stretch of active frog's muscle. J Physiol 276, 449-465.

Choi et al. (2010). Calcium-activated force of human muscle fibers following a standardized eccentric contraction. Am J Physiol Cell Physiol 299, C1409-1417.

Weidner et al. (2022). How velocity impacts eccentric force generation of fully activated skinned skeletal muscle fibers in long stretches. J Appl Physiol (1985) 133, 223-233.

Reply

We added a supplementary figure (Fig. S7) to show model force predictions at faster strain rates, and a brief mentioning of this in the discussion section (line 474-480):

> *"To our surprise, the model with a forcibly-detached state did not yield improved predictions of the plateau phase of the force response. This could be because the stretches considered here were relatively slow (0.45 L_0/s) and brief (< 0.1 s). Indeed, when evaluating model predictions for faster and longer stretches, differences between models with and without a forcibly detached state were larger (Fig. S7). In response to faster stretches, forces of the 4-state XB coop model had smaller overshoot and undershoot (sometimes referred to as "yield" (Cole et al., 1996; Zahalak, 1981) or "give" (Flitney and Hirst, 1978; Weidner et al., 2022)) than those of the 3-state XB coop model"*

The model with forcibly detached state has less "give" because forcibly-detached cross-bridges re-attach rapidly. In contrast, in the model without a forcibly detached state, re-attachment is relatively slow. Hence, in the latter model, fewer cross-bridges are attached during fast stretches, causing "give". We consider a detailed discussion on "give" behavior beyond the scope of this manuscript.

Reviewer

What is the unloaded shortening velocity of the fibers and the relative strain rate (% vmax) of the fibers during eccentric ramps? This is important because Altman et al. (2015) showed that activated rabbit psoas fibers behave thixotropically at high-frequency cycling, with stiffness decreasing over time, but rheopectically at low frequencies (<1 Hz), with stiffness increasing. Furthermore, short-range stiffness is also dependent on the strain rate (Weidner et al. 2022, Pinniger et al. 2026). What influence do you expect at other strain rates?

Altman et al. (2015). Thixotropy and rheopexy of muscle fibers probed using sinusoidal oscillations. PLoS One 10, e0121726.

Pinniger et al. (2006). Crossbridge and non-crossbridge contributions to tension in lengthening rat muscle: force-induced reversal of the power stroke. J Physiol 573, 627-643.

Weidner et al. (2022). How velocity impacts eccentric force generation of fully activated skinned skeletal muscle fibers in long stretches. J Appl Physiol (1985) 133, 223-233.

Reply
We added the following sentence to the methods (line 590-591):

> *"All stretches had a strain rate of 0.45 L_0/s, corresponding to ~35% of the expected unloaded shortening velocity of these fibers (Widrick et al., 1997)."*

A typical length ramp in our experiment had a total duration of 0.1686 s (i.e., 0.0843 s up and 0.0843 down), which is similar to the period of the high-frequency perturbation at which Altman observed thixotropy (0.1 s). We added a brief mentioning of the potential role of titin in rheopexy (see reply to an earlier comment), also referring to the velocity-dependent force response to longer stretches mentioned by the reviewer. We added the following sentence to acknowledge that our models cannot capture viscous effects mentioned by the reviewer (line 514-516):

> *"But other aspects of the cross-bridge models are still somewhat simplistic and descriptive. For example, our models assume that cross-bridges behave as linear springs, while muscle's mechanical response to stretch has a viscous component (Weidner et al., 2022)."*

Reviewer
Minor comments
To what extent do fiber type-dependent differences in XB and muscle properties (Sieck et al.1998, Weidner et al. 2024, Walklate et al. 2016) play a role in modeling and model predictions?

Sieck et al. (1998). Cross-bridge cycling kinetics, actomyosin ATPase activity and myosin heavy chain isoforms in skeletal and smooth respiratory muscles. Comp Biochem Physiol B Biochem Mol Biol 119, 435-450.

Weidner et al. (2024). Impact of lengthening velocity on the generation of eccentric force by slow-twitch muscle fibers in long stretches. Pflugers Arch 476, 1517-1527.

Walklate et al. (2016). Myosin isoforms and the mechanochemical cross-bridge cycle. J Exp Biol 219, 168-174.

Reply
Fiber type-dependent differences in cross-bridge and muscle properties indeed affect model predictions. For example, in our cross-bridge models, fast-twitch fibers would be associated with faster cross-bridge cycling rates. We briefly discuss these effects (line 501-508):

> *"While fibers in our dataset were predominantly slow-twitch, muscle thixotropy has also been observed in fast-twitch fibers. Fast-twitch fibers are typically associated with faster cross-bridge cycling rates, and faster short-range stiffness recovery times(Campbell and Moss, 2000; Campbell and Moss, 2002). In fast-twitch fibers, more cross-bridges are thought to be attached at any given time, potentially explaining why these fibers have both larger forces and higher metabolic rates when normalized for cross-sectional area (Hill et al., 2026). Future modeling studies could explore the extent to which cooperative myofilament dynamics can simultaneously explain multiple aspects of muscle mechanics and energetics across different fiber types."*

In the methods, we added more information on fiber type (line 583-584):

> *"Fibers had a rate of tension regeneration of less than 2 s^{-1}, consistent with that of slow-twitch fibers (Campbell and Moss, 2002)"*

Reviewer
The description of the experiments on muscle fibers needs improvement. It is clear that experiments from a previous study (Horslen et al. 2023) were used. Nevertheless, for better understanding the experimental data, it is important to describe the execution of the experiments in this manuscript in more detail, e.g., at what temperature the experiments were carried out, how old and heavy the rats were, what sex they were, etc.

Reply
While we do not know the age and mass of the rats, we do know that they were adult female Sprague–Dawley rats. We added this information (line 581) and also added more information on preparation and temperature (line 584-586):

> *"Permeabilized muscle fiber bundles were stored in a glycerol solution at −20°C for no more than 1 month prior to use. A mean temperature of 22 °C was maintained during the experiments"*

Reviewer
L474: The first physiologically motivated biophysical modeling approach to describe history dependence using an activation-dependent titin spring was presented by Rode in 2009 and should be added to the list of models for describing force enhancement (L 474).

Rode et al. (2009). Titin-induced force enhancement and force depression: a 'sticky-spring' mechanism in muscle contractions? J Theor Biol 259, 350-360.

Reply
We added this reference (line 498).

Reviewer
L 527: You performed 80-196 tests per fiber. How was it ensured that the fiber would not be damaged?

Reply
We added the following information (line 596-601):

> *"To reduce the chance of early run-down, the stretch-shorten trials at pCa 4.5 were performed last. The pCa 4.5 conditions of two fibers were excluded because the isometric force at the onset of the stretch-shortening trials was less than 50% of that during an initial reference trial at the same calcium activation level, indicating run-down. Averaged over the remaining trials, isometric force at stretch onset was within 1% of that measured during the reference trials indicating that the fiber was still intact"*

Reviewer
L446: SRX should be defined. (probably super-relaxed is meant)

Reply
That is correct. We replaced SRX with super-relaxed throughout the manuscript.

Literature references

Afschrift, M., van Deursen, R., De Groote, F. and Jonkers, I. (2019). Increased use of stepping strategy in response to medio-lateral perturbations in the elderly relates to altered reactive tibialis anterior activity. *Gait Posture* **68**, 575–582.

Ait-Mou, Y., Hsu, K., Farman, G. P., Kumar, M., Greaser, M. L., Irving, T. C. and de Tombe, P. P. (2016). Titin strain contributes to the Frank-Starling law of the heart by structural rearrangements of both thin- and thick-filament proteins. *Proc. Natl. Acad. Sci. U. S. A.* **113**, 2306–2311.

Altman, D., Minozzo, F. C. and Rassier, D. E. (2015). Thixotropy and rheopexy of muscle fibers probed using sinusoidal oscillations. *PloS One* **10**, e0121726.

Ao, D. and Fregly, B. J. (2024). Comparison of synergy extrapolation and static optimization for estimating multiple unmeasured muscle activations during walking. *J. NeuroEngineering Rehabil.* **21**, 194.

Barclay, C. J. (2015). Energetics of contraction. *Compr. Physiol.* **5**, 961–995.

Baylor, S. M. and Hollingworth, S. (2003). Sarcoplasmic reticulum calcium release compared in slow-twitch and fast-twitch fibres of mouse muscle. *J. Physiol.* **551**, 125–138.

Blum, K. P., Campbell, K. S., Horslen, B. C., Nardelli, P., Housley, S. N., Cope, T. C. and Ting, L. H. (2020). Diverse and complex muscle spindle afferent firing properties emerge from multiscale muscle mechanics. *eLife* **9**, e55177.

Brown, I. E., Scott, S. H. and Loeb, G. E. (1996a). Mechanics of feline soleus: II. Design and validation of a mathematical model. *J. Muscle Res. Cell Motil.* **17**, 221–233.

Brown, I. E., Liinamaa, T. L. and Loeb, G. E. (1996b). Relationships between range of motion, lo, and passive force in five strap-like muscles of the feline hind limb. *J. Morphol.* **230**, 69–77.

Buonfiglio, V., Pertici, I., Marcello, M., Morotti, I., Caremani, M., Reconditi, M., Linari, M., Fanelli, D., Lombardi, V. and Bianco, P. (2024). Force and kinetics of fast and slow muscle myosin determined with a synthetic sarcomere-like nanomachine. *Commun. Biol.* **7**, 361.

Campbell, K. S. (2014). Dynamic coupling of regulated binding sites and cycling myosin heads in striated muscle. *J. Gen. Physiol.* **143**, 387–399.

Campbell, K. S. (2016). Compliance Accelerates Relaxation in Muscle by Allowing Myosin Heads to Move Relative to Actin. *Biophys. J.* **110**, 661–668.

Campbell, K. S. and Moss, R. L. (2000). A thixotropic effect in contracting rabbit psoas muscle: prior movement reduces the initial tension response to stretch. *J. Physiol.* **525**, 531–548.

Campbell, K. S. and Moss, R. L. (2002). History-dependent mechanical properties of permeabilized rat soleus muscle fibers. *Biophys. J.* **82**, 929–943.

Campbell, K. S., Janssen, P. M. L. and Campbell, S. G. (2018). Force-Dependent Recruitment from the Myosin Off State Contributes to Length-Dependent Activation. *Biophys. J.* **115**, 543–553.

Capitanio, M., Canepari, M., Maffei, M., Beneventi, D., Monico, C., Vanzi, F., Bottinelli, R. and Pavone, F. S. (2012). Ultrafast force-clamp spectroscopy of single molecules reveals load dependence of myosin working stroke. *Nat. Methods* **9**, 1013–1019.

Cole, G. K., van den Bogert, A. J., Herzog, W. and Gerritsen, K. G. (1996). Modelling of force production in skeletal muscle undergoing stretch. *J. Biomech.* **29**, 1091–1104.

Cui, L., Perreault, E. J., Maas, H. and Sandercock, T. G. (2008). Modeling short-range stiffness of feline lower hindlimb muscles. *J. Biomech.* **41**, 1945–1952.

De Groote, F., Kinney, A. L., Rao, A. V. and Fregly, B. J. (2016). Evaluation of Direct Collocation Optimal Control Problem Formulations for Solving the Muscle Redundancy Problem. *Ann. Biomed. Eng.* **44**, 2922–2936.

de Tombe, P. P., Mateja, R. D., Kittipong-Tachampa, Mou, Y. A., Farman, G. P. and Irving, T. C. (2010). Myofilament Length Dependent Activation. *J. Mol. Cell. Cardiol.* **48**, 851–858.

de Vlugt, E., van Eesbeek, S., Baines, P., Hilte, J., Meskers, C. G. M. and de Groot, J. H. (2011). Short range stiffness elastic limit depends on joint velocity. *J. Biomech.* **44**, 2106–2112.

Dembia, C. L., Bianco, N. A., Falisse, A., Hicks, J. L. and Delp, S. L. (2020). OpenSim Moco: Musculoskeletal optimal control. *PLOS Comput. Biol.* **16**, e1008493.

Elst, T., Weidner, S., Tomalka, A., Hahn, D., Paternoster, F. K., Seiberl, W. and Siebert, T. (2025). Consecutive SSCs increase the SSC effect in skinned rat muscle fibres. *Pflugers Arch.* **477**, 873–888.

Falisse, A., Serrancolí, G., Dembia, C. L., Gillis, J., Jonkers, I. and De Groote, F. (2019). Rapid predictive simulations with complex musculoskeletal models suggest that diverse healthy and pathological human gaits can emerge from similar control strategies. *J. R. Soc. Interface* **16**, 20190402.

Fenwick, A. J., Wood, A. M. and Tanner, B. C. W. (2017). Effects of cross-bridge compliance on the force-velocity relationship and muscle power output. *PLOS ONE* **12**, e0190335.

Fitzsimons, D. P., Patel, J. R., Campbell, K. S. and Moss, R. L. (2001). Cooperative mechanisms in the activation dependence of the rate of force development in rabbit skinned skeletal muscle fibers. *J. Gen. Physiol.* **117**, 133–148.

Flitney, F. W. and Hirst, D. G. (1978). Cross-bridge detachment and sarcomere "give" during stretch of active frog's muscle. *J. Physiol.* **276**, 449–465.

Ford, L. E., Huxley, A. F. and Simmons, R. M. (1985). Tension transients during steady shortening of frog muscle fibres. *J. Physiol.* **361**, 131–150.

Geijtenbeek, T. (2021). Hyfydy - High Fidelity Dynamics. *Hyfydy*.

Hammond, C. V., Williams, S. T., Vega, M. M., Ao, D., Li, G., Salati, R. M., Pariser, K. M., Shourijeh, M. S., Habib, A. W., Patten, C., et al. (2025). The Neuromusculoskeletal Modeling Pipeline: MATLAB-based model personalization and treatment optimization functionality for OpenSim. *J. NeuroEngineering Rehabil.* **22**, 112.

Hatze, H. (1981). Myoelectric control models of skeletal muscle. *Univ. South Afr. Pretoria* 31–42.

Hill, C., Kalakoutis, M., Arcidiacono, A., Wang, Y., Smith, E., Brunello, E., Fusi, L. and Irving, M. (2026). Distinct distributions of myosin motor conformations during contraction of slow and fast skeletal muscle. *J. Physiol.* **604**, 3843–3874.

Horslen, B. C., Milburn, G. N., Blum, K. P., Simha, S. N., Campbell, K. S. and Ting, L. H. (2023). History-dependent muscle resistance to stretch remains high after small, posturally relevant pre-movements. *J. Exp. Biol.* **226**, jeb245456.

Huxley, A. F. (1957). Muscle structure and theories of contraction. *Prog. Biophys. Biophys. Chem.* **7**, 255–318.

Huxley, A. F. and Simmons, R. M. (1971). Proposed Mechanism of Force Generation in Striated Muscle. *Nature* **233**, 533–538.

Irving, M. (2017). Regulation of Contraction by the Thick Filaments in Skeletal Muscle. *Biophys. J.* **113**, 2579–2594.

Julian, F. J. and Morgan, D. L. (1979). Intersarcomere dynamics during fixed-end tetanic contractions of frog muscle fibres. *J. Physiol.* **293**, 365–378.

Kawai, M. and Jin, J.-P. (2021). Mechanisms of Frank-Starling law of the heart and stretch activation in striated muscles may have a common molecular origin. *J. Muscle Res. Cell Motil.* **42**, 355–366.

Kistemaker, D. A., Van Soest, A. K. J. and Bobbert, M. F. (2005). Length-dependent [Ca2+] sensitivity adds stiffness to muscle. *J. Biomech.* **38**, 1816–1821.

Lakie, M. and Campbell, K. S. (2019). Muscle thixotropy—where are we now? *J. Appl. Physiol.* **126**, 1790–1799.

Lemaire, K. K., Baan, G. C., Jaspers, R. T. and van Soest, A. J. 'Knoek' (2016). Comparison of the validity of Hill and Huxley muscle–tendon complex models using experimental data obtained from rat m. soleus in situ. *J. Exp. Biol.* **219**, 977–987.

Linari, M., Dobbie, I., Reconditi, M., Koubassova, N., Irving, M., Piazzesi, G. and Lombardi, V. (1998). The stiffness of skeletal muscle in isometric contraction and rigor: the fraction of myosin heads bound to actin. *Biophys. J.* **74**, 2459–2473.

Linari, M., Brunello, E., Reconditi, M., Fusi, L., Caremani, M., Narayanan, T., Piazzesi, G., Lombardi, V. and Irving, M. (2015). Force generation by skeletal muscle is controlled by mechanosensing in myosin filaments. *Nature* **528**, 276–279.

Liu, S., Marang, C., Woodward, M., Joumaa, V., Leonard, T., Scott, B., Debold, E., Herzog, W. and Walcott, S. (2024). Modeling Thick Filament Activation Suggests a Molecular Basis for Force Depression. *Biophys. J.* S0006-3495(24)00040–7.

Lloyd, D. G. and Besier, T. F. (2003). An EMG-driven musculoskeletal model to estimate muscle forces and knee joint moments in vivo. *J. Biomech.* **36**, 765–776.

Lombardi, V. and Piazzesi, G. (1990). The contractile response during steady lengthening of stimulated frog muscle fibres. *J. Physiol.* **431**, 141–171.

Longyear, T., Walcott, S. and Debold, E. P. (2017). The molecular basis of thin filament activation: from single molecule to muscle. *Sci. Rep.* **7**, 1822.

Michaud, F., Márquez, G., Giraldez-García, M. A. and Cuadrado, J. (2025). Comparison of subject-specific musculoskeletal model calibration strategies on muscle force and fatigue estimation. *J. NeuroEngineering Rehabil.* **22**, 156.

Millard, M., Uchida, T., Seth, A. and Delp, S. L. (2013). Flexing Computational Muscle: Modeling and Simulation of Musculotendon Dynamics. *J. Biomech. Eng.* **135**, 021005–021005.

Millard, M., Stutzig, N., Fehr, J. and Siebert, T. (2024). A benchmark of muscle models to length changes great and small. *J. Mech. Behav. Biomed. Mater.* **160**, 106740.

Newhard, C. S., Walcott, S. and Swank, D. M. (2019). The load dependence of muscle's force-velocity curve is modulated by alternative myosin converter domains. *Am. J. Physiol.-Cell Physiol.* **316**, C844–C861.

Pinniger, G. J., Ranatunga, K. W. and Offer, G. W. (2006). Crossbridge and non-crossbridge contributions to tension in lengthening rat muscle: force-induced reversal of the power stroke. *J. Physiol.* **573**, 627–643.

Shue, G. H. and Crago, P. E. (1998). Muscle-tendon model with length history-dependent activation-velocity coupling. *Ann. Biomed. Eng.* **26**, 369–380.

Simha, S. N. and Ting, L. H. (2024). Intrafusal cross-bridge dynamics shape history-dependent muscle spindle responses to stretch. *Exp. Physiol.* **109**, 112–124.

Squarci, C., Bianco, P., Reconditi, M., Pertici, I., Caremani, M., Narayanan, T., Horváth, Á. I., Málnási-Csizmadia, A., Linari, M., Lombardi, V., et al. (2023). Titin activates myosin filaments in skeletal muscle by switching from an extensible spring to a mechanical rectifier. *Proc. Natl. Acad. Sci. U. S. A.* **120**, e2219346120.

Tanner, B. C. W., Daniel, T. L. and Regnier, M. (2007). Sarcomere Lattice Geometry Influences Cooperative Myosin Binding in Muscle. *PLOS Comput. Biol.* **3**, e115.

Tanner, B. C. W., Daniel, T. L. and Regnier, M. (2012). Filament Compliance Influences Cooperative Activation of Thin Filaments and the Dynamics of Force Production in Skeletal Muscle. *PLOS Comput. Biol.* **8**, e1002506.

Terui, T., Shimamoto, Y., Yamane, M., Kobirumaki, F., Ohtsuki, I., Ishiwata, S., Kurihara, S. and Fukuda, N. (2010). Regulatory mechanism of length-dependent activation in skinned porcine ventricular muscle: role of thin filament cooperative activation in the Frank-Starling relation. *J. Gen. Physiol.* **136**, 469–482.

Thelen, D. G. (2003). Adjustment of muscle mechanics model parameters to simulate dynamic contractions in older adults. *J. Biomech. Eng.* **125**, 70–77.

Till, O., Siebert, T., Rode, C. and Blickhan, R. (2008). Characterization of isovelocity extension of activated muscle: a Hill-type model for eccentric contractions and a method for parameter determination. *J. Theor. Biol.* **255**, 176–187.

Todorov, E. (2012). MuJoCo: A physics engine for model-based control. *2012 IEEERSJ Int. Conf. Intell. Robots Syst.*

Tomalka, A., Rode, C., Schumacher, J. and Siebert, T. (2017). The active force-length relationship is invisible during extensive eccentric contractions in skinned skeletal muscle fibres. *Proc. Biol. Sci.* **284**, 20162497.

Uchida, T. K., Hicks, J. L., Dembia, C. L. and Delp, S. L. (2016). Stretching Your Energetic Budget: How Tendon Compliance Affects the Metabolic Cost of Running. *PLOS ONE* **11**, e0150378.

van den Bogert, A. J., Gerritsen, K. G. and Cole, G. K. (1998). Human muscle modelling from a user's perspective. *J. Electromyogr. Kinesiol. Off. J. Int. Soc. Electrophysiol. Kinesiol.* **8**, 119–124.

Wakeling, J. M., Febrer-Nafría, M. and De Groote, F. (2023). A review of the efforts to develop muscle and musculoskeletal models for biomechanics in the last 50 years. *J. Biomech.* **155**, 111657.

Weidner, S., Tomalka, A., Rode, C. and Siebert, T. (2022). How velocity impacts eccentric force generation of fully activated skinned skeletal muscle fibers in long stretches. *J. Appl. Physiol.* **133**, 223–233.

Widrick, J. J., Romatowski, J. G., Karhanek, M., Fitts, R. H., and New Collective Author (1997). Contractile properties of rat, rhesus monkey, and human type I muscle fibers. *Am. J. Physiol.* **272**, R34-42.

Willaert, J., Desloovere, K., Campenhout, A. V., Ting, L. H. and Groote, F. D. (2024). Identification of Neural and Non-Neural Origins of Joint Hyper-Resistance Based on a Novel Neuromechanical Model. *IEEE Trans. Neural Syst. Rehabil. Eng. Publ. IEEE Eng. Med. Biol. Soc.* **32**, 1435.

Wilson, A. and Lichtwark, G. (2011). The anatomical arrangement of muscle and tendon enhances limb versatility and locomotor performance. *Philos. Trans. R. Soc. B Biol. Sci.* **366**, 1540–1553.

Zahalak, G. I. (1981). A distribution-moment approximation for kinetic theories of muscular contraction. *Math. Biosci.* **55**, 89–114.

Zajac, F. E. (1989). Muscle and tendon Properties models scaling and application to biomechanics and motor. *Crit. Rev. Biomed. Eng.* **17**, 359–411.
