# Supplementary material for: Cross-bridge model for predicting muscle short-range stiffness during movement
Source: PLoS Comput Biol. 2026 Sep 1;22(9):e1014748. doi: 10.1371/journal.pcbi.1014748 (PMC13561430; doi:10.1371/journal.pcbi.1014748)
Supplement: Supplementary file 4 [file pcbi.1014748.s015.pdf]

Reviewer 1
The authors have revised the manuscript substantially and many of the detailed points raised in the previous round have been addressed. In particular, the manuscript is now clearer regarding the distinction between Hill-type and cross-bridge models, the limitations of the proposed cross-bridge formulations are stated more explicitly, the terminology has been improved, the experimental and computational methods are described in more detail, and many figure, equation, and parameter description issues have been corrected. I also appreciate the additional discussion of titin, rheopexy, fibre-type effects, and the more careful phrasing of the mechanistic interpretation of cooperative thin- and thick-filament activation.

Reply
We are glad to hear that the reviewer appreciated our revision.

Reviewer 1
However, I do not think that all conceptual concerns from the previous round have been addressed satisfactorily. In particular, the revised manuscript still treats rather simple Hill-type models as the effective comparison class, although several existing Hill-type or Hill-type-compatible formulations already incorporate nonlinear calcium dynamics, length-dependent calcium sensitivity, activity-dependent force–length behaviour, or short-range-stiffness-like history dependence. Moreover, the alternative electrostatic/interfilament-spacing mechanism raised previously is not discussed in a meaningful way. These two omissions weaken the claim that the present cross-bridge formulation is uniquely required to capture the observed activation-dependent short-range stiffness and thixotropy.

Reply
We edited the manuscript to clarify why we chose these two Hill-type models as comparison (e.g., line 150-152):

> *"Considering our aim to identify a model suitable for musculoskeletal simulations, we compared cross-bridge models to two Hill-type models commonly employed in musculoskeletal simulations. One of these Hill-type models included series compliance (Afschrift et al., 2019; De Groote et al., 2016; Dembia et al., 2020; Falisse et al., 2019; Geijtenbeek, 2021; Uchida et al., 2016), while the other one did not (Ao and Fregly, 2024; Michaud et al., 2025; Hammond et al., 2025; Todorov, 2012)".*

The reviewer correctly notes that these Hill-type models are rather simple. To address this, we added the following discussion on more complex Hill-type models (line 416-426):

> *"While more complex Hill-type models than those tested here have been developed (e.g., De Groote et al., 2017; Willaert et al., 2024; Lloyd and Besier, 2003; Kistemaker et al., 2005; Haeufle et al., 2014; Kim et al., 2015; Siebert et al., 2018; Rockenfeller et al., 2020; Hussein et al., 2022), it is unlikely that any of these models would have captured short-range stiffness across the conditions tested here. Hill-type model extensions that aimed at better capturing short-range stiffness greatly simplified the influence of movement history, distinguishing only between the presence or absence of prior movement (De Groote et al., 2017; Willaert et al., 2024). Other extensions aimed at capturing other phenomena not described by simple Hill-type models, such as length-dependent activation (Lloyd and Besier, 2003; Kistemaker et al., 2005), non-linear calcium dynamics (Hussein et al., 2022; Kim et al., 2015), transverse loading (Siebert et al., 2018), and serial damping (Haeufle et al., 2014). Yet, forces of these more complex Hill-type models remain uniquely determined by muscle activation, length and velocity. These more complex models therefore cannot account for observed differences in short-range stiffness between two stretches at identical activation, length and velocity (see Figs. 1-4)"*

To address the second issue raised by the reviewer, we added a discussion on the complementary electrostatic/interfilament-spacing mechanism. A portion of this discussion is shown below (line 518-521):

"A limitation of the current study is that we did not explicitly model the rightward shift of the force-length relation with lower activation levels (de Tombe et al., 2010; Hatze, 1981; Kawai and Jin, 2021; Kistemaker et al., 2005; Lloyd and Besier, 2003; Terui et al., 2010), here referred to as length-dependent activation. This phenomenon has recently been attributed to interactions between filament overlap and interfilament spacing (Rockenfeller et al., 2022)"

Both issues (i.e. Hill-type modeling and electrostatic/interfilament-spacing) are addressed in more detail in the respective replies to major and medium concerns below.

Reviewer 1

Major concern:

The manuscript still does not make a sufficiently strong effort to improve or enrich the Hill-type comparison models, despite the fact that the previous review round explicitly questioned whether the comparison to Hill-type models was too weak or potentially unfair. The revised manuscript argues that cooperative myofilament activation is hardly implementable in Hill-type models, but I do not find this convincing in its present form. It is true that explicit cross-bridge-state cooperativity is naturally represented in a cross-bridge framework. However, this is not the same as saying that Hill-type models cannot incorporate activation-dependent filament effects, length-dependent calcium sensitivity, or phenomenological history-dependent stiffness. There is a substantial body of literature showing that Hill-type or Hill-type-compatible models can be extended beyond the simple multiplicative structure of activation times force–length times force–velocity. For example, Hatze-type nonlinear activation dynamics include calcium-related effects and have been used to reproduce activity-dependent shifts in optimum muscle length. Kistemaker, van Soest and Bobbert showed that length-dependent Ca2+ sensitivity can be incorporated into a Hill-type musculoskeletal framework and can contribute substantially to muscle stiffness, especially at low-to-moderate activation levels (Journal of Biomechanics, DOI: 10.1016/j.jbiomech.2004.08.025). Furthermore, De Groote et al. augmented a Hill-type muscle model with a dynamic short-range-stiffness component and showed that this substantially improved simulated perturbation responses (Journal of Biomechanics, DOI: 10.1016/j.jbiomech.2017.02.008). This latter example is particularly relevant because it directly addresses short-range stiffness in a Hill-type modelling context, and one of the present authors is, to my understanding, also an author of that study. Or am I missing something here? Therefore, the manuscript should either include a more advanced Hill-type comparison model or, at minimum, explicitly acknowledge that the Hill-type models tested here are deliberately simple baseline models and do not exhaust the possible Hill-type modelling space. The current phrasing risks overstating the conclusion that Hill-type models as a class cannot capture activation-dependent short-range stiffness or history dependence.

Reply

As mentioned in the reply above, we now explain why we chose these two Hill-type models as the comparison. To refine our claims, we replaced "*Hill-type models*" with "*the Hill-type models*", "*commonly-used Hill-type models*", "*simple Hill-type models*" or "*Hill-type models commonly employed in movement simulations*" throughout the manuscript. In addition, we added a brief discussion on more complex Hill-type models (line 416-426), including those from Kistemaker et al. (2015) and De Groote et al. (2017) mentioned by the reviewer (referred to as KIST15 and GROOT17 below). Below, we briefly explain why neither type of Hill-type model would capture the history dependence of short-range stiffness.

Hill-type modifications such as GROOT17 add model elements that are conditionally engaged. In GROOT17, a 'short-range stiffness' (SRS) element is engaged during lengthening. This SRS element was designed to capture the stiffness of a muscle that had been previously isometric and does not describe thixotropy. Later, we have conditionally (dis-)engaged the SRS element depending on muscle contraction history when simulating lower limb swinging in the pendulum test (e.g., Willaert et al., 2024), but this binary model cannot capture the complex movement history dependence of short-range stiffness that we aimed to describe here. We added the following sentence to clarify this (line 61-63):

> *“Previously proposed extensions only distinguish the absence or presence of prior movement, and therefore cannot describe the complex relationship between muscle short-range stiffness and movement history”*

Hill-type modifications such as KIST15 add complexities while keeping the multiplicative structure of activation × force–length × force–velocity. While activation is length-dependent in KIST15, its force predictions remain uniquely determined by activation, length and velocity. Hence, it could not account for observed short-range stiffness reductions between two consecutive stretches of equal activation, length and velocity. We extended the second paragraph of the discussion to clarify this (line 416-426).

Reviewer 1
Medium concern:
The revised manuscript still does not adequately address the alternative electrostatic/interfilamentspacing mechanism raised in the previous review. Rockenfeller, Gunther and Hooper proposed an electrophysical sarcomere model in which constant-volume geometry, length-dependent interfilament spacing, calcium binding, and electrostatic thin–thick filament interactions contribute to the active force–length–activation relation, including activity-dependent shifts in optimum length (Biophysical Journal, DOI: 10.1016/j.bpj.2022.04.019). This mechanism is directly relevant to the present manuscript because the authors interpret activation-dependent short-range stiffness and its history dependence mainly through cooperative thin- and thick-filament activation. The present revision discusses cooperative effects and refers to thick-filament mechanosensing, but it does not clearly explain why the number of force-generating cross-bridges should decrease after shortening at the mechanistic level. In particular, I cannot identify from the Barclay- or Linari-related discussion a concrete mechanistic picture for why shortening per se should alter myosin states or reduce the number of attached (or attachment-competent) cross-bridges in the way inferred by the model. The electrostatic/interfilament-spacing hypothesis could provide an alternative or complementary explanation. Shortening changes sarcomere geometry and, under volume constancy, may alter radial lattice spacing and hence thin–thick filament electrostatic interactions. This could plausibly affect calcium sensitivity, attachment probability, or the number of attachment-competent crossbridges without requiring the same mechanistic assumptions as force-dependent thick-filament recruitment. I do not ask the authors to implement this mechanism in the present model, but it should be discussed as a plausible alternative or complementary mechanism, especially because the manuscript makes comparatively strong mechanistic claims about cooperative filament activation.

Reply
Rockenfeller et al. provided convincing evidence for an electrostatic/interfilament-spacing mechanism underlying muscle’s force-length relation, including its ascending limb and its rightward shift with lower activation. We clarified that electrostatic/interfilament-spacing and cooperative myofilament activation may be complementary mechanisms (line 523-524):

> *“Thus, interfilament spacing and cooperative myofilament dynamics may be complementary mechanisms underlying muscle force production.”*

Furthermore, we now acknowledge that incorporating electrostatic/interfilament-spacing will be important for capturing forces across a muscle’s entire operating range (line 546-548):

> *“For example, our models do not account for changes in force or calcium sensitivity due to interfilament spacing (Rockenfeller et al., 2022), which will be key to capturing forces across a broader range of length changes than the short-range stretches modelled here.”*

Whilst important for capturing length-dependent activation, electrostatic/interfilament-spacing may not readily explain short-range stiffness reductions observed here, as they occur between consecutive stretches at identical activation, length and velocity. To the best of our knowledge, identical activation, identical length at stretch onset and identical stretch velocity would imply identical radial lattice spacing,

attachment probability, and number of attachment-competent crossbridges predicted by the electrostatic/interfilament-spacing model. Yet, despite identical activation, length at stretch onset and stretch velocity, short-range stiffness is reduced if the muscle has been shortened recently. Electrostatic/interfilament-spacing and cooperative filament activation may thus be complementary rather than alternative mechanisms. We further weakened mechanistic claims on cooperative myofilament activation throughout the manuscript and acknowledged the need to experimentally verify model-based explanations (line 497-499):

> *"It is important to note that these model-based explanations warrant experimental verification. As models inherently simplify reality, it is probable that other mechanisms – not modelled here – also contribute to muscle thixotropy"*

Reviewer 1

Minor comments:

1. Line 31: "at a 8 times lower computational cost" should be changed to "at an 8-times lower computational cost" or, preferably, "with an approximately eightfold lower computational cost".

2. Line 42: The Author Summary refers to data from "a single rat soleus muscle fiber", whereas the study uses multiple fibres. This should be changed to "isolated rat soleus muscle fibres" or similar.

3. Line 76: "The detachment is proportional..." should be "The detachment rate is proportional...".

4. Lines 80–85: The manuscript appears to use "disordered-relaxed state", "disordered relaxed state", and "disorder-relaxed state" inconsistently. Please standardise the terminology.

5. Line 153: "could robustly captured" should be "could robustly capture" or "robustly captured".

6. Lines 238–245 (and probably elsewhere): There is still an inconsistency in the notation for the force RMSD. If the hats on σ were removed throughout the manuscript, ˆσF should be changed to σF .

7. In the Fig. 5 caption, the 4-state XB coop model is described as "purple", whereas elsewhere it appears to be green. Please check and correct the colour description.

8. Line 281: "high large short-range stiffness reductions" should be "large short-range stiffness reductions".

9. In the Fig. 6 caption, the panel labels appear to end "D", "E", "D". The final "D" should presumably be "F". Also, "Data is shown" should be "Data are shown". In the same caption, please clarify the apparent mixture of model predictions averaged over n = 7 fibres and experimental data shown for n = 11 fibres.

10. Line 341: "Fig, 10" should presumably be "Fig. 8".

11. Line 354: "resulting in a 8.6 times shorter processing time" should be "resulting in an 8.6-fold reduction in processing time" or "an 8.6-times shorter processing time".

12. Line 385: "Fig. 5-9" should be formatted as "Figs. 5–9" as a range of figures is intended.

13. Line 392 There appears to be an extra closing parenthesis in "movement [52–54])".

14. Lines 431: "which suggest that" should be "which suggests that" if the subject is singular.

15. Lines 446: "which may underly" should be "which may underlie".

16. Line 494: "cooperative myofilaments dynamics" should be "cooperative myofilament dynamics".

17. Lines 525 and many others: Please standardise the spelling of "modeling"/"modelling" throughout the manuscript.

18. Line 700: "cross-bridge ripping" is rather colloquial. Consider replacing it with "strain induced cross-bridge detachment" or "cross-bridge rupture/detachment".

19. Line 718 and others: Use "log" or "log10" consistently.

20. Line 777 and others: Make parentheses as large as the terms inside.

21. Line 779 and others: Differential operator is still italic in some places.

22. Lines 812–813: "transforms the partial differential equation in ordinary differential equations" should be "transforms the partial differential equation into ordinary differential equations".

23. Line 860: "If forcibly detached state is not included" should be "If the forcibly detached state is not included".

24. Line 876: "one additional parameters was fitted" should be "one additional parameter was fitted".

Reply

We corrected these minor mistakes and thank the reviewer for pointing them out. Small note regarding comment #19**:** "log" and "log10" referred to the natural versus common logarithm (i.e. base e versus base 10). We replaced "log" with "ln" to clarify.

Literature references

Afschrift, M., van Deursen, R., De Groote, F. and Jonkers, I. (2019). Increased use of stepping strategy in response to medio-lateral perturbations in the elderly relates to altered reactive tibialis anterior activity. *Gait Posture* **68**, 575–582.

Ao, D. and Fregly, B. J. (2024). Comparison of synergy extrapolation and static optimization for estimating multiple unmeasured muscle activations during walking. *J. NeuroEngineering Rehabil.* **21**, 194.

De Groote, F., Kinney, A. L., Rao, A. V. and Fregly, B. J. (2016). Evaluation of Direct Collocation Optimal Control Problem Formulations for Solving the Muscle Redundancy Problem. *Ann. Biomed. Eng.* **44**, 2922–2936.

De Groote, F., Allen, J. L. and Ting, L. H. (2017). Contribution of muscle short-range stiffness to initial changes in joint kinetics and kinematics during perturbations to standing balance: A simulation study. *J. Biomech.* **55**, 71–77.

de Tombe, P. P., Mateja, R. D., Kittipong-Tachampa, Mou, Y. A., Farman, G. P. and Irving, T. C. (2010). Myofilament Length Dependent Activation. *J. Mol. Cell. Cardiol.* **48**, 851–858.

Dembia, C. L., Bianco, N. A., Falisse, A., Hicks, J. L. and Delp, S. L. (2020). OpenSim Moco: Musculoskeletal optimal control. *PLOS Comput. Biol.* **16**, e1008493.

Falisse, A., Serrancolí, G., Dembia, C. L., Gillis, J., Jonkers, I. and De Groote, F. (2019). Rapid predictive simulations with complex musculoskeletal models suggest that diverse healthy and pathological human gaits can emerge from similar control strategies. *J. R. Soc. Interface* **16**, 20190402.

Geijtenbeek, T. (2021). Hyfydy - High Fidelity Dynamics. *Hyfydy*.

Haeufle, D. F. B., Günther, M., Bayer, A. and Schmitt, S. (2014). Hill-type muscle model with serial damping and eccentric force-velocity relation. *J. Biomech.* **47**, 1531–1536.

Hammond, C. V., Williams, S. T., Vega, M. M., Ao, D., Li, G., Salati, R. M., Pariser, K. M., Shourijeh, M. S., Habib, A. W., Patten, C., et al. (2025). The Neuromusculoskeletal Modeling Pipeline: MATLAB-based model personalization and treatment optimization functionality for OpenSim. *J. NeuroEngineering Rehabil.* **22**, 112.

Hatze, H. (1981). Myoelectric control models of skeletal muscle. *Univ. South Afr. Pretoria* 31–42.

Hussein, M., Shebl, S., Elnemr, R. and Elkaranshawy, H. (2022). A New Muscle Activation Dynamics Model, That Simulates the Calcium Kinetics and Incorporates the Role of Store-Operated Calcium Entry Channels, to Enhance the Electromyography-Driven Hill-Type Models. *J. Biomech. Eng.* **144**, 011002.

Kawai, M. and Jin, J.-P. (2021). Mechanisms of Frank-Starling law of the heart and stretch activation in striated muscles may have a common molecular origin. *J. Muscle Res. Cell Motil.* **42**, 355–366.

Kim, H., Sandercock, T. G. and Heckman, C. J. (2015). An action potential-driven model of soleus muscle activation dynamics for locomotor-like movements. *J. Neural Eng.* **12**, 046025.

Kistemaker, D. A., Van Soest, A. K. J. and Bobbert, M. F. (2005). Length-dependent [Ca2+] sensitivity adds stiffness to muscle. *J. Biomech.* **38**, 1816–1821.

Lloyd, D. G. and Besier, T. F. (2003). An EMG-driven musculoskeletal model to estimate muscle forces and knee joint moments in vivo. *J. Biomech.* **36**, 765–776.

Michaud, F., Márquez, G., Giraldez-García, M. A. and Cuadrado, J. (2025). Comparison of subject-specific musculoskeletal model calibration strategies on muscle force and fatigue estimation. *J. NeuroEngineering Rehabil.* **22**, 156.

Rockenfeller, R., Günther, M., Stutzig, N., Haeufle, D. F. B., Siebert, T., Schmitt, S., Leichsenring, K., Böl, M. and Götz, T. (2020). Exhaustion of Skeletal Muscle Fibers Within Seconds: Incorporating Phosphate Kinetics Into a Hill-Type Model. *Front. Physiol.* **11**, 306.

Rockenfeller, R., Günther, M. and Hooper, S. L. (2022). Muscle active force-length curve explained by an electrophysical model of interfilament spacing. *Biophys. J.* **121**, 1823–1855.

Siebert, T., Stutzig, N. and Rode, C. (2018). A hill-type muscle model expansion accounting for effects of varying transverse muscle load. *J. Biomech.* **66**, 57–62.

Terui, T., Shimamoto, Y., Yamane, M., Kobirumaki, F., Ohtsuki, I., Ishiwata, S., Kurihara, S. and Fukuda, N. (2010). Regulatory mechanism of length-dependent activation in skinned porcine ventricular muscle: role of thin filament cooperative activation in the Frank-Starling relation. *J. Gen. Physiol.* **136**, 469–482.

Todorov, E. (2012). MuJoCo: A physics engine for model-based control. *2012 IEEERSJ Int. Conf. Intell. Robots Syst.*

Uchida, T. K., Hicks, J. L., Dembia, C. L. and Delp, S. L. (2016). Stretching Your Energetic Budget: How Tendon Compliance Affects the Metabolic Cost of Running. *PLOS ONE* **11**, e0150378.

Willaert, J., Desloovere, K., Campenhout, A. V., Ting, L. H. and Groote, F. D. (2024). Identification of Neural and Non-Neural Origins of Joint Hyper-Resistance Based on a Novel Neuromechanical Model. *IEEE Trans. Neural Syst. Rehabil. Eng. Publ. IEEE Eng. Med. Biol. Soc.* **32**, 1435.
