# Supplementary material for: Cross-bridge model for predicting muscle short-range stiffness during movement
Source: PLoS Comput Biol. 2026 Sep 1;22(9):e1014748. doi: 10.1371/journal.pcbi.1014748 (PMC13561430; doi:10.1371/journal.pcbi.1014748)
Supplement: Supplementary file 5 [file pcbi.1014748.s003.docx]

## S1 Appendix: analytical expressions for the spatial integrals

Because the attachment functions $f\left( x \right)$ and $\phi\left( x \right)$ are gaussian, $C_{\lambda}$ and $\beta_{\lambda}$ can be expressed in terms of model parameters $f_{1}$, $\phi_{1}$ and $w$:

$${C_{0}=f_{1} \atop\begin{aligned} C_{1}=0 \\ C_{2}={2\cdot f}_{1}\cdot w^{2} \end{aligned}}$$

$${\beta_{0}=\phi_{1} \atop\begin{aligned} \beta_{1}=0 \\ \beta_{2}={2\cdot\phi}_{1}\cdot w^{2} \end{aligned}}$$

Because an exponential detachment function $g\left( x \right)$ is used, $\theta_{\lambda}$ can be written in terms of the moments of the cross-bridge distribution $Q_{0}-Q_{2}$, and the model parameters $g_{1}$, $g_{2}$, $E_{1}$ and $E_{2}$:

$$\theta_{0}=\sum_{i=1}^{2} g_{i}\cdot e^{-E_{i}\cdot(p-q^{2}\cdot\frac{E_{i}}{2})}$$

$$\theta_{1}=\sum_{i=1}^{2} g_{i}\cdot e^{-E_{i}\cdot(p-q^{2}\cdot\frac{E_{i}}{2})}\cdot(p-q^{2}\cdot E_{i})$$

$$\theta_{2}=\sum_{i=1}^{2} g_{i}\cdot e^{-E_{i}\cdot(p-q^{2}\cdot\frac{E_{i}}{2})}\cdot{[\left( p-q^{2}\cdot E_{i} \right)}^{2}+q^{2}]$$

Where:

$p=\frac{Q_{1}}{Q_{0}}$ , $q=\sqrt{\frac{Q_{2}}{Q_{0}}-p^{2}}$

$$n\left( x \right)=\frac{Q_{0}}{\sqrt{2\pi{\cdot q}^{2}}}\cdot e^{-\frac{{(x-p)}^{2}}{2{\cdot q}^{2}}}$$

Because the detachment function $\Gamma\left( x \right)$ is a step function, $\Omega_{\lambda}$ can be written in terms of the moments of the cross-bridge distribution $Q_{0}-Q_{2}$, and the model parameters $r$ and $x_{c}$:

$$\Omega_{0}=r\cdot\frac{Q_{0}}{2}\cdot[1-\mathrm{erf} \left( \frac{x_{c}-p}{\sqrt{2\cdot q^{2}}} \right)]$$

$$\Omega_{1}=\Omega_{0}\cdot p-q^{2}\cdot n(x_{c})$$

$$\Omega_{2}=\Omega_{0}\cdot\left( p^{2}+q^{2} \right)-q^{2}\cdot n\left( x_{c} \right)\cdot(p+x_{c})$$

Here, $\mathrm{erf}$ is the error function..
