# Supplementary material for: Cross-bridge model for predicting muscle short-range stiffness during movement
Source: PLoS Comput Biol. 2026 Sep 1;22(9):e1014748. doi: 10.1371/journal.pcbi.1014748 (PMC13561430; doi:10.1371/journal.pcbi.1014748)
Supplement: Peer review history

# Peer review: Decision Letter 0

**DOI:** 10.1371/journal.pcbi.1014748.r001

**Authors:** Dimitrios Vavylonis, Alex Tam

23 Apr 2026

Biophysical model for predicting muscle short-range stiffness during movement

PLOS Computational Biology

Dear Dr. van der Zee,

Thank you for submitting your manuscript to PLOS Computational Biology. After careful consideration, we feel that it has merit but does not fully meet PLOS Computational Biology's publication criteria as it currently stands. Therefore, we invite you to submit a revised version of the manuscript that addresses the points raised during the review process.

Please submit your revised manuscript by Jun 23 2026 11:59PM. If you will need more time than this to complete your revisions, please reply to this message or contact the journal office at ploscompbiol@plos.org. When you're ready to submit your revision, log on to https://www.editorialmanager.com/pcompbiol/ and select the 'Submissions Needing Revision' folder to locate your manuscript file.

Please include the following items when submitting your revised manuscript:

* A letter that responds to each point raised by the editor and reviewer(s). You should upload this letter as a separate file labeled 'Response to Reviewers'. This file does not need to include responses to formatting updates and technical items listed in the 'Journal Requirements' section below.

* A marked-up copy of your manuscript that highlights changes made to the original version. You should upload this as a separate file labeled 'Revised Manuscript with Track Changes'.

* An unmarked version of your revised paper without tracked changes. You should upload this as a separate file labeled 'Manuscript'.

If you would like to make changes to your financial disclosure, competing interests statement, or data availability statement, please make these updates within the submission form at the time of resubmission. Guidelines for resubmitting your figure files are available below the reviewer comments at the end of this letter

We look forward to receiving your revised manuscript.

Kind regards,

Alex Tam

Academic Editor

PLOS Computational Biology

Dimitrios Vavylonis

Section Editor

PLOS Computational Biology

**Additional Editor Comments:**

Thank you for your patience with this submission, and please find attached the reviewer comments. In your revision, please address all comments and respond to all major and medium comments directly, focusing particularly on enhancing the discussion to situate the study within the literature identified by the reviewers, and on the mechanistic explanations of the cross-bridge model.

**Journal Requirements:**

If the reviewer comments include a recommendation to cite specific previously published works, please review and evaluate these publications to determine whether they are relevant and should be cited. There is no requirement to cite these works unless the editor has indicated otherwise.

1) Please ensure that the CRediT author contributions listed for every co-author are completed accurately and in full.

At this stage, the following Authors/Authors require contributions: Tim J. van der Zee, Kenneth S. Campbell, Friedl De Groote, Gregory N. Milburn, Surabhi N. Simha, and Lena H. Ting. Please ensure that the full contributions of each author are acknowledged in the "Add/Edit/Remove Authors" section of our submission form.

The list of CRediT author contributions may be found here: https://journals.plos.org/ploscompbiol/s/authorship#loc-author-contributions

2) We ask that a manuscript source file is provided at Revision. Please upload your manuscript file as a .doc, .docx, .rtf or .tex. If you are providing a .tex file, please upload it under the item type u2018LaTeX Source Fileu2019 and leave your .pdf version as the item type u2018Manuscriptu2019.

3) Please upload all main figures as separate Figure files in .tif or .eps format. For more information about how to convert and format your figure files please see our guidelines:

https://journals.plos.org/ploscompbiol/s/figures

4) We have noticed that you have uploaded Supporting Information files, but you have not included a list of legends. Please add a full list of legends for your Supporting Information files after the references list.

5) Please amend your detailed Financial Disclosure statement. This is published with the article. It must therefore be completed in full sentences and contain the exact wording you wish to be published.

1) State the initials, alongside each funding source, of each author to receive each grant. For example: "This work was supported by the National Institutes of Health (####### to AM; ###### to CJ) and the National Science Foundation (###### to AM)."

2) State what role the funders took in the study. If the funders had no role in your study, please state: "The funders had no role in study design, data collection and analysis, decision to publish, or preparation of the manuscript."

3) If any authors received a salary from any of your funders, please state which authors and which funders..

If you did not receive any funding for this study, please simply state: u201cThe authors received no specific funding for this work.u201d

6) Some material included in your submission may be copyrighted. According to PLOS's copyright policy, authors who use figures or other material (e.g., graphics, clipart, maps) from another author or copyright holder must demonstrate or obtain permission to publish this material under the Creative Commons Attribution 4.0 International (CC BY 4.0) License used by PLOS journals. Please closely review the details of PLOS's copyright requirements here: PLOS Licenses and Copyright. If you need to request permissions from a copyright holder, you may use the PLOS Content Copyright Permission Form Please respond directly to this email and provide any known details concerning your material's license terms and permissions required for reuse, even if you have not yet obtained copyright permissions or are unsure of your material's copyright compatibility. Once you have responded and addressed all other outstanding technical requirements, you may resubmit your manuscript within Editorial Manager. Potential Copyright Issues:

i) Figures 1, and 10. Please confirm whether you drew the images / clip-art within the figure panels by hand. If you did not draw the images, please provide (a) a link to the source of the images or icons and their license / terms of use; or (b) written permission from the copyright holder to publish the images or icons under our CC BY 4.0 license. Alternatively, you may replace the images with open source alternatives. See these open source resources you may use to replace images / clip-art: - https://commons.wikimedia.org - https://openclipart.org/.

**Reviewers' comments:**

Reviewer's Responses to Questions

**Comments to the Authors:**

**Please note here if the review is uploaded as an attachment.**

Reviewer #1: See attached pdf.

Reviewer #2: The use of muscle models in biomechanics, medicine, and sports is rapidly expanding. They are increasingly applied to study the neuromechanics of whole-body movement, predict the effects of medical treatments and surgery, assess injury risk in accidents, understand how the body stabilizes movement in response to perturbations, and analyze performance for sports training and clinical diagnostics. A large proportion of modeling studies use classical Hill-type models, which are limited, for example, in predicting force generation during eccentric contractions. In particular, the description of the history-dependent reduction in short-range stiffness after muscle shortening, known as muscle thixotropy, is still insufficiently understood. This study compares different modeling approaches (e.g., Hill-type and biophysical cross-bridge (XB) models) with extensive experimental data from stretch-shortening cycles determined in rat soleus muscle fibers. The authors found that only biophysical models with cooperative interactions between muscle's thin and thick filaments could accurately predict short-range stiffness and its reduction after shortening. Consequently, the study has very high relevance for the microstructural understanding of muscle eccentric as well as the further development of biophysical XB models. Nevertheless, there are some limitations in the description of the methodology, the interpretation of the results, and the discussion of the model limitations, which need improvement.

Major comments:

This study presents various biophysical XB modeling approaches that lead to a significantly better description of short-range stiffness, and in particular, the reduction of short-range stiffness depending on contraction history (e.g. 3-state and 4-state XB coop models). These models represent (as emphasized by the authors in the discussion and conclusion) a major step forward for improving model predictions of musculoskeletal systems. However, the models were only validated for predicting relatively small changes in muscle fiber length (approximately 0.1 to 6.8% L0, Fig. 9D). For longer stretches, however, a further, approximately linear increase in force occurs (Tomalka et al. 2017, Till et al. 2008), which cannot be explained by the presented models. It is likely that this increase in force can be explained by the force development of an activation-dependent titin spring. Model approaches with an activation-dependent titin spring (which, while not focusing on describing the short-range stiffness), can reproduce linear force development (after the short-range stiffness phase) at long stretches (Till et al. 2008). Thus, the comparison of model predictions with and without an activation-dependent titin spring shows a significantly better prediction of the linear force development from experiments (Millard et al. 2024). While the discussion mentions the importance of titin for describing residual force enhancement (i.e., the isometric force increase after stretch), titin also has a crucial influence on the dynamic eccentric force response (Pinniger et al. 2006) and on the change in the eccentric force response over repeated SSCs (Elst et al. 2025). To enable model predictions over a larger working range (i.e., also at larger stretches), models must be extended to include this additional mechanism (titin-actin interaction). This requires further discussion.

Tomalka et al. (2017). The active force-length relationship is invisible during extensive eccentric contractions in skinned skeletal muscle fibres. Proc Biol Sci 284.

Till et al. (2008). Characterization of isovelocity extension of activated muscle: a Hill-type model for eccentric contractions and a method for parameter determination. J Theor Biol 255, 176-187.

Millard et al. (2024). A benchmark of muscle models to length changes great and small. J Mech Behav Biomed Mater 160, 106740.

Pinniger et al. (2006). Crossbridge and non-crossbridge contributions to tension in lengthening rat muscle: force-induced reversal of the power stroke. J Physiol 573, 627-643.

Elst et al. (2025). Consecutive SSCs increase the SSC effect in skinned rat muscle fibres. Pflugers Arch 477, 873-888.

Furthermore, rapid stretches lead to forcibly detached XBs, defined as give (Flitney et al. 1978, Choi et al. 2010, Weidner et al. 2022). Although a "give" does not occur at the applied strain rates and amplitudes (e.g., Fig. 2), it is still interesting to consider whether the XB model with the 'forcibly detached' cross-bridge state is fundamentally capable of reproducing this eccentric behavior. This should at least be discussed.

Flitney et al. (1978). Cross-bridge detachment and sarcomere 'give' during stretch of active frog's muscle. J Physiol 276, 449-465.

Choi et al. (2010). Calcium-activated force of human muscle fibers following a standardized eccentric contraction. Am J Physiol Cell Physiol 299, C1409-1417.

Weidner et al. (2022). How velocity impacts eccentric force generation of fully activated skinned skeletal muscle fibers in long stretches. J Appl Physiol (1985) 133, 223-233.

What is the unloaded shortening velocity of the fibers and the relative strain rate (% vmax) of the fibers during eccentric ramps? This is important because Altman et al. (2015) showed that activated rabbit psoas fibers behave thixotropically at high-frequency cycling, with stiffness decreasing over time, but rheopectically at low frequencies (<1 Hz), with stiffness increasing. Furthermore, short-range stiffness is also dependent on the strain rate (Weidner et al. 2022, Pinniger et al. 2026). What influence do you expect at other strain rates?

Altman et al. (2015). Thixotropy and rheopexy of muscle fibers probed using sinusoidal oscillations. PLoS One 10, e0121726.

Pinniger et al. (2006). Crossbridge and non-crossbridge contributions to tension in lengthening rat muscle: force-induced reversal of the power stroke. J Physiol 573, 627-643.

Weidner et al. (2022). How velocity impacts eccentric force generation of fully activated skinned skeletal muscle fibers in long stretches. J Appl Physiol (1985) 133, 223-233.

Minor comments:

To what extent do fiber type-dependent differences in XB and muscle properties (Sieck et al.1998, Weidner et al. 2024, Walklate et al. 2016) play a role in modeling and model predictions?

Sieck et al. (1998). Cross-bridge cycling kinetics, actomyosin ATPase activity and myosin heavy chain isoforms in skeletal and smooth respiratory muscles. Comp Biochem Physiol B Biochem Mol Biol 119, 435-450.

Weidner et al. (2024). Impact of lengthening velocity on the generation of eccentric force by slow-twitch muscle fibers in long stretches. Pflugers Arch 476, 1517-1527.

Walklate et al. (2016). Myosin isoforms and the mechanochemical cross-bridge cycle. J Exp Biol 219, 168-174.

The description of the experiments on muscle fibers needs improvement. It is clear that experiments from a previous study (Horslen et al. 2023) were used. Nevertheless, for better understanding the experimental data, it is important to describe the execution of the experiments in this manuscript in more detail, e.g., at what temperature the experiments were carried out, how old and heavy the rats were, what sex they were, etc.

L474: The first physiologically motivated biophysical modeling approach to describe history dependence using an activation-dependent titin spring was presented by Rode in 2009 and should be added to the list of models for describing force enhancement (L 474).

Rode et al. (2009). Titin-induced force enhancement and force depression: a 'sticky-spring' mechanism in muscle contractions? J Theor Biol 259, 350-360.

L 527: You performed 80-196 tests per fiber. How was it ensured that the fiber would not be damaged?

L446: SRX should be defined. (probably super-relaxed is meant)

**********

**Have the authors made all data and (if applicable) computational code underlying the findings in their manuscript fully available?**

The PLOS Data policy requires authors to make all data and code underlying the findings described in their manuscript fully available without restriction, with rare exception (please refer to the Data Availability Statement in the manuscript PDF file). The data and code should be provided as part of the manuscript or its supporting information, or deposited to a public repository. For example, in addition to summary statistics, the data points behind means, medians and variance measures should be available. If there are restrictions on publicly sharing data or code —e.g. participant privacy or use of data from a third party—those must be specified.

Reviewer #1: Yes

Reviewer #2: Yes

**********

PLOS authors have the option to publish the peer review history of their article (what does this mean?). If published, this will include your full peer review and any attached files.

If you choose “no”, your identity will remain anonymous but your review may still be made public.

**Do you want your identity to be public for this peer review?**  For information about this choice, including consent withdrawal, please see our Privacy Policy.

Reviewer #1: No

Reviewer #2: No

[NOTE: If reviewer comments were submitted as an attachment file, they will be attached to this email and accessible via the submission site. Please log into your account, locate the manuscript record, and check for the action link "View Attachments". If this link does not appear, there are no attachment files.]

**Figure resubmission:**

After uploading your figures to PLOS’s NAAS tool - https://ngplosjournals.pagemajik.ai/artanalysis, NAAS will process the files provided and display the results in the "Uploaded Files" section of the page as the processing is complete. If the uploaded figures meet our requirements (or NAAS is able to fix the files to meet our requirements), the figure will be marked as "fixed" above. If NAAS is unable to fix the files, a red "failed" label will appear above. When NAAS has confirmed that the figure files meet our requirements, please download the file via the download option, and include these NAAS processed figure files when submitting your revised manuscript.

**Reproducibility:**

To enhance the reproducibility of your results, we recommend that authors of applicable studies deposit laboratory protocols in protocols.io, where a protocol can be assigned its own identifier (DOI) such that it can be cited independently in the future. Additionally, PLOS ONE offers an option to publish peer-reviewed clinical study protocols. Read more information on sharing protocols at https://plos.org/protocols?utm_medium=editorial-email&utm_source=authorletters&utm_campaign=protocols

Attachment Submitted filename: Review_PCOMPBIOL-D-26-00059.pdf

# Author comment: Author response to Decision Letter 1

**DOI:** 10.1371/journal.pcbi.1014748.r002

26 Jun 2026

Attachment Submitted filename: reply_to_reviewer_clean.pdf

# Peer review: Decision Letter 1

**DOI:** 10.1371/journal.pcbi.1014748.r003

**Authors:** Dimitrios Vavylonis, Alex Tam

31 Jul 2026

PCOMPBIOL-D-26-00059R1

Cross-bridge model for predicting muscle short-range stiffness during movement

PLOS Computational Biology

Dear Dr. van der Zee,

Thank you for submitting your manuscript to PLOS Computational Biology. After careful consideration, we feel that it has merit but does not fully meet PLOS Computational Biology's publication criteria as it currently stands. Therefore, we invite you to submit a revised version of the manuscript that addresses the points raised during the review process.

Please submit your revised manuscript by Sep 30 2026 11:59PM. If you will need more time than this to complete your revisions, please reply to this message or contact the journal office at ploscompbiol@plos.org. When you're ready to submit your revision, log on to https://www.editorialmanager.com/pcompbiol/ and select the 'Submissions Needing Revision' folder to locate your manuscript file.

Please include the following items when submitting your revised manuscript:

* A letter that responds to each point raised by the editor and reviewer(s). You should upload this letter as a separate file labeled 'Response to Reviewers'. This file does not need to include responses to formatting updates and technical items listed in the 'Journal Requirements' section below.

* A marked-up copy of your manuscript that highlights changes made to the original version. You should upload this as a separate file labeled 'Revised Manuscript with Track Changes'.

* An unmarked version of your revised paper without tracked changes. You should upload this as a separate file labeled 'Manuscript'.

If you would like to make changes to your financial disclosure, competing interests statement, or data availability statement, please make these updates within the submission form at the time of resubmission. Guidelines for resubmitting your figure files are available below the reviewer comments at the end of this letter.

As the corresponding author, your ORCID iD is verified in the submission system and will appear in the published article. PLOS supports the use of ORCID, and we encourage all coauthors to register for an ORCID iD and use it as well. Please encourage your coauthors to verify their ORCID iD within the submission system before final acceptance, as unverified ORCID iDs will not appear in the published article. Only the individual author can complete the verification step; PLOS staff cannot verify ORCID iDs on behalf of authors.

We look forward to receiving your revised manuscript.

Kind regards,

Alex Tam

Academic Editor

PLOS Computational Biology

Dimitrios Vavylonis

Section Editor

PLOS Computational Biology

**Additional Editor Comments (if provided):**

Dear authors, the reviewers were satisfied with many of the changes made in the revised version of this submission. One reviewer has a few outstanding comments, so I invite you to address these in a second revision.

**Journal Requirements:**

If the reviewer comments include a recommendation to cite specific previously published works, please review and evaluate these publications to determine whether they are relevant and should be cited. There is no requirement to cite these works unless the editor has indicated otherwise.

1) Please amend your detailed Financial Disclosure statement. This is published with the article. It must therefore be completed in full sentences and contain the exact wording you wish to be published.

i) State the initials, alongside each funding source, of each author to receive each grant. For example: "This work was supported by the National Institutes of Health (####### to AM; ###### to CJ) and the National Science Foundation (###### to AM)."

ii) State what role the funders took in the study. If the funders had no role in your study, please state: "The funders had no role in study design, data collection and analysis, decision to publish, or preparation of the manuscript."

iii) If any authors received a salary from any of your funders, please state which authors and which funders..

If you did not receive any funding for this study, please simply state: u201cThe authors received no specific funding for this work.u201d

**Reviewers' comments:**

Reviewer's Responses to Questions

**Comments to the Authors:**

**Please note here if the review is uploaded as an attachment.**

Reviewer #1: See attached pdf

Reviewer #2: Thank you for the thorough revision of the manuscript. The authors have carefully considered and addressed all comments raised in the first round of review. Overall, the quality and scientific insight of the manuscript have improved substantially. The limitations of the biophysical model are now discussed more clearly, particularly with regard to the omission of titin. In addition, the presentation of the model's force predictions at higher strain rates in the supplementary material (Fig. S7) is both interesting and informative, providing valuable context for the reader. I have no further comments on the manuscript.

**********

**Have the authors made all data and (if applicable) computational code underlying the findings in their manuscript fully available?**

The PLOS Data policy requires authors to make all data and code underlying the findings described in their manuscript fully available without restriction, with rare exception (please refer to the Data Availability Statement in the manuscript PDF file). The data and code should be provided as part of the manuscript or its supporting information, or deposited to a public repository. For example, in addition to summary statistics, the data points behind means, medians and variance measures should be available. If there are restrictions on publicly sharing data or code —e.g. participant privacy or use of data from a third party—those must be specified.

Reviewer #1: Yes

Reviewer #2: Yes

**********

PLOS authors have the option to publish the peer review history of their article (what does this mean?). If published, this will include your full peer review and any attached files.

If you choose “no”, your identity will remain anonymous but your review may still be made public.

**Do you want your identity to be public for this peer review?**  For information about this choice, including consent withdrawal, please see our Privacy Policy.

Reviewer #1: No

Reviewer #2: No

[NOTE: If reviewer comments were submitted as an attachment file, they will be attached to this email and accessible via the submission site. Please log into your account, locate the manuscript record, and check for the action link "View Attachments". If this link does not appear, there are no attachment files.]

**Figure resubmission:**

**Reproducibility:**

To enhance the reproducibility of your results, we recommend that authors of applicable studies deposit laboratory protocols in protocols.io, where a protocol can be assigned its own identifier (DOI) such that it can be cited independently in the future. Additionally, PLOS ONE offers an option to publish peer-reviewed clinical study protocols. Read more information on sharing protocols at https://plos.org/protocols?utm_medium=editorial-email&utm_source=authorletters&utm_campaign=protocols

Attachment Submitted filename: Review_PCOMPBIOL-D-26-00059_R2.pdf

# Author comment: Author response to Decision Letter 2

**DOI:** 10.1371/journal.pcbi.1014748.r004

14 Aug 2026

Attachment Submitted filename: reply_to_reviewer_round2_clean.pdf

# Editor report: Decision Letter 2

**DOI:** 10.1371/journal.pcbi.1014748.r005

**Authors:** Dimitrios Vavylonis, Alex Tam

20 Aug 2026

Dear Mr. van der Zee,

We are pleased to inform you that your manuscript 'Cross-bridge model for predicting muscle short-range stiffness during movement' has been provisionally accepted for publication in PLOS Computational Biology.

Before your manuscript can be formally accepted you will need to complete some formatting changes, which you will receive in a follow up email. A member of our team will be in touch with a set of requests.

Please note that your manuscript will not be scheduled for publication until you have made the required changes, so a swift response is appreciated.

IMPORTANT: The editorial review process is now complete. PLOS will only permit corrections to spelling, formatting or significant scientific errors from this point onwards. Requests for major changes, or any which affect the scientific understanding of your work, will cause delays to the publication date of your manuscript.

Should you, your institution's press office or the journal office choose to press release your paper, you will automatically be opted out of early publication. We ask that you notify us now if you or your institution is planning to press release the article. All press must be co-ordinated with PLOS.

Thank you again for supporting Open Access publishing; we are looking forward to publishing your work in PLOS Computational Biology.

Best regards,

Alex Tam

Academic Editor

PLOS Computational Biology

Dimitrios Vavylonis

Section Editor

PLOS Computational Biology

***********************************************************

Thank you for your thorough responses to the reviewer comments throughout this process.

# Editor report: Acceptance letter

**DOI:** 10.1371/journal.pcbi.1014748.r006

**Authors:** Dimitrios Vavylonis, Alex Tam

PCOMPBIOL-D-26-00059R2

Cross-bridge model for predicting muscle short-range stiffness during movement

Dear Dr van der Zee,

I am pleased to inform you that your manuscript has been formally accepted for publication in PLOS Computational Biology. Your manuscript is now with our production department and you will be notified of the publication date in due course.

The corresponding author will soon be receiving a typeset proof for review, to ensure errors have not been introduced during production. Please review the PDF proof of your manuscript carefully, as this is the last chance to correct any errors. Please note that major changes, or those which affect the scientific understanding of the work, will likely cause delays to the publication date of your manuscript.

Soon after your final files are uploaded, unless you have opted out, the early version of your manuscript will be published online. The date of the early version will be your article's publication date. The final article will be published to the same URL, and all versions of the paper will be accessible to readers.

For Research, Software, and Methods articles, you will receive an invoice from PLOS for your publication fee after your manuscript has reached the completed accept phase. If you receive an email requesting payment before acceptance or for any other service, this may be a phishing scheme. Learn how to identify phishing emails and protect your accounts at https://explore.plos.org/phishing.

Thank you again for supporting PLOS Computational Biology and open-access publishing. We are looking forward to publishing your work!

With kind regards,

Sharmila Kamatchi

PLOS Computational Biology | Carlyle House, Carlyle Road, Cambridge CB4 3DN | United Kingdom ploscompbiol@plos.org | Phone +44 (0) 1223-442824 | ploscompbiol.org | @PLOSCompBiol
